# Serotonin (5‐Hydroxytryptamine): Metabolism, Signaling, Biological Functions, Diseases, and Emerging Therapeutic Opportunities

**DOI:** 10.1002/mco2.70383

**Published:** 2025-09-09

**Authors:** Yuxin Zhang, Nan Wang, Louqian Zhang, Yan Zhuang, Qilei Xin, Xiaosong Gu, Chunping Jiang, Junhua Wu

**Affiliations:** ^1^ State Key Laboratory of Pharmaceutical Biotechnology Department of General Surgery Nanjing Drum Tower Hospital The Affiliated Hospital of Medical School Medical School Nanjing University Nanjing China; ^2^ Jinan Microecological Biomedicine Shandong Laboratory Building 1, Jinan Medical and Health Science and Technology Innovation Industrial Park Jinan City Shandong China; ^3^ Dezhou Hospital of Traditional Chinese Medicine Dezhou China; ^4^ “Nanjing University‐Gulou” Joint Laboratory of AI and Healthcare Bigdata National Institute of Healthcare Data Science at Nanjing University School of Life Sciences Jiangsu Key Laboratory of Molecular Medicine Nanjing University Nanjing China; ^5^ Department of Hepatobiliary and Pancreatic Surgery The Second Affiliated Hospital of Fujian Medical University Fuzhou China

**Keywords:** 5‐hydroxytryptamine (5‐HT), 5‐HT receptor (5‐HTR), serotonin, signaling pathways, therapeutic potential, tumor immune microenvironment

## Abstract

Serotonin (5‐hydroxytryptamine; 5‐HT) is an evolutionarily conserved monoamine neurotransmitter that plays critical roles in various physiological systems, functioning as a neurotransmitter, hormone, and paracrine signaling molecule. This review synthesizes current research on 5‐HT metabolism (biosynthesis, transport, and degradation), 5‐HT receptor‐mediated signaling pathways (seven receptor families and 14 subtypes), and broad biological functions of 5‐HT. We emphasize the roles of 5‐HT in both health and disease, with a particular focus on its emerging significance in the tumor immune microenvironment. Studies have shown that dysregulated 5‐HT signaling is associated with various pathological conditions, including functional gastrointestinal disorders, psychiatric diseases, metabolic disorders, and cancer progression. Notably, this review describes novel mechanisms by which 5‐HT modulates tumor immunity, including its effects on macrophage polarization, dendritic cell function, T cell activity, and PD‐L1 expression, and it explores the therapeutic potential of targeting 5‐HT‐associated pathways. Promising therapeutic strategies that target 5‐HT include combining selective serotonin reuptake inhibitors with immune checkpoint inhibitors, inhibiting key metabolic enzymes (e.g., Tph1 and MAO‐A), and developing receptor subtype‐specific agents (e.g., 5‐HT_7_R antagonists). These findings position the 5‐HT system as a pivotal target for next‐generation precision therapeutics across multiple disease domains.

## Graphical abstract

This article reviews the roles of 5‐HT metabolism, 5‐HT receptors, and their related signaling pathways in normal physiology and various diseases, and explores their potential value in disease treatment, providing a reference basis for research and clinical treatment in related fields.

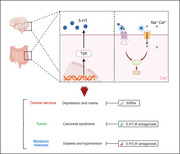

## 1 Introduction

Serotonin (5‐hydroxytryptamine; 5‐HT) research spans the fields of neurobiology, pharmacology, immunology, and systems physiology. 5‐HT functions as a key neurotransmitter, hormonal regulator, and paracrine signaling molecule, and it is widely involved in the regulation of multiple physiological systems [1, 2]. Moreover, 5‐HT is currently recognized as a central modulator of central nervous system (CNS) functions, such as mood, cognition, and pain perception, and peripheral processes, including gastrointestinal (GI) motility, cardiovascular homeostasis, platelet aggregation, and immune responses [3, 4]. In addition to its fundamental biological roles, 5‐HT has attracted enormous attention because of its clinical relevance in central and peripheral disorders, including depression, irritable bowel syndrome (IBS), neurodegenerative diseases, metabolism, and other syndromes [5, 6, 7, 8]. Additionally, recent studies have highlighted the increasingly prominent role of 5‐HT in tumor immunomodulation [9]. The important role of 5‐HT in many diseases emphasizes its emerging potential for use as a therapeutic target and places this field at the forefront of translational research.

There is broad consensus in the scientific community that 5‐HT functions through a complex network of metabolic pathways and receptor subtypes; at least 14 subtypes of 5‐HT receptors (5‐HTRs) have been identified to date, and each subtype mediates distinct physiological effects [10, 11]. Although the pathways that are involved in 5‐HT metabolism and 5‐HTR signaling are well characterized, their functional roles and mechanisms in the context of disease remain controversial [12, 13]. For example, the 5‐HT transporter (SERT) has been particularly well studied, and it is the cornerstone of current antidepressant pharmacotherapy [14]. However, the precise mechanisms underlying the role of 5‐HT in mood disorders, as well as the contribution of peripheral 5‐HT to systemic diseases such as metabolic syndrome or cardiovascular dysfunction, are still under debate [15]. Emerging evidence that 5‐HT plays dual roles in tumorigenesis has further complicated the traditional CNS‐centric understanding of 5‐HT, highlighting the need for integrative and systems research [16].

In light of these developments, this review aims to provide a comprehensive and up‐to‐date summary of the current knowledge about 5‐HT, including its metabolism, receptor‐mediated signaling, physiological functions, and pathophysiological roles [17, 18, 19]. Here, we systematically organize the advances in understanding how 5‐HT signaling modulates disease progression and how these pathways may be leveraged in the development of multitarget therapeutic strategies [20, 21, 22, 23, 24]. By incorporating insights from the fields of biology, immunoregulation, and molecular signaling, this review highlights the integrative role of the 5‐HT system in the pathophysiology of multiple organ systems, offering a comprehensive perspective for future mechanistic research.

This review is organized as follows. First, we provide an overview of 5‐HT metabolism and summarize its physiological roles. Next, we describe 5‐HTR‐mediated signaling cascades and receptor–receptor interactions. Then, we assess currently available therapeutic strategies and their limitations. Subsequent sections explore recent advances in 5‐HT‐related research in the fields of cancer, functional GI disorders, neuropsychiatric diseases, and metabolic syndromes, with a focus on its immunomodulatory functions. We further evaluate the potential of 5‐HT to be used as a biomarker of disease. Finally, we discuss emerging directions for targeting the 5‐HT system, including strategies for multitarget modulation.

## 2 5‐HT Metabolism and Physiological Functions

5‐HT is widely distributed in both the CNS and peripheral tissues, where it performs diverse regulatory functions in the nervous, endocrine, and immune systems [1]. In recent years, advances in molecular biology, neuroscience, and immunology have further elucidated the mechanisms underlying 5‐HT synthesis and metabolism as well as its roles in the physiological regulation of multiple systems.

### 2.1 Biosynthesis, Transport, and Degradation

**FIGURE 1:**
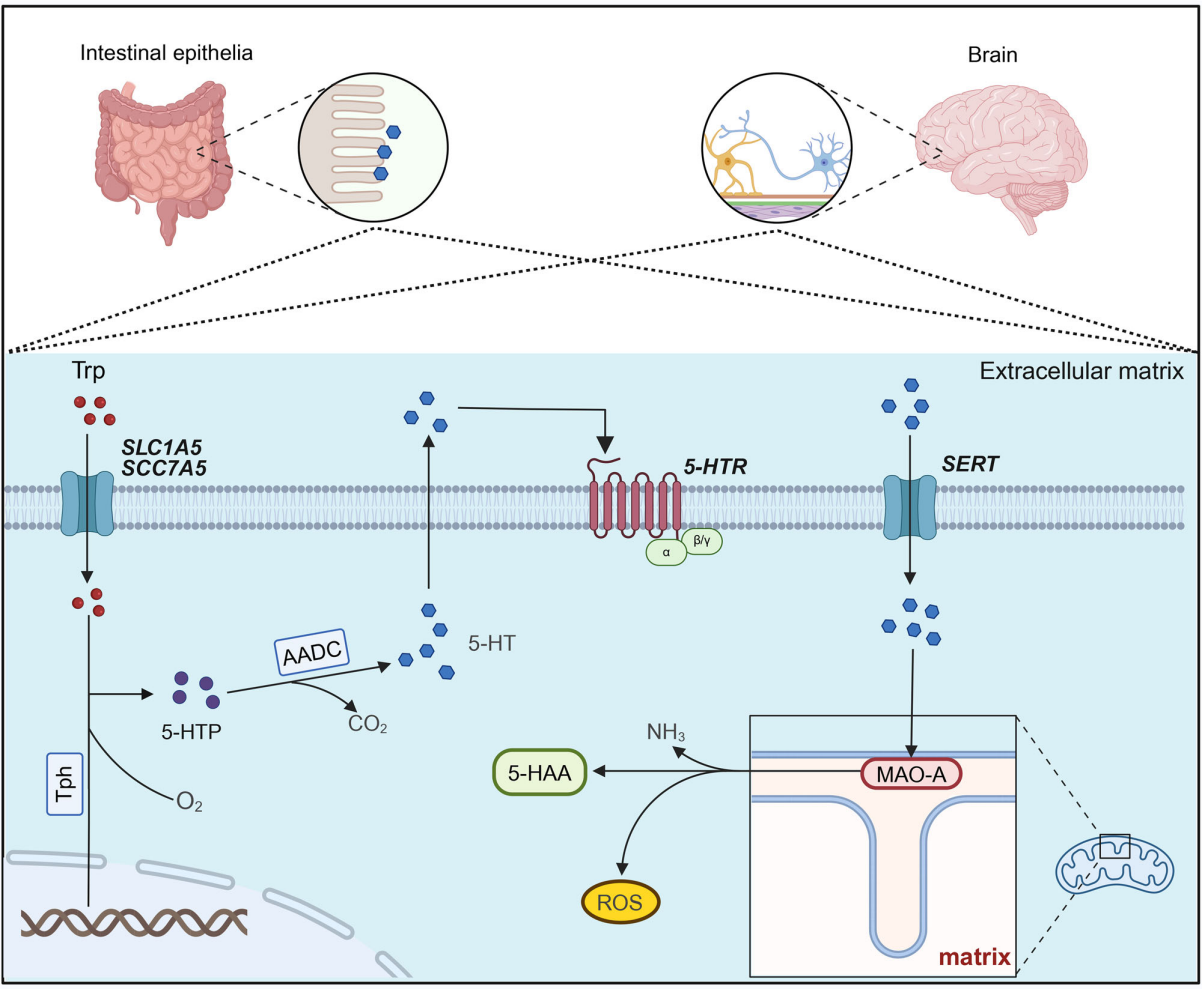
Pathways of serotonin (5‐HT) metabolism. Within the cytoplasm of cells, tryptophan hydroxylase (Tph) catalyzes the conversion of l‐tryptophan (Trp) to 5‐hydroxytryptophan (5‐HTP), which is then converted to 5‐HT via aromatic amino acid decarboxylase (AADC). After crossing the cell membrane via exocytosis, 5‐HT binds to a 5‐HT receptor (5‐HTR). Extracellular 5‐HT is transported into the cytoplasm via the 5‐HT transporter (SERT). 5‐HT can be catabolized into 5‐hydroxyindoleacetic acid (5‐HAA), NH_3_, and reactive oxygen species (ROS) by mitochondrial monoamine oxidase A (MAO‐A). *Abbreviations*: SLC1A5: solute carrier family 1 member 5; SCC7A5: solute carrier family 1 member 7.

5‐HT is a substance that exerts a strong vasoconstrictor effect, and it was first isolated from the intestine [1]. Tryptophan is converted into 5‐hydroxytryptophan (5‐HTP) by tryptophan hydroxylase (Tph), and then, 5‐HT is further generated via the action of 5‐HTP decarboxylase [10]. In this biosynthetic process, Tph functions as the rate‐limiting enzyme in 5‐HT synthesis. There are two isoforms of Tph in the body: Tph1 and Tph2 [25]. Tph1 is located mainly in the chromaffin cells of the intestine, where it produces approximately 95% of the total 5‐HT molecules throughout the body, whereas Tph2 is expressed primarily in the brain within the CNS, where it synthesizes approximately 5% of the total 5‐HT molecules throughout the body [26]. Since 5‐HT cannot cross the blood‒brain barrier, the 5‐HT that is synthesized by Tphs is confined to either the peripheral or CNS [27] (Figure 1). Notably, in addition to the classical synthesis pathway, the cytochrome P450 enzyme system (especially CYP2D6) may participate in the synthesis of 5‐HT via the O‐demethylation of 5‐methoxytryptamine [28]

In the circulatory system, the 5‐HT that is synthesized by the body is stored in platelets through SERT, where it performs important physiological functions [26, 29]. 5‐HT plays a physiological role by binding to specific receptors and then quickly dissociating from these receptors [11]. The dissociated 5‐HT is then reabsorbed into platelets or other related cells by SERT, resulting in a loss of its physiological activity, thereby terminating its effects in the body [30, 31].

In tissues, platelets, and other related cells, the metabolism of 5‐HT mainly depends on monoamine oxidase A (MAO‐A) in mitochondria, and the function of MAO‐A requires flavin adenine dinucleotide as a cofactor [32, 33]. 5‐HT is metabolized mainly into 5‐hydroxyindoleacetic acid (5‐HIAA) [34]. This metabolic pathway involves the initial oxidation of 5‐HT by MAO‐A to generate the corresponding aldehyde. This aldehyde is then further oxidized by aldehyde dehydrogenase to form 5‐HIAA, which is an indoleacetic acid derivative [34]. 5‐HIAA is subsequently excreted by the kidneys [34]. In addition, there is a minor metabolic pathway by which 5‐HIAL is converted to 5‐hydroxytryptol by acetaldehyde reductase, but this pathway is generally considered nondominant [35]. During the degradation of 5‐HT by MAO‐A, reactive oxygen species (ROS) are also generated, and these ROS may induce oxidative stress in cells, further regulating cellular functions or contributing to pathological responses [16, 36].

### 2.2 Physiological Functions

5‐HT, which is an important neurotransmitter, is widely distributed in various tissues, such as the CNS, GI tract, and platelets [37]. 5‐HT plays key roles in many physiological processes, especially in the CNS and peripheral systems [38]. 5‐HT is involved in mood regulation, cognitive function, sleep, appetite control, and so on, and it plays a crucial role in regulating peripheral systems, such as the cardiovascular and GI systems [38, 39].

The role of 5‐HT in the nervous system is particularly important, since it is involved in the regulation of various functions, such as mood, memory, learning, and pain transmission [40]. Research has shown that changes in 5‐HT levels are closely associated with several mental disorders [40]. 5‐HT deficiency or 5‐HTR dysfunction may lead to symptoms, such as depression and anxiety. Selective serotonin reuptake inhibitors (SSRIs) function by increasing the concentration of 5‐HT in the brain, thereby helping to treat depression and anxiety [41, 42]. In addition, 5‐HT plays dual roles in the perception, regulation and conduction of pain depending on the site of its action and its interaction with different receptors [43]. Peripherally, 5‐HT enhances pain perception by binding to the 5‐HTRs on peripheral nerves [44]. Especially during an inflammatory response, 5‐HT may exacerbate pain by promoting local vasodilation and increasing the excitability of local nerve conduction. In the CNS, the role of 5‐HT is more complex, since in this system, it can both alleviate and intensify pain [45]. 5‐Hydroxytryptaminergic (5‐HTergic) neurons communicate with the spinal thalamic tract through multisynaptic or monosynaptic connections, thereby modulating the transmission of pain signals [46]. Furthermore, the 5‐HT system interacts with other neurotransmitters, such as those in the norepinephrine (NE) and dopamine (DA) systems, to jointly regulate the transmission and modulation of pain [47, 48, 49].

Moreover, 5‐HT not only is involved in the regulation of emotions but also plays important roles in cognitive function, learning and memory [39]. 5‐HTergic neurons primarily project from the raphe nucleus to areas of the brain, such as the hippocampus and prefrontal cortex (PFC), as well as extending descending projections to regions such as the periaqueductal gray, striatum, and amygdala [50]. The function of 5‐HT in areas of the brain such as the hippocampus is considered to be closely related to synaptic plasticity and efficiency of information transmission during learning [49]. 5‐HT regulates hippocampal synaptic connectivity by altering the excitability of intracellular homeostasis and synaptic connections [51], and reduced release of 5‐HT leads to declarative memory dysfunction [52].

Furthermore, 5‐HT plays a crucial role in the peripheral nervous system, particularly in the regulation of GI function, the cardiovascular system, and platelet activity [53]. In the GI tract, 5‐HT regulates gut motility, secretion, and immune responses [54, 55, 56, 57]. An imbalance in the 5‐HT levels in the GI tract may be associated with a variety of GI disorders, such as IBS, constipation, or diarrhea [58, 59, 60]. Moreover, 5‐HT may have complex interactions with the gut microbiota. Indeed, 5‐HT affects the growth and metabolism of gut microbes, thereby indirectly influencing overall gut function and immune responses [61, 62]. Additionally, 5‐HT plays a significant role in the vascular system [63]. 5‐HT regulates vascular smooth muscle to induce vasoconstriction, thereby affecting the regulation of blood pressure [64]. Under normal physiological conditions, 5‐HT helps maintain the balance between blood vessel constriction and dilation, thus participating in the regulation of local blood flow [16]. However, under pathological conditions, the abnormal release or metabolism of 5‐HT may contribute to the development of diseases such as hypertension and arteriosclerosis [16, 65]. Additionally, 5‐HT plays a crucial role in platelets. During the coagulation process, platelets release 5‐HT, which aids in platelet aggregation and thrombus formation, thus contributing to hemostasis [66]. Changes in the concentration of 5‐HT in the blood are closely related to thrombus formation and dissolution, highlighting its significant role in the development of cardiovascular diseases [67, 68].

In addition to its traditional physiological functions, 5‐HT has recently been shown to play important roles in metabolic regulation and immune modulation [69]. Similar to the expression of the classic fasting hormone glucagon, the upregulation of Tph1 expression in the gut leads to a significant increase in 5‐HT levels during fasting [70]. This change effectively promotes the breakdown of fats in adipocytes, providing the necessary substrates for hepatic gluconeogenesis [70]. Furthermore, 5‐HT plays dual roles in inflammatory responses [71]. Research has shown that 5‐HT can modulate the intensity of inflammation by promoting or inhibiting the release of certain cytokines. This function makes 5‐HT a key focus in the study of autoimmune diseases and allergic reactions.

Overall, 5‐HT, which is an important neurotransmitter, not only regulates psychological and physiological functions such as mood, cognition, and sleep in the CNS but also participates in various physiological processes of the peripheral nervous system by regulating the GI tract, blood vessels, and platelet functions. As the understanding of the physiological functions of 5‐HT increases, more drugs that target the 5‐HT system are likely to emerge in the future, potentially leading to significant advances in the treatment of both neurological and peripheral system disorders.

## 3 Signaling Mechanisms

5‐HT plays a wide range of physiological roles in both the central and peripheral nervous systems [38]. 5‐HT must exert its effects through its corresponding receptors. Owing to the diversity of 5‐HTRs, the signaling mechanisms that are mediated by 5‐HT are complex, and different receptors activate distinct signaling pathways. The following is an overview of the signaling mechanisms that are mediated by 5‐HT.

### 3.1 5‐HTRs

**FIGURE 2:**
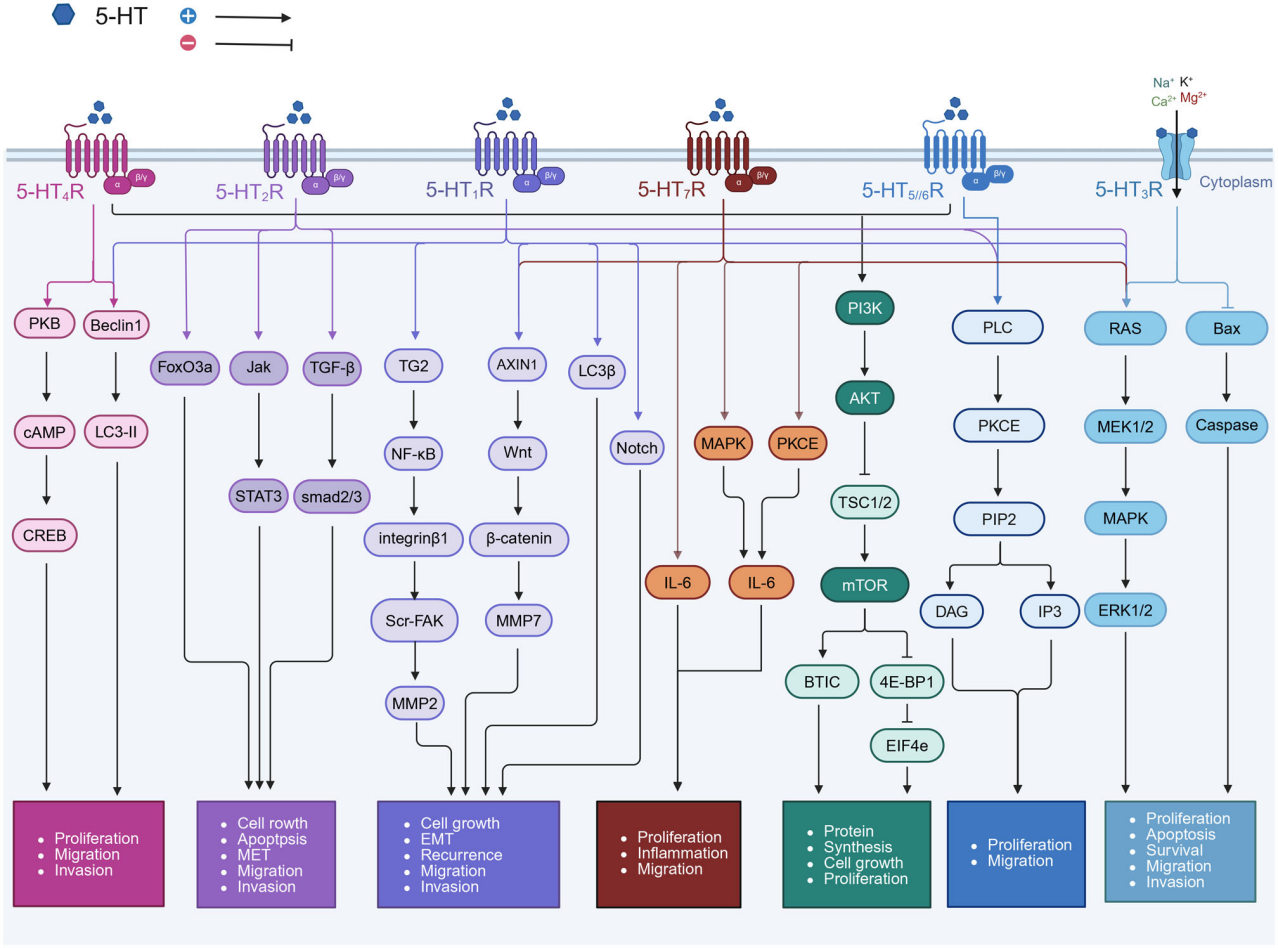
Pathways associated with serotonin (5‐HT) receptors. 5‐HT signals through seven receptors on membrane surfaces. These receptors activate several major interconnected signaling networks, such as those involving PKB/cAMP, PI3K/Akt, Ras/MEK1/2/MAPK, and PLC/PKCE, that trigger cell proliferation, migration, and invasion.

The classification of 5‐HTRs is complex. To date, 14 subtypes of 5‐HTRs have been identified in mammals, and these receptors are grouped into seven families: 5‐HT_1_R to 5‐HT_7_R [11, 72]. By activating different 5‐HTR subtypes, 5‐HT exerts diverse pharmacological effects (Figure 2).

In the CNS, research on 5‐HT has focused primarily on 5‐HT_1_Rs, 5‐HT_2_Rs, 5‐HT_3_Rs, and 5‐HT_6_Rs [15, 73]. 5‐HT_1B_R is predominantly distributed in the striatum and, to a lesser extent, in the basal ganglia, hypothalamus, substantia nigra, pituitary gland, and neocortex [74]. As a presynaptic autoreceptor, 5‐HT_1B_R plays a key role in regulating the synthesis and release of 5‐HT. 5‐HT_1D_R is abundantly expressed in the coronary arteries, cerebral arteries, and ganglia, where it mainly mediates vasodilation [74]. 5‐HT_1E_R, which has a high affinity exclusively for 5‐HT, is diffusely distributed in the cerebral cortex and may be involved in various mental activities [75]. 5‐HT_1F_R exhibits strong binding affinity for tritiated 5‐HT, and it is located primarily in layer V pyramidal neurons of the PFC; in the periphery, it is distributed mainly in the uterus and mesentery [76]. 5‐HT_2A_R is widely expressed in the CA1, CA2, and CA3 regions of the hippocampus, and it is regulated by exogenous 5‐HT neurotransmission [77]. 5‐HT_2B_R is expressed in multiple regions of the spinal cord and brain [77]. 5‐HT_6_R, which is coupled to excitatory guanine nucleotide‐binding proteins (G proteins), activates adenylate cyclase (AC) to initiate intracellular signaling cascades [78, 79].

The role of 5‐HT in peripheral tissues has also received considerable attention. Studies have shown that 5‐HT can promote liver regeneration, 5‐HT_2A_R plays a key role in signal transduction during this process [80]. 5‐HT_2B_R is highly expressed in a wide range of peripheral tissues, including the heart, skeletal muscle, ovary, liver, kidney, lungs, pancreas, trachea, spleen, prostate, and salivary glands [81, 82]. 5‐HT_3_R is expressed at high levels in enteric neurons, particularly in intestinal afferent neurons containing substance P [83]. In the human colon, 5‐HT_3_Rs are most densely distributed in the myenteric plexus and less densely distributed in the muscular and mucosal layers [83]. In the GI tract of rats, 5‐HT_3_Rs are located primarily in the neurons of the myenteric and submucosal plexuses, especially near the serosal membrane of neurons and circular and longitudinal muscle fibers; additionally, in the nerve fibers of the mucosa and submucosa, only low levels of 5‐HT_3_RS expression are found in the interstitial cells of Cajal and enterochromaffin (EC) cells [84]. 5‐HT_4_R is expressed in various GI cell types, including EC cells, smooth muscle cells, secretory cells, and neurons [85]. However, studies on the genetic and functional roles of 5‐HT_5_R in the nervous and digestive systems remain limited. Finally, 5‐HT_7_R is present in enteric neurons and smooth muscle cells [76, 86].

Notably, studies have revealed differential 5‐HT_2B_R expression among patients with subtypes of IBS, suggesting that distinct IBS subtypes may have different underlying pathophysiological mechanisms [87]. Furthermore, 5‐HT and its receptors have been identified in hepatocellular carcinoma (HCC) cells. In these cells, 5‐HT and its receptors are predominantly localized in the cytoplasm, with strong positive expression near the cell membrane, indicating a possible autocrine mechanism of 5‐HT function in HCC [88, 89]. In particular, HCC cells expressing 5‐HT and its receptors demonstrate high proliferative potential [88, 89].

In summary, the distribution of 5‐HTRs varies significantly across different tissues and spatial dimensions. A thorough analysis of their spatial distribution patterns in various tissues and cell types will contribute to a more comprehensive understanding of the physiological functions of 5‐HTR and the signaling mechanisms they mediate.

### 3.2 5‐HTR‐Associated Signal Transduction Pathways

Although 5‐HTRs are differentially distributed across various tissues and organs, the signaling pathways they mediate exhibit a certain degree of consistency. With the exception of 5‐HT_3_Rs, all 5‐HTRs are coupled to G proteins, and thus, they activate second messenger systems and regulate cellular functions through the phosphorylation or dephosphorylation of intracellular proteins [90, 91]. These receptors primarily regulate two classical intracellular second messenger pathways. Moreover, an increasing number of studies have suggested that these receptor subtypes can also activate various other intracellular signaling cascades, indicating that they play more complex roles in the modulation of cellular functions.

#### 3.2.1 5‐HT_2_R

The 5‐HT_2_R family consists of three members: 5‐HT_2A_R, 5‐HT_2B_R, and 5‐HT_2C_R [92]. These receptors are consistently coupled to the phospholipase C (PLC)‐β second messenger pathway in both native tissues and heterologous cells [93]. However, 5‐HT_2_R can also be coupled to other second messenger pathways in a cell‐specific manner [92]. Although 5‐HT_2_Rs are similar in terms of structure, pharmacology, and signaling pathways, there are some differences in their signaling properties [93].

5‐HT_2_R activates PLC‐β in various tissues and cells, leading to the accumulation of inositol phosphates and an increase in intracellular Ca^2+^, which subsequently activates l‐type Ca^2+^ channels and stimulates protein kinases C (PKC) [94]. Additionally, 5‐HT_2A/2C_R can also activate other phospholipases, such as PLA2 and PLD [95, 96]. Moreover, in CHO and 1C11 cells, 5‐HT_2A/2C_R promotes the release of arachidonic acid (AA) via the activation of PLA2 [97], whereas in rat glomerular mesangial cells, 5‐HT_2A/2C_R is coupled to PLD [98], although there is little evidence suggesting that 5‐HT_2B_R can perform the same function. For 5‐HT_2C_R, compared with 5‐HT, some agonists preferentially activate PLA2 or PLC in terms of relative efficacy. 5‐HT_2A/2C_R generally does not regulate cAMP formation in most cells or tissues, but it can either stimulate or reduce cAMP accumulation in specific cell types [99]. In A1 cells, cAMP production is amplified via PKC‐α/δ and Ca^2+^/calmodulin (CaM) [100]. In rat mesangial cells, 5‐HT_2A/2C_R can inhibit intracellular cAMP accumulation and adenylate cyclase (AC) activity in washed membranes, and this effect occurs independently of PLC, Ca^2+^, and PKC [101]. 5‐HT_2_R activates extracellular regulated protein kinases (ERK) in contractile cells, such as vascular smooth muscle cells and mesangial cells [102, 103]. In vascular smooth muscle cells, ERK activation involves a complex mechanism that requires input from PLC, L‐type Ca^2+^ channels, and mitogen‐activated protein kinase (MEK) 1 [104]. In mesangial cells, this pathway also involves PKC stimulation, NADPH oxidase‐like enzyme activation, and ROS (H_2_O_2_ and/or superoxide) production [105, 106, 107]. In mesangial cells, ERK activation via 5‐HT_2A/2C_R depends on the production of ROS by nicotinamide adenine dinucleotide phosphate (NADPH) oxidase, which serves as a critical upstream step. Furthermore, 5‐HT_2A/2C_R is coupled to the Jak/STAT signaling pathway, promoting the phosphorylation of janus kinase (Jak) 2 and signal transducers and activators of transcription (STAT) 3, inducing the nuclear translocation of STAT3, and regulating the expression of genes that are related to myogenic differentiation [108, 109]. 5‐HT_2_R can increase intracellular Ca^2+^ levels either by releasing Ca^2+^ from internal stores or by activating both voltage‐dependent and voltage‐independent Ca^2+^ channels [110]. This effect is associated with Ca^2+^‐activated K^+^ channels and Ca^2+^‐activated Cl^−^ channels in the cell [111]. 5‐HT_2A/2C_R can both promote NO release and inhibit cytokine‐induced inducible nitric oxide synthase (iNOS) expression [112]. Additionally, 5‐HT_2A/2C_R can regulate various transport processes, such as the activation of the Na^+^/H^+^ exchanger in mesangial cells and the Na^+^/K^+^‐ATPase in airway smooth muscle cells. In mouse LMTK^−^ fibroblasts stably expressing 5‐HT_2B_R, rapid activation of the Ras and ERK1/2 MAPK pathways, which involves both the Gαq and Gβγ subunits, has been observed [113, 114]. This signaling axis not only promotes cell proliferation but also leads to tumor formation in nude mice, indicating that tumorigenic potential of 5‐HT_2B_R is primarily mediated by the Ras‐ERK pathway [114]. 5‐HT_2B_R can also drive cell cycle progression via an ERK‐dependent mechanism, upregulating and activating the cyclin D1/cyclin dependent kinase (cdk) 4 and cyclin E/cdk2 complexes, resulting in hyperphosphorylation of the Rb protein [115]. The activation of cyclin D1 depends on the involvement of platelet‐derived growth factor (PDGF) receptor kinase. Moreover, activation of the PDGF receptor, ERK, and cyclin D1/E as well as cell proliferation rely on the function of the nonreceptor tyrosine kinase Src [116].

#### 3.2.2 5‐HT_3_R

To date, five different 5‐HT_3_R subunits have been identified, and among these subunits, the 5‐HT_3A_R and 5‐HT_3B_R subunits are the mature components of the 5‐HTR [117]. Notably, only 5‐HT_3A_R subunits can form functional isoforms, and at least one 5‐HT_3A_R subunit is present in all isomeric receptors. Many researchers refer collectively to various subtypes of 5‐HT_3_R, and studies have also indicated that the selective blockade of 5‐HT_3A_R has the same effect as the complete blockade of 5‐HT_3_R activity [118, 119]. 5‐HT_3_R antagonists reportedly increase cytoplasmic Ca^2+^ levels and ERK1/2 phosphorylation and inhibit microtubule formation [120, 121, 122]. 5‐HT_3A_R promotes the proliferation of lung adenocarcinoma cells by increasing the phosphorylation of ERK1/2 [123].

#### 3.2.3 5‐HT_1_R

The 5‐HT_1_R family consists of five members: 5‐HT_1A_R, 5‐HT_1B_R, 5‐HT_1D_R, 5‐HT_1E_R, and 5‐HT_1F_R [124]. 5‐HT_1_R primarily interact with Gi/o proteins, thereby inhibiting AC activity and regulating various other signaling pathways and effector molecules [125].

5‐HT_1A_R can inhibit AC in the human dorsal raphe nucleus (DRN), but it is ineffective in rats [126]. Under specific conditions, 5‐HT_1A_R and 5‐HT_1B_R can cooperate with the βγ subunit to activate AC in order to promote cAMP expression, whereas 5‐HT_1D_R can stimulate mild levels of cAMP accumulation [126, 127]. This effect is specific to particular cell types: 5‐HT_1_A is effective in HeLa cells [128], whereas 5‐HT_1B_R and 5‐HT_1D_R can increase Ca^2+^ levels in specific cell types, thus contributing to K^+^ channel regulation [129]. 5‐HT_1A_R may also activate AA metabolism via PC‐PLC and PLA2, indirectly regulating nuclear factor kappa‐B (NF‐κB) and ERK signaling [127]. 5‐HT_1_R can activate the ERK signaling pathway, which depends on Gβγ subunits, PC‐PLC, phosphatidylinositol 3‐kinase (PI3K), endocytosis, and ROS [129, 130]. 5‐HT_1B_R activates ERK, protein kinase (Akt), and p70 S6 kinase more efficiently than 5‐HT_1A_R [131]. In 5‐HT_1D_R‐expressing systems, DNA synthesis can also be stimulated, and the proliferative effect is associated with the ERK‐ and PI3K‐related pathways [132]. In CHO cells, 5‐HT_1A_R can induce the production of H_2_O_2_ and superoxide anions, which are critical for ERK activation [133]. Additionally, 5‐HT_1A_R and 5‐HT_1B_R can regulate NO production [134]. The effect of 5‐HT_1A_R on NO varies across systems; it may either promote or inhibit NO generation [134], whereas 5‐HT_1B_R promotes endothelial nitric oxide synthase (eNOS) activity in vascular endothelial cells via a Ca^2+^‐dependent mechanism [135, 136]. Moreover, 5‐HT_1_Rs modulate various ion channels. 5‐HT_1A_R is a classic regulator of G‐protein inwardly rectifying K^+^ (GIRK) channels, and it promotes their opening via Gβγ subunits [137]. 5‐HT_1A_R can also inhibit N‐type and P/Q‐type Ca^2+^ channels in a Goα protein‐dependent manner [138]. 5‐HT_1B_R and 5‐HT_1D_R can activate Ca^2+^‐dependent K^+^ channels, which participate in postsynaptic modulation and negative feedback mechanisms [139, 140]. 5‐HT_1A_R can regulate various transport mechanisms, including Na^+^/K^+^‐ATPase, Na^+^/H^+^ exchange, and Na^+^‐dependent phosphate uptake, via the Ca^2+^ and PKC pathways, suggesting that 5‐HT_1A_R plays important roles in cell volume regulation and energy metabolism [141]. 5‐HT_1A_R promotes the proliferation of T cells, small‐cell lung cancer cells, and carcinoid cells in an ERK signaling‐dependent manner [127]. 5‐HT_1B_R enhances the proliferation of aortic endothelial cells and neuroblastoma cells, whereas 5‐HT_1D_R stimulates DNA synthesis in lung cancer cells [142]. Presynaptic 5‐HT_1A_R and 5‐HT_1B_R receptors have been shown to negatively regulate 5‐HT release [127]. Moreover, 5‐HT_1F_R participates in regulating 5‐HT release and may play a physiological role in antimigraine effects [143]. The pharmacological function of 5‐HT_1E_R remains unclear because of the lack of selective ligands.

#### 3.2.4 5‐HT_4_R

5‐HT_4_R primarily exerts its effects through the activation of AC [144]. The major functional roles of this receptor include prokinetic effects in the GI tract, as well as positive inotropic, chronotropic, and lusitropic effects in the atria but not in the ventricles [145].

5‐HT_4_R typically activates AC in both endogenous tissues and heterologously expressed cells. In the presence of 5‐HT, 5‐HT_4_R has similar abilities to stimulate AC activity [144]. Since the primary function of 5‐HT_4_R is to increase cAMP levels, most of the downstream effects of 5‐HT_4_R are mediated by PKA activation [146, 147]. Additionally, 5‐HT_4_Rs can modulate various ion channels. For example, they regulate Ca^2+^ channels by increasing the intracellular cAMP level and activating PKA [148]. In addition, 5‐HT_4_R enhances the If pacemaker current in atrial myocytes, stimulates Cl^−^ currents in the human jejunal mucosa and rat distal colon, and activates Na^+^ currents in type II dorsal root ganglion cells via a diffusible second messenger pathway that is independent of cAMP, indicating that the receptor can also modulate channel function through the noncAMP/PKA pathway [149, 150]. However, 5‐HT_4_R does not universally activate ion channels. For example, 5‐HT_4_R inhibits Ca^2+^‐activated K^+^ currents, and this effect is also mediated through increased cAMP and PKA activation [151]. Furthermore, the receptor inhibits delayed rectifier K^+^ currents and suppresses voltage‐activated K^+^ channels in colliculi neurons via the cAMP/PKA pathway [152]. In addition, 5‐HT_4_Rs facilitate striatal dopamine (DA) release, acetylcholine (ACh) release from the frontal cortex, and 5‐HT release in the hippocampus [153, 154].

Although the four 5‐HT_4_R variants have similar abilities to stimulate AC, some functional differences between the isoforms have been observed. For example, compared with longer variants such as 5‐HT_4A_R and 5‐HT_4B_R, the 5‐HT_4E_R and 5‐HT_4F_R isoforms can significantly increase AC activity even in the absence of agonists [155, 156]. One proposed explanation is that the C‐terminal region may be involved in rapid or constitutive desensitization, and removal of this region could increase basal activity by reducing desensitization. Recent findings about the B2 bradykinin receptor support this mechanism [157]. Additionally, 5‐HT_4B_R splice variants differ in their inclusion of PDZ interaction domains, which may further influence their signaling properties, thus increasing the complexity of this regulatory mechanism [144].

#### 3.2.5 5‐HT_5_R and 5‐HT_6_R

Previous research on 5‐HT_5_R has been relatively limited. Studies have identified two subtypes within the 5‐HT_5_R family: 5‐HT_5A_R and 5‐HT_5B_R [158]. Both subtypes have been cloned from rats and mice; however, only the 5‐HT_5A_R has been successfully cloned from humans [158]. 5‐HT_5A_R is expressed primarily in several brain regions, including the cerebral cortex, hippocampus, habenula, olfactory bulb, and granule layer of the cerebellum, whereas no 5‐HT_5A_R expression has been detected in peripheral tissues [159, 160]. To date, there is no conclusive evidence that 5‐HT_5A_Rs are directly linked to specific physiological effects or signal transduction pathways in mammalian cells [161]. Recent studies have indicated that human 5‐HT_5A_R may be coupled to GIRK channels [162]. In addition, 5‐HT_5A_R may inhibit cAMP accumulation [3]. In addition to its effects on cAMP levels, the receptor has been implicated in other signaling pathways and the regulation of secondary messengers [163]. Treatment of cells with pertussis toxin abolishes the receptor's ability to inhibit adenosine diphosphate (ADP)‐ribosyl cyclase activity, suggesting that the receptor may signal through Gi/Go proteins. Furthermore, 5‐HT_5A_R has been shown to regulate intracellular Ca^2+^ mobilization via inositol triphosphate (IP3)‐sensitive calcium stores [164]. Nevertheless, the precise G protein subtypes, downstream signaling pathways, and physiological roles of 5‐HT_5_R have yet to be fully elucidated and warrant further investigation.

5‐HT_6_Rs are widely expressed in various brain regions, and they are predominantly distributed in the caudate nucleus, olfactory tubercle, striatum, hippocampus, and nucleus accumbens [165, 166]. Studies have shown that this receptor primarily regulates cholinergic neurotransmission in the CNS, suggesting its potential for use as a therapeutic target for learning and memory disorders [167]. Research has demonstrated that 5‐HT_6_Rs can stimulate AC activity in cultured striatal neurons and pig caudate nucleus membranes [168].

#### 3.2.6 5‐HT_7_R

5‐HT_7_R is highly expressed in the CNS, particularly in the hippocampus, hypothalamus, and neocortex [169, 170]. Owing to its expression in the suprachiasmatic nucleus, the receptor may be involved in the regulation of circadian rhythms [171]. 5‐HT_7_R is also expressed in glial cells, the spleen, vascular smooth muscle, and the intestine [172].

5‐HT_7_R can activate AC, and most of its functions are likely mediated via its coupling with the G protein [173]. 5‐HT_7_R can stimulate several AC isoforms, including the Gs‐sensitive AC5 isoform and the calcium‐sensitive AC1 and AC8 isoforms [173]. The activation of 5‐HT_7_R leads to an increase in the intracellular Ca^2+^ concentration, which is consistent with the calcium sensitivity of AC1 and AC8. However, this increase in Ca^2+^ occurs independently of PKC, phosphoinositide signaling, or Gi proteins, suggesting the involvement of a nonclassical signaling pathway [174]. Furthermore, endogenously expressed 5‐HT_7_R can activate ERK1 and ERK2 in the MAPK pathway, and this activation is insensitive to pertussis toxin, indicating that it likely does not involve Gi/o proteins [175]. 5‐HT_7_R also contributes to vasorelaxation, potentially through mechanisms involving the release of NO or the cAMP‐mediated inhibition of myosin light chain kinase, leading to smooth muscle relaxation [170]. In the nervous system, 5‐HT_7_R is thought to inhibit calcium spike‐induced slow afterhyperpolarization in CA3 hippocampal neurons [171]. However, other studies have shown that the receptor may actually promote afterdepolarization [176]. Research in neurons of the anterodorsal thalamic nucleus has shown that 5‐HT_7_R‐mediated modulation of afterhyperpolarization is driven by cAMP and a hyperpolarization‐activated nonselective cation current [177]. This process occurs independently of PKA or changes in intracellular Ca^2+^ levels, suggesting that a novel signal transduction mechanism may be involved [178].

### 3.3 5‐HTR Crosstalk and Integration

Recent studies have revealed that different 5‐HTR subtypes do not function in isolation but instead engage in functional “crosstalk” through various mechanisms [179]. This crosstalk can manifest as mutual regulation of signaling pathways, changes in ligand‐binding affinities, or even the physical formation of heterodimers or higher‐order receptor complexes [180]. These receptor–receptor interactions contribute to the high complexity and plasticity of the 5‐HT signaling network, playing a critical role in the fine‐tuning of neuronal functions.

For example, 5‐HT_1A_R and 5‐HT_7_R can undergo functional crosstalk or form heterodimers in specific brain regions, such as the hippocampus. The binding affinity of these compounds follows the order 5‐HT_7_R–5‐HT_7_R > 5‐HT_7_R–5‐HT_1A_R > 5‐HT_1A_R–5‐HT_1A_R [181]. Functionally, the activation of 5‐HT_7_R inhibits 5‐HT_1A_R‐mediated Gi signaling, significantly reduces GIRK channel activity, and promotes 5‐HT_1A_R internalization via the β‐arrestin/MAPK pathway [182]. 5‐HT_2A_R participates in crosstalk with several glutamate and DA receptors, such as metabotropic glutamate receptor (mGluR) 2 and D2 receptor (D2R) [183]. In these interactions, the activation of one receptor may alter the ligand‐binding affinity and downstream signaling of the other [183]. For example, D2 receptor activation enhances the affinity of 5‐HT_2A_R for 5‐HT but simultaneously inhibits IP3 production, suggesting that Gq signaling is modulated by Gi pathways [184]. The activation of mGluR2 increases the affinity of 5‐HT_2A_R for 5‐HT, whereas the activation of 5‐HT_2A_R decreases the affinity of mGluR2 [179, 183]. Furthermore, 5‐HT_2A_R and presynaptic mGluR2/3 mutually regulate each other through downstream signaling [185]. Although they are colocalized at glutamatergic terminals in the PFC, 5‐HT_2A_R and mGluR2/3 do not form physical complexes but instead functionally interact in an antagonist‐like manner to modulate glutamate exocytosis [186, 187]. Crosstalk also occurs between ATP receptor (P2X4) and 5‐HT_3_R via their intracellular and transmembrane domains [188]. In addition, mGlu4 and 5‐HT_1A_R exhibit functional interactions, wherein mGlu4 activation suppresses 5‐HT_1A_R activity and subsequently reduces cAMP production [189]. A functional heteromer composed of 5‐HT_2C_R and oxytocin receptor (OTR), which functions as a 5‐HT_2C_R antagonist and enhances OT‐mediated hypoactivity in mice, has been identified [190]. Moreover, phosphatase and tensin homolog deleted on chromosome ten (PTEN) physically interacts with the third intracellular loop (3L4F) of 5‐HT_2C_R and thus activates the PI3K/Akt signaling pathway; this highlights additional layers of complexity in 5‐HTR‐mediated signaling [191]. In brainstem raphe nuclei, autoreceptors such as 5‐HT_1_Rs negatively regulate 5‐HT release, whereas heteroreceptors that are located in the cortex influence emotion, cognition, and even sexual behavior. Chronic use of SSRIs can lead to desensitization of autoreceptors, indirectly enhancing heteroreceptor activity, which may explain the delayed onset of antidepressant efficacy. The activation of 5‐HTRs can initiate multiple intracellular signaling cascades, including the cAMP/PKA, PLC/PKC, and MAPK/ERK pathways. These pathways are often shared among different 5‐HTR subtypes, supporting a model of multipoint cooperative regulation [192].

In addition, inhibitory crosstalk between 5‐HT_2B_R and 5‐HT_7_R depends on NOX and PKA activity [193]. Similar inhibitory interactions have been observed among 5‐HT_2A_R, 5‐HT_7_R, A2A receptor, and CD73 in neuroblastoma cells [194]. These findings suggest that the mechanisms of action for atypical antipsychotics and antidepressants may not be limited to individual receptor signaling but rather involve the modulation of receptor heterodimers, such as 5‐HT_2A_R–mGluR2 or 5‐HT_2A_R–D2R.

Overall, a relatively clear understanding of the mechanisms of 5‐HT‐mediated signal transduction has been established, and studies have confirmed that 5‐HTRs play important roles in various tissues and organs. On the basis of known 5‐HT signaling pathways, the development of targeted drugs is providing new approaches and strategies for the treatment of multiple diseases.

### 3.4 Termination of Signaling

While the mechanisms by which 5‐HTRs are activated have been extensively studied, increasing attention has only recently been given to how 5‐HTR signaling is effectively terminated to prevent prolonged activation, which may lead to toxicity or functional dysregulation [195]. Previous research indicated that 5‐HTR signaling termination involves a series of finely tuned regulatory mechanisms, including receptor desensitization, phosphorylation, β‐arrestin recruitment, endocytosis/recycling and degradation, negative feedback loops, and modulation through receptor heteromerization [196, 197].

Receptor desensitization and phosphorylation represent the primary steps in signal termination [198]. When 5‐HT persistently stimulates its receptors, especially G protein‐coupled 5‐HTRs (e.g., 5‐HT_1A_R, 5‐HT_2A_R, and 5‐HT_7_R), the intracellular regions of these receptors are phosphorylated by G protein‐coupled receptor kinases, thereby promoting the recruitment of β‐arrestin, which in turn disrupts G protein coupling and leads to signal cessation [199, 200]. Moreover, β‐arrestin itself functions as a signaling scaffold, mediating noncanonical pathways such as MAPK/ERK signaling, and thus resulting in biased signaling [201].

Receptor endocytosis followed by either degradation or recycling is another crucial mechanism that modulates signal strength [202, 203]. After being phosphorylated and bound to β‐arrestin, a receptor is internalized into clathrin‐coated vesicles and trafficked to early endosomes [204]. Once internalized, receptors may follow one of two fates: degradation via lysosomal pathways, which reduces receptor availability at the membrane and thus increases cellular sensitivity to 5‐HT [196], or recycling back to the membrane to facilitate renewed signaling [203]. For example, 5‐HT_2A_R and 5‐HT_2C_R tend to be routed toward degradation after prolonged stimulation, whereas 5‐HT_1A_R receptors often exhibit high recycling capacity in certain cell types [203, 205, 206].

Negative feedback regulation also plays a critical role in signal termination. In the raphe nuclei, autoreceptors such as 5‐HT_1B_R and 5‐HT_1D_R sense extracellular 5‐HT levels and inhibit further 5‐HT release, thereby preventing overexcitation and potential neurotoxicity or network instability [207]. In recent years, studies on interreceptor crosstalk and heteromerization have expanded our understanding of the mechanisms that regulate signal termination. For example, 5‐HT_7_R and 5‐HT_1A_R can form heterodimers that influence each other's signaling efficiency and internalization rates [181]; additionally, functional coupling between 5‐HT_2A_R and receptors such as mGluR2 or D2R not only modulates downstream pathways but also may affect desensitization, internalization, or overall signaling duration [179, 183]. These receptor complexes do not always require direct physical interactions; functional synergy or antagonism alone can significantly alter receptor activity status, signaling persistence, and termination dynamics.

Importantly, signal termination is not merely a “shut‐off” process but rather lays the foundation for reshaping future responsiveness, including receptor resensitization, downregulation, or signal switching to alternative pathways. In summary, 5‐HTR signaling termination is a multilayered, dynamic, and highly regulated process that involves molecular‐level events (such as phosphorylation and endocytosis) as well as system‐level mechanisms (such as feedback control and receptor‒receptor interactions).

In conclusion, significant progress has been made in the study of 5‐HT‐mediated signaling mechanisms. However, further exploration is needed in areas such as the functional characterization of receptor subtypes, the integration of signaling networks, and the development of personalized therapeutic strategies to better understand their physiological roles and advance innovative treatments for related diseases.

## 4 Currently Available Therapeutics and Limitations in the Targeting of 5‐HT and its Receptors

On the basis of the signaling networks that are mediated by 5‐HT and 5‐HTR, as well as their extensive regulatory roles in both central and peripheral physiological processes, researchers have gradually developed a variety of modulators that target the 5‐HT system [208]. In recent years, research on the effects of 5‐HT modulators on multiple systems, including the nervous system, cardiovascular system, and endocrine system, has made significant progress. Currently, the therapeutic effects of various 5‐HT‐related drugs in disease treatment have been evaluated. Therefore, further exploration of the potential of these drugs for use in clinical application is essential. The therapeutic effects of 5‐HT modulators in cancer treatment are among the currently popular research topics in the field of oncology (Table 1).

**TABLE 1:** Effects of agonists and antagonists of 5‐HT metabolism‐related enzymes and 5‐HTRs.

Molecule	Target	Cancer type	Clinical development for cancer treatment	Mechanism	Effects	Clinical diseases	References
LP‐533401	^‐^Tph1	Breast cancer	In preclinical development	Inhibits the synthesis of 5‐HT by breast tumor cells, thereby reducing the effect of 5‐HT through autocrine or paracrine pathways	Reduces breast cancer stem cell frequency	Functional gastrointestinal disorders	[209]
Telotristat ethyl (TE)	^‐^Tph1	Pancreatic cancer, colorectal cancer, NET, cholangiocarcinoma	In phase II and III clinical trials	(a) Enhances the accumulation and effect of CD8^+^ T cells; (b) decreases 5‐HT‐mediated PD‐L1 expression	Inhibits tumor growth	Carcinoid syndrome	[210, 211, 212, 213]
LX‐1031	^‐^Tph1	Glioma	In phase I clinical trial	Inhibits 5‐HT‐induced activation of the L1CAM/NF‐κB signaling pathway	Inhibits tumor cell proliferation, migration and chemoresistance	Functional gastrointestinal disorders	[214]
p‐CPA	^‐^Tph1	Cholangiocarcinoma	In preclinical development	Inhibits 5‐HT‐induced activation of 5HT_1A_R, 5HT_2A_R, 5HT_2B_R, 5HT_4_R, and 5HT_6_R	Inhibits tumor growth		[215]
Fluoxetine	^‐^SERT	Various cancer types	In preclinical development	(a) Decreases tumor cyclin D3, cyclin E and cyclin B expression; (b) increases the number of circulating CD8^+^ T lymphocytes and promotes IFN‐γ and TNF‐α secretion	(a) Inhibits tumor growth; (b) promotes tumor apoptosis; (c) promotes T cell‐mediated immunity	Depression, anxiety disorder, obsessive–compulsive disorder, bulimia	[216, 217, 218]
Citalopram	^‐^SERT	Colon cancer	In preclinical development	Inhibits TGF‐β signaling in tumors	Inhibits tumor cell proliferation and migration	Depression, anxiety disorders	[219]
Sertraline	^‐^SERT	Breast cancer, lung cancer, lymphoma	In preclinical development	(a) Inhibits autocrine or paracrine pathways; (b) decreases tumor cyclin D3, cyclin E, and cyclin B expression; (c) inhibits AMPK/mTOR pathway signaling in NSCLC cells	(a) Inhibits tumor growth; (b) promotes antitumor immunity	Depression, social phobia, obsessive–compulsive disorder, posttraumatic stress disorder	[209, 220, 221, 222]
Paroxetine	^‐^SERT	Breast cancer	In phase II and III clinical trials	Suppresses autocrine or paracrine pathways	Reduces breast cancer stem cell frequency	Depression, anxiety disorders, obsessive–compulsive disorder, menopausal mood disorders	[209]
Clorgyline	^‐^MAO‐A	Various cancer types	In preclinical development	(a) Reduces the expression of oncogenes FOS, JUN, NF‐κB, and Myc, as well as cell cycle regulators CCND1, CCNE1, and CDK4/6; (b) inhibits tumor T cell and TAM auto secretion of 5‐HT signaling to promote antitumor immunity	(a) Inhibits tumor cell proliferation, metastasis, and invasion; (b) decreases tumor microvessel density; (c) increases immune cell infiltration		[223, 224, 225, 226, 227, 228, 229]
Phenelzine	^‐^MAO‐A	Prostate cancer, melanoma, colorectal cancer	In phase II and III clinical trial	(a) Suppresses Enz/ARv7/hair‐A signaling in tumor cells; (b) inhibits the secretion of 5‐HT by tumor‐infiltrating T cells and TAMs to promote antitumor immunity	(a) Inhibits the growth and increase in tumor in vitro and in vivo; (b) promotes immune cell infiltration		[227, 229, 230]
NAN‐190	^−^5‐HT_1A_R	Various cancer types	In preclinical development	(a) Blocks MAPK and PI3K/Akt signaling pathways; (b) blocks signaling pathways that are involved in protein translation and survival, such as the Akt/mTOR pathway	(a) Inhibits tumor cell proliferation and metastasis; (b) inhibits tumor microvessel density; (c) increases immune cell infiltration		[231, 232]
WAY‐100635	^−^5‐HT_1A_R	Lymphoma	In preclinical development	(a) Inhibits cell proliferation and metabolism through transcription and translation (e.g., Akt, GSK‐3β, cMyc, and p53); (b) regulates mitochondrial activity by reducing mitochondrial membrane potential and decreasing dehydrogenase activity	(a) Inhibits tumor cell proliferation; (b) promotes apoptosis		[233]
SB216641	^−^5‐HT_1B_R	Uterine leiomyoma, liver cancer	In preclinical development	(a) Decreases cyclin D1 and α‐SMA expression and decreases MAPK, ERK, and EF2K pathway activation; (b) induces caspase‐8, caspase‐9, and caspase‐3 activation	(a) Inhibits tumor cell proliferation; (b) promotes apoptosis		[131, 234]
GR127935	^−^5‐HT_1D_R	Colorectal cancer	In preclinical development	Blocks the Axin1/β‐catenin/MMP‐7 signaling pathway	Inhibits tumor metastasis and invasion		[235]
BRL54443	^−^5‐HT_1E_R	Ovarian cancer	In preclinical development	Activates SRC‐mediated downstream signaling pathways and significantly promotes cell proliferation and EMT	Promotes tumor growth and peritoneal spread		[236]
Ketanserin	^−^5‐HT_2A_R	Choriocarcinoma, breast cancer, lung cancer	In preclinical development	(a) Inhibits MEK‐ERK1/2 and Jak2–STAT3 signaling; (b) inhibits Jak1/STAT3/ERK1/2 and AC/PKA signaling	(a) Inhibits tumor cell proliferation and viability; (b) inhibits tumor cell glycolysis and mitochondrial biogenesis	Hypertension, Raynaud's phenomenon	[108, 237]
SB204741	^−^5‐HT_2B_R	Various cancer types	In preclinical development	(a) Downregulates FOXO3a expression in tumor cells; (b) blocks the 5‐HT–5‐HT_2B_R–pERK–Yap axis; (c) blocks 5‐HT induced phosphorylation of ERK1/2 and eNOS; (d) inhibits 5‐HT‐mediated upregulation of STAB1 and SERPINB2 gene expression	(a) Inhibits tumor cell proliferation; (b) promotes macrophage polarization toward the M1 phenotype; (c) reduces tumor microvessel density		[238, 239, 240, 241, 242]
PRX08066	^−^5‐HT_2B_R	Neuroendocrine tumors	In preclinical development	(a) Inhibits many signaling pathways (e.g., WNT, focal adhesion kinase, and Jak/STAT); (b) inhibits ERK1/2 phosphorylation and profibrotic growth factor synthesis and TGFβ1, CTGF, and FGF2 secretion	(a) Inhibits tumor cell proliferation, metastasis and invasion; (b) inhibits MET fibroblast proliferation		[243, 244]
SB215505	^−^5‐HT_2B_R	Prostate cancer	In preclinical development	Regulates the production of IL‐6, IL‐1β, and TNF‐α by fibroblasts	(a) Inhibits tumor cell proliferation, metastasis and invasion; (b) inhibits MET fibroblast proliferation		[245, 246]
Palonosetron	^−^5‐HT_3_R	Lung cancer	CINV/RINV	(a) Blocks ERK pathway‐induced autophagy in tumor cells; (b) inhibits LC3 protein expression	(a) Inhibits cell proliferation and migration; (b) suppresses the formation of tumor colonies		[247]
Ramosetron	^−^5‐HT_3_R	Lung cancer	CINV/RINV	(a) Blocks ERK pathway‐induced autophagy in tumor cells; (b) inhibits LC3 protein expression	(a) Inhibits cell proliferation and migration; (b) suppresses the formation of tumor colonies		[247, 248]
Tropisetron	^−^5‐HT_3_R	Colorectal cancer, melanoma, lung cancer	CINV/RINV	(a) Inhibits NLRP3 inflammasome activation; (b) promotes apoptosis, microtubule depolymerization, ERK activation, and NF‐κB downregulation	(a) Inhibits tumor cell proliferation; (b) alleviates inflammation; (c) promotes apoptosis; (d) promotes microtubule depolymerization	Nausea and vomiting induced by radiotherapy/chemotherapy	[120, 123, 249]
Ondansetron	^−^5‐HT_3_R	Pancreatic cancer	CINV/RINV	Inhibits Ca^2+^ mobilization in tumor cells	Inhibits tumor cell proliferation	Nausea and vomiting induced by radiotherapy/chemotherapy	[249, 250, 251]
RS23597‐190	^−^5‐HT_4_R	Prostate cancer	In preclinical development	Blocks cell cycle progression in the M phase	Inhibits tumor cell proliferation		[245]
Prucalopride	^+^5‐HT_4_R	Glioma	In preclinical development	Induces autophagy via the Akt–mTOR pathway	(a) Inhibits tumor cell proliferation, migration and invasion; (b) promotes apoptosis; (c) promotes cell autophagy	Chronic constipation	[252]
Mosapride	^+^5‐HT_4_R	Various cancer types	In preclinical development	Blocks cell cycle progression	Inhibits angiogenesis	Indigestion, gastroparesis	[253]
SB699551	^−^5‐HT_5A_R	Breast cancer	In preclinical development	Inhibits Gαi/o signaling and PI3K/AKT/mTOR signaling	(a) Inhibits tumor cell proliferation; (b) reduces tumorsphere frequency		[162]
Valerenic Acid	^+^5‐HT_5A_R	Glioblastoma	In preclinical development	(a) Inhibits 5‐HT‐induced angiogenesis and tumor growth through the PI3K/NOX pathway; (b) increases ROS levels in GBM cells; (c) activates the AMPK pathway	(a) Inhibits tumor cell proliferation, migration, and invasion; (b) promotes tumor cell death	Auxiliary treatments for insomnia, anxiety	[254]
SB258719	^−^5‐HT_7_R	Hepatocellular carcinoma	In preclinical development	Inhibits Wnt/β‐catenin signaling	(a) Inhibits tumor cell proliferation; (b) promotes tumor cell autophagy		[255]
SB269970	^−^5‐HT_7_R	Various cancer types	In preclinical development	(a) Inhibits PI3K/Akt phosphorylation; (b) inhibits cAMP/PKA and CREB activity; (c) inhibits PKCɛ‐ and P38‐induced IL‐6 production	(a) Inhibits tumor cell proliferation; (b) promotes tumor apoptosis; (c) promotes the polarization of macrophages toward the M1 phenotype		[242, 256, 257, 258, 259]
LP211	^+^5‐HT_7_R	Non‐small cell lung cancer	In preclinical development	Activates the Akt and P38 signaling pathways	(a) Promotes tumor cell colony formation; (b) promotes tumor cell migration and invasion		[260]

^+^agonist, ^−^antagonist.

### 4.1 Drugs Targeting 5‐HT Metabolic Enzymes

The biosynthesis and metabolism of 5‐HT are crucial for the regulation of its functions in the body [208]. Changes in the activity of metabolic enzymes not only affect the concentration of 5‐HT but also are directly related to the occurrence and progression of various diseases.

#### 4.1.1 Tph Inhibitors Reduce 5‐HT Synthesis

**FIGURE 3:**
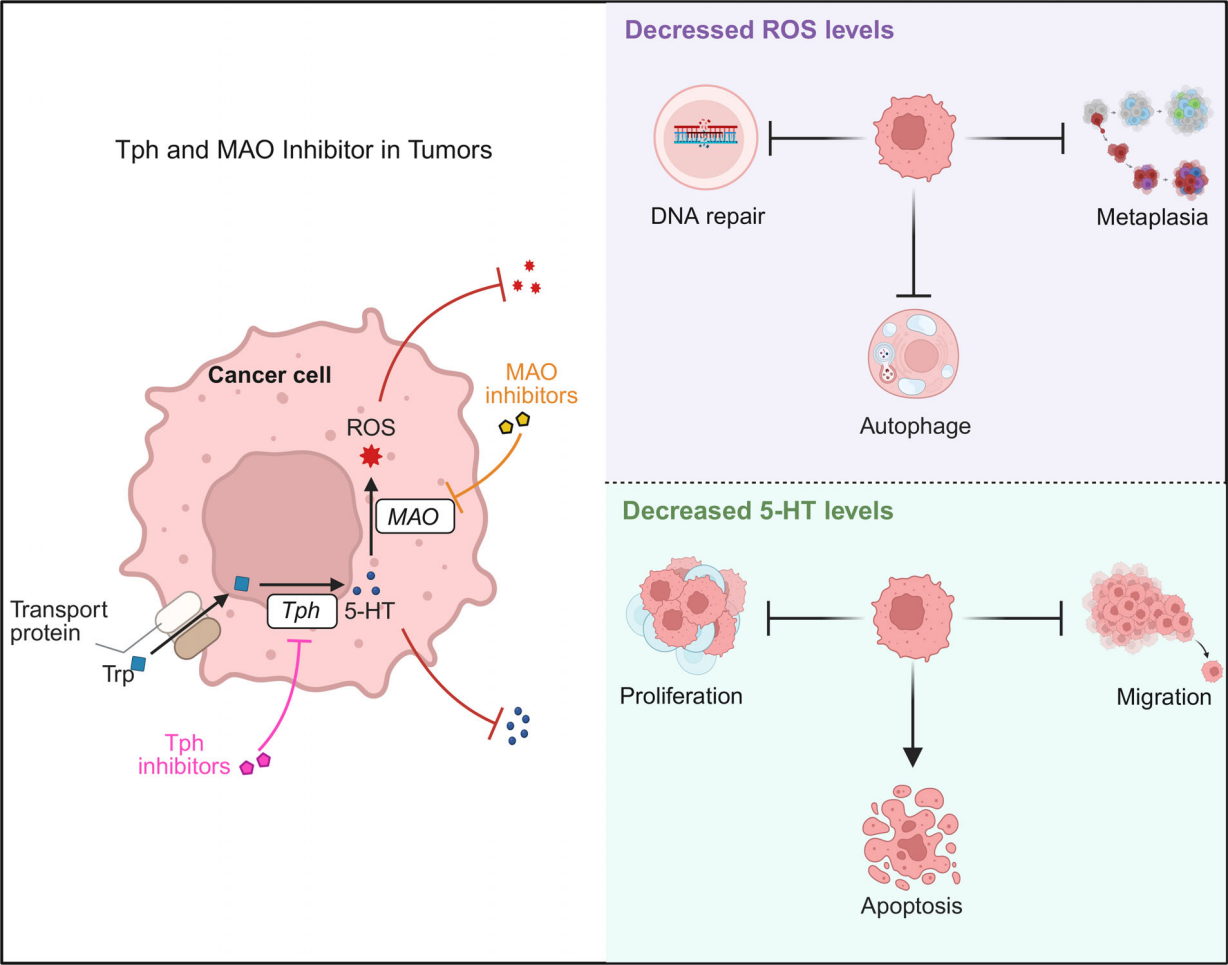
Effects of serotonin (5‐HT) metabolic enzyme antagonists on tumors. Tph1 antagonists block 5‐HT synthesis, resulting in increased tumor cell apoptosis and decreased tumor cell proliferation and migration. MAO‐A antagonists block 5‐HT degradation, resulting in a decrease in intracellular reactive oxygen species (ROS) production and the suppression of tumor cell autophagy and DNA repair.

Tph1, which is the rate‐limiting enzyme in 5‐HT synthesis, has gained attention because of its role in biology [208]. Although 5‐HT production can be increased by Tph1 agonists, no Tph1 agonists are currently available for use in the clinic. Antagonists of Tph1 aim to inhibit 5‐HT synthesis. Telotristat ethyl (TE), which is an oral inhibitor of Tph1, has been approved by the United States Food and Drug Administration (US FDA) to relieve diarrhea symptoms in patients with carcinoid syndrome [210]. Additionally, a Teleace study revealed that cancer patients who were treated with TE experienced a reduction in tumor size [211]. LX‐1031 is a selective peripheral Tph1 inhibitor that was developed for the treatment of diarrhea‐predominant IBS (IBS‐D) [261]. The drug entered phase II clinical trials and demonstrated a certain degree of efficacy and safety because it inhibited intestinal 5‐HT synthesis to alleviate hypermotility symptoms [262]. However, owing to its limited therapeutic efficacy, the development of LX‐1031 has not continued beyond this stage [263]. Moreover, pCPA has been widely used in animal studies to create 5‐HT depletion models for investigations of the roles of 5‐HT in anxiety, depression, aggression, and cognitive function [264]. However, its poor selectivity, irreversible inhibition, and severe side effects have limited its potential for clinical application [265] (Figure 3).

In conclusion, the Tph1 inhibitor TE is currently used in clinical practice to treat carcinoid syndrome. However, the clinical use of other inhibitors has been discontinued because of severe side effects. Therefore, the development of drugs with fewer side effects and greater targeting specificity has become a possible future research direction.

#### 4.1.2 SSRIs Inhibit 5‐HT Reuptake

The use of these medications varies slightly among different populations. For example, fluoxetine, sertraline, fluvoxamine, and escitalopram are approved for use in patients under 25 years of age, whereas citalopram is generally not used in children [266]. Vilazodone, which has partial 5‐HT_1A_R agonistic activity, is approved for the treatment of major depressive disorder (MDD) in adults [267]. Paroxetine is also used to treat menopausal hot flashes and premature ejaculation [268]. Furthermore, SSRIs have applications in migraine, fibromyalgia, IBS, and other nontypical indications [269].

Although SSRIs are effective, their side effects require attention. Common side effects include GI discomfort (such as nausea and diarrhea), headache, insomnia or somnolence, and sexual dysfunction [270]. Citalopram may lead to QT interval prolongation [271, 272]. Owing to its short half‐life, pahroxetine may cause significant discontinuation syndrome, whereas fluoxetine, which has a longer half‐life, has a milder withdrawal effect [273]. Overall, citalopram, fluvoxamine, and paroxetine are associated with slightly higher rates of adverse effects, with citalopram exhibiting the best GI tolerability [267]. In clinical practice, differences among individual patients, drug metabolism characteristics, and potential drug interactions should be considered in the selection of SSRIs. For example, paroxetine may antagonize the metabolism of tamoxifen via CYP2D6, potentially influencing its antitumor effect [274, 275]. Additionally, SSRIs should be used with caution in children, pregnant women, and patients with cardiovascular diseases to ensure drug safety [276].

In summary, SSRIs have been widely used in clinical practice. Recent studies have revealed their potential value in other diseases, such as cancer; thus, further expanding their range of applications will become an important direction for future research.

#### 4.1.3 MAO‐A Inhibitors Block 5‐HT Degradation

MAO‐A is primarily responsible for the breakdown of neurotransmitters, such as 5‐HT, norepinephrine (NE), and DA; therefore, MAO‐A regulates the concentrations of these neurotransmitters in the nervous system and affects mood, stress responses, and various physiological functions [277]. MAO‐A inhibitors (MAOIs) function by inhibiting the activity of MAO‐A, thereby increasing the concentrations of these neurotransmitters and ameliorating mood disorders such as depression and anxiety [278]. Currently, the major MAOIs that have been approved by the US FDA include hydrazine derivatives (isoniazid, iproniazid, and phenelzine), nonhydrazine derivatives, and selective MAOIs (moclobemide, toloxatone, and brofaromine) [279].

MAOIs have a long history of use in treating depression, particularly in patients who do not respond to conventional antidepressants such as SSRIs [278]. Medications such as phenelzine and moclobemide increase the concentrations of 5‐HT, NE, and other neurotransmitters by inhibiting MAO‐A, thereby ameliorating depressive symptoms [280]. MAOIs are also used to treat generalized anxiety disorder and panic disorder [281]. By increasing 5‐HT levels in the brain, MAOIs can effectively alleviate anxiety symptoms. Although MAOIs have potential for use in the treatment of anxiety, they are typically not used as first‐line therapies because of their notable side effects. Some MAOIs have been shown to have therapeutic effects on migraines, particularly in patients with migraines accompanied by depressive symptoms [282]. In patients with depression, MAOIs can ameliorate symptoms such as appetite loss and weight loss [283]. These medications alleviate appetite issues related to depression by increasing 5‐HT levels [283].

Although MAOIs have significant therapeutic effects in the treatment of many conditions, they also have several limitations and side effects [284]. MAOIs can cause severe adverse reactions when combined with foods containing tyramine (such as cheese, cured meats, and soy sauce) and various other medications. These adverse reactions include dizziness, insomnia, muscle weakness, blurred vision, hyperreflexia, respiratory difficulties, and changes in blood counts [285, 286, 287]. Specifically, owing to their shorter half‐life, MAOIs such as paroxetine may result in significant withdrawal symptoms, making discontinuation more challenging [288].

In conclusion, MAOIs have certain clinical application value in the treatment of depression, anxiety, social phobia, and other psychiatric disorders. Although MAOIs can effectively increase neurotransmitter levels and ameliorate mood and behavioral symptoms in patients, owing to the risks of side effects and drug interactions, MAOIs are typically used as second‐line treatment options. Close monitoring and consideration of differences among individual patients are necessary when these medications are used.

### 4.2 Drugs Targeting 5‐HTRs

In recent years, the development of drugs that target 5‐HTRs has made significant progress, especially in the fields of neuro‐psychiatric disease, cardiovascular disease, and cancer treatment, demonstrating great application prospects. This section summarizes the role of drugs that target 5‐HTRs in disease treatment.

#### 4.2.1 5‐HT_2_R Agonists and Antagonists

The 5‐HT_2_R family includes three subtypes, namely, 5‐HT_2A_R, 5‐HT_2B_R, and 5‐HT_2C_R, which play key roles in various physiological and pathological processes. These receptors are regulated by agonists or antagonists and have broad clinical application potential [289].

5‐HT_2A_R agonists directly stimulate 5‐HT_2A_R, thus affecting cognitive, emotional, and perceptual functions. In recent years, research on 5‐HT_2A_R agonists has focused primarily on treating mental disorders such as depression, anxiety, and schizophrenia [290]. Lysergic acid diethylamide and psilocybin are typical 5‐HT_2A_R agonists and have shown potential in the treatment of treatment‐resistant depression, posttraumatic stress disorder, and anxiety, especially in the fields of palliative care and psychological counseling [291, 292]. However, 5‐HT_2A_R agonists are often accompanied by intense hallucinogenic effects; thus, their clinical application requires strict regulation and supervision. 5‐HT_2C_R agonists, particularly lorcaserin, are used as antiobesity drugs on the market [293]. However, owing to its association with risk of cancer development, lorcaserin has been withdrawn from the market [293, 294].

5‐HT_2A_R antagonists have become first‐line drugs for the treatment of schizophrenia, depression, and bipolar disorder. For example, clozapine, olanzapine, and risperidone, which are typical atypical antipsychotics, antagonize 5‐HT_2A_R to ameliorate symptoms such as hallucinations and delusions in psychiatric patients, with fewer side effects than traditional drugs such as chlorpromazine [295, 296, 297]. 5‐HT_2B_R antagonists (e.g., sarpogrelate) are used to treat cardiovascular diseases, especially ischemic symptoms such as ulcers, pain, and cold sensations caused by chronic arterial occlusion [298, 299]. 5‐HT_2C_R antagonists have been used to ameliorate depression and anxiety and have shown potential for appetite control. Some antidepressants, such as azelastine, are both antihistamines and 5‐HT_2C_R antagonists and can be used to alleviate appetite loss and weight loss caused by depression [300].

The 5‐HT_2_R agonist DOI has been shown to stimulate dendritic cell activation [301]. The 5‐HT_2A_R antagonist ketanserin can inhibit the proliferation of JEG‐3 and BeWo choriocarcinoma cells [108]. The 5‐HT_2B_R antagonist SB204741 significantly inhibits the proliferation of Huh7 cells [238]. Additionally, SB204741 reduces microvessel density in the context of lung cancer and melanoma [239]. The 5‐HT_2B_R agonist BW‐723C86 enhances the anti‐inflammatory properties of macrophages [302].

Significant research progress has been made in the use of 5‐HT_2_R agonists and 5‐HT_2_R antagonists in the clinical treatment of mental illnesses, cardiovascular diseases, and obesity. Although these drugs have shown considerable efficacy in treating certain diseases, agonists and antagonists are also associated with side effects and risks; in particular, 5‐HT_2A_R agonists are associated with hallucinogenic effects, and 5‐HT_2B_R agonists are associated with cardiac side effects. With further clinical research and drug safety evaluations, drugs that regulate 5‐HT_2_R still hold vast application potential.

#### 4.2.2 5‐HT_3_R Agonists and Antagonists

5‐HT_3_R is an important neurotransmitter receptor that plays a key role in both the central and peripheral nervous systems. It is particularly involved in regulating nausea, vomiting, GI motility, mood, and other physiological functions [303]. Unlike other G protein‐coupled receptors, 5‐HT_3_R is unique in that it is a ligand‐gated ion channel that directly influences the cellular membrane potential by modulating ion flow [119]. In recent years, research on 5‐HT_3_R antagonists has made significant progress, showing broad potential for the use of these agents in the clinic.

These antagonists function by preventing the activation of 5‐HT_3_Rs, thereby reducing the physiological responses associated with receptor activation, especially under conditions such as nausea, vomiting, and pain [304, 305]. 5‐HT_3_R antagonists, such as ondansetron, granisetron, and dolasetron, are widely used to prevent and treat the nausea and vomiting that are caused by chemotherapy, radiotherapy, and surgery [306]. These agents effectively reduce the incidence of nausea and vomiting by blocking the 5‐HT_3_R‐mediated emetic reflex [307]. Alosetron, which is another 5‐HT_3_R antagonist, alleviates diarrhea and abdominal pain in patients with IBS by inhibiting 5‐HT_3_R in the gut [308]. Although its efficacy is well established, the drug is associated with rare but serious side effects, such as severe constipation and ischemic colitis, and therefore requires strict monitoring during use [308]. Studies have also indicated that 5‐HT_3_R antagonists may offer auxiliary analgesic benefits in patients with conditions such as cancer pain and postoperative pain [309]. However, this indication is still in the preclinical or early clinical research stages. Emerging evidence also suggests that 5‐HT_3_R antagonists may have modulatory effects on anxiety and depressive‐like behaviors. Animal studies and small‐scale clinical trials have shown promising results, although larger clinical studies are needed to confirm these findings [310].

The 5‐HT_3_R agonist 2‐methylserotonin enhances 5‐HT_3_R‐dependent Ca^2+^ influx in immature and mature dendritic cells (DCs) [301]. In addition, the 5‐HT_3_R agonist 2‐methylserotonin promotes T cell activation and proliferation from the S phase to the G2/M phase of the cell cycle [311]. The 5‐HT_3_R antagonists palonosetron and ramosetron effectively inhibit tumor growth and colony formation [247].

In conclusion, 5‐HT_3_R antagonists have become essential therapeutic agents in the management of nausea, vomiting, and IBS, whereas the use of agonists remains largely in the exploratory phase. Both classes of drugs show promising potential for treating various diseases, but further clinical trials and long‐term safety evaluations are necessary to support their broader application.

#### 4.2.3 5‐HT_1_R Agonists and Antagonists

5‐HT_1_R plays a crucial role in regulating neurotransmitter release, and it is involved in processes such as emotional regulation, vasoconstriction, and pain transmission [124]. In recent years, research on 5‐HT_1_R agonists and antagonists has continued to progress, revealing the broad therapeutic potential of these agents in the treatment of psychiatric disorders, neuropathic pain, and cardiovascular diseases [312, 313].

5‐HT_1A_R agonists are the most extensively studied subtype of 5‐HT_1_R‐targeting drugs, and they are widely used to treat psychiatric disorders such as anxiety, depression, and schizophrenia [314]. A typical agent, buspirone, is a partial 5‐HT_1A_R agonist with antidepressant and anxiolytic effects, and it is associated with a low risk of dependence [315]. Newer antidepressants, such as vilazodone and vortioxetine, combine SSRIs with partial 5‐HT_1A_R agonism, and these antidepressants are thought to more effectively alleviate depressive symptoms and cognitive impairment in patients with mood disorders [316, 317, 318]. 5‐HT_1A_R antagonists such as WAY‐100635 are mainly used in experimental research to investigate receptor function, and they have limited clinical application [319]. 5‐HT_1B/1D_R agonists, also known as “triptans,” including sumatriptan, rizatriptan, and zolmitriptan, are the primary medications that are used for migraine treatment [320, 321, 322]. These drugs alleviate headache symptoms by constricting dilated intracranial blood vessels and inhibiting proinflammatory neuropeptide release [323]. Despite their efficacy, triptans can cause vasoconstriction‐related side effects, such as coronary artery spasms, and they should therefore be used cautiously in patients with a history of cardiovascular disease [324]. To mitigate cardiovascular risk, 5‐HT_1F_R agonists such as lasmiditan have been developed as a new generation of antimigraine medications. Unlike triptans, lasmiditan does not induce vasoconstriction, and it has been approved by the US FDA for the treatment of acute migraine attacks, for which it has demonstrated both efficacy and safety [312, 325].

Research on 5‐HT_1_R antagonists is relatively limited, and these antagonists are primarily used to investigate the physiological and pathological roles of 5‐HT1Rs [326]. The 5‐HT_1_R antagonist NAN‐190 induces G2/M phase arrest in HT‐29 cells, whereas the 5‐HT_1B_R antagonist SB224289 causes apoptosis [326]. The 5‐HT_1B_R agonist AnHcl has been shown to stimulate DCs activation [301]. The 5‐HT_1D_R antagonist GR127935 effectively inhibits tumor metastasis [235]. Currently, there are no widely used clinical drugs that function as selective 5‐HT_1_R antagonists.

In summary, 5‐HT_1_R agonists, particularly agonists of the 5‐HT_1A_R and 5‐HT_1B/1D_R subtypes, have significant therapeutic value in the treatment of anxiety, depression, and migraine. The emergence of 5‐HT_1F_R receptor agonists offers a novel option for migraine treatment without cardiovascular risk. Research on other subtypes and a greater understanding of receptor function may further expand the applications of 5‐HT_1_R‐targeted drugs in the treatment of neurological, psychiatric, and vascular disorders.

#### 4.2.4 5‐HT_4_R Agonists and Antagonists

5‐HT_4_Rs are widely distributed in the CNS, GI tract, and cardiovascular system, where they regulate various physiological functions, including GI motility, cognitive function, neurotransmitter release, and cardiac function [327]. In recent years, research on 5‐HT_4_R agonists and antagonists has continued to advance.

5‐HT_4_R agonists are primarily used to enhance GI motility [328]. Drugs such as mosapride, prucalopride, and tegaserod have demonstrated significant efficacy in treating chronic constipation, gastroparesis, and constipation‐predominant IBS (IBS‐C) [329, 330]. These agents activate 5‐HT_4_Rs in the GI tract, promoting ACh release and thereby enhancing intestinal peristalsis. Prucalopride, which is a selective 5‐HT_4_R agonist, has better cardiac safety, whereas tegaserod has been associated with cardiovascular side effects [331]. In addition to GI indications, 5‐HT_4_R agonists have shown promise in ameliorating cognitive impairments and depressive symptoms [332]. Studies have demonstrated that 5‐HT_4_R agonists can enhance learning and memory functions and may have potential antidepressant and antidementia effects [333]. Animal studies also suggest that these drugs may augment the effects of SSRIs and accelerate the onset of antidepressant action [334]. 5‐HT_4_R antagonists are mainly used in experimental models.

In summary, the use of 5‐HT_4_R agonists for the treatment of GI motility disorders has been well established, but their applications in the neuropsychiatric and cardiovascular fields remain at the preclinical or early clinical trial stages. With further understanding of the mechanisms underlying receptor‐mediated functions and the development of new selective drugs, 5‐HT_4_R‐related therapeutics are expected to play broader roles in the treatment of various systemic diseases.

#### 4.2.5 5‐HT_5_R and 5‐HT_6_R Agonists and Antagonists

5‐HT_5_R mainly refers to the 5‐HT_5A_R subtype. Research suggests that 5‐HT_5A_R may play a role in regulating circadian rhythms, cognition, mood, depression, and schizophrenia [335]. Research on agonists of this receptor is still in its early stages, and no drugs have been marketed yet. Research on antagonists of this receptor, however, is relatively more advanced. For example, SB‐699551 has been shown to have antidepressant, cognition‐enhancing, and anxiolytic effects in animal studies [159].

5‐HT_6_Rs are closely related to learning, memory, and cognitive disorders. 5‐HT_6_R antagonists have been used to treat alzheimer’s disease (AD) and cognitive impairments [336]. Idalopirdine has been investigated in multiple clinical trials to assess its ability to ameliorate cognitive function in AD patients, but large‐scale phase III trials have failed to meet primary endpoints [337]. SUVN‐502 and intepirdine were once highly anticipated as adjunctive treatments for AD, but their efficacy was disappointing in later‐stage studies, and their development was hindered [337, 338, 339].

Currently, both 5‐HT_5A_R and 5‐HT_6_R remain relatively underexplored but promising targets, with related drugs still in preclinical research stages.

#### 4.2.6 5‐HT_7_R Agonists and Antagonists

5‐HT_7_Rs are widely involved in regulating various physiological functions, including mood, cognition, sleep, and temperature regulation [171]. In recent years, research on 5‐HT_7_R agonists and antagonists has increased, revealing their potential therapeutic value in various neuropsychiatric disorders [169].

Research on 5‐HT_7_R agonists is relatively limited and has focused mainly on exploring their effects on neurofunction, mood regulation, and cognition [175]. In animal models, 5‐HT_7_R agonists, such as AS19, have shown promising effects in ameliorating cognitive deficits and mood disorders [340]. For example, AS19 promotes M2 phenotypic polarization and cytokine production in macrophages [302]. Additionally, AS19 promotes T cell activation and proliferation [341]. However, despite the significant research potential of agonists, their clinical application remains at an early stage and lacks sufficient clinical validation.

In contrast, research on 5‐HT_7_R antagonists is more developed, and they have been used in the treatment of various diseases. Some 5‐HT_7_R antagonists, such as SB‐269970 and LY‐3154207, have been shown to ameliorate depression, anxiety, and cognitive dysfunction in animal models [342, 343]. In addition, 5‐HT_7_R antagonists have been shown to improve sleep quality, helping alleviate insomnia and other sleep‐related symptoms [344]. Some studies also suggest that 5‐HT_7_R antagonists may play a role in temperature regulation. The 5‐HT_7_R antagonist SB258719 can effectively inhibit the proliferation of liver cancer cells [255]; SB‐258719 also inhibits the growth of xenograft tumors in patients with primary liver cancer [256, 345], modulates cytokine release from DCs, and inhibits ERK signaling in T cells [341].

Overall, both 5‐HT metabolism and 5‐HTR agonists and antagonists have broad potential in the treatment of various diseases. Despite the promising efficacy of these drugs, further clinical trials and drug safety evaluations are needed to advance their clinical application.

## 5 Emerging Therapeutic Opportunities

**FIGURE 4:**
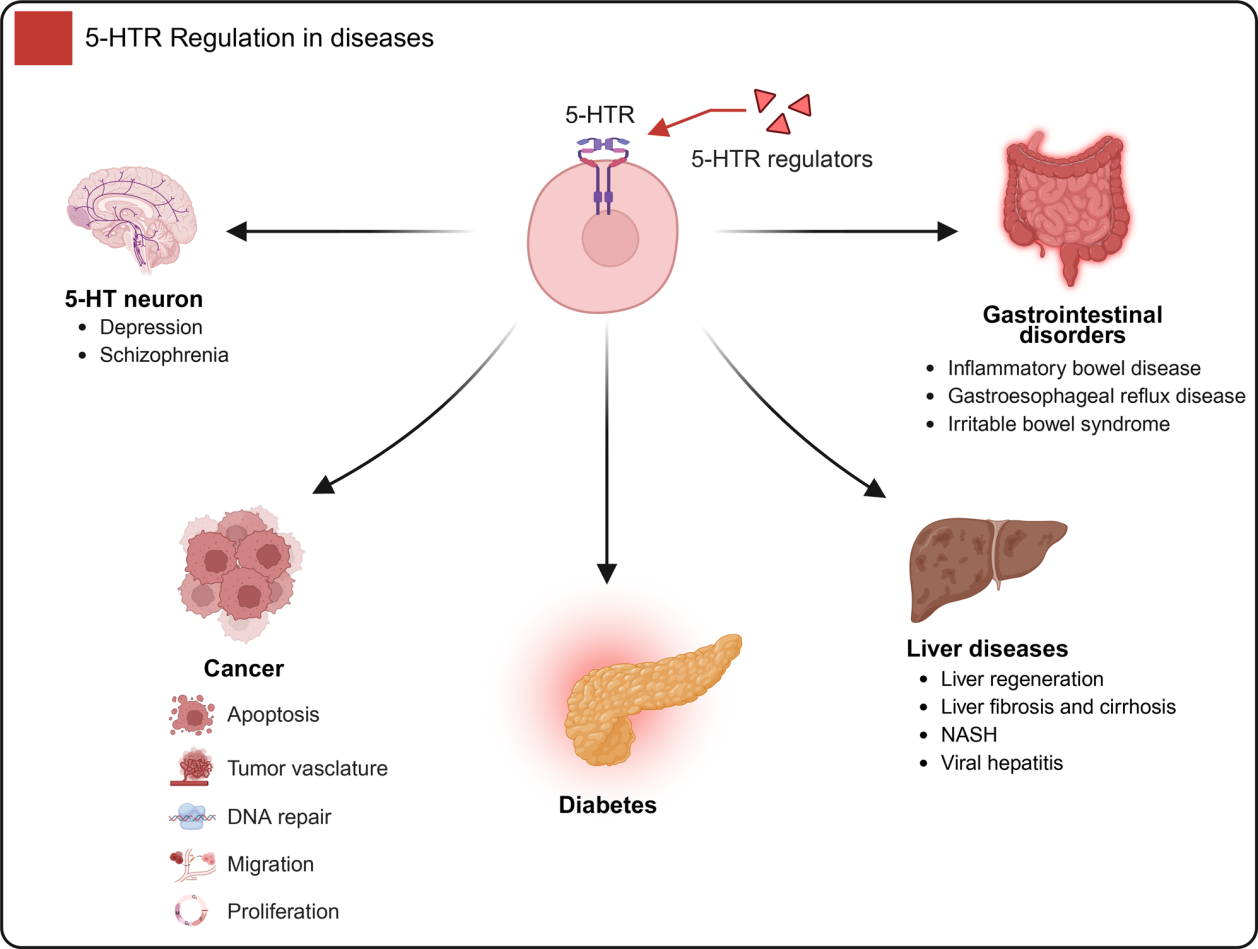
Effects of serotonin (5‐HT) receptor (5‐HTR) regulators on diseases. Blocking 5‐HTRs on the cell surface regulates the progression of a variety of diseases. These include psychiatric diseases, gastrointestinal diseases, liver diseases, diabetes, and tumors. In tumors, blockade of 5‐HTRs inhibits the proliferation and migration of tumor cells and induces their apoptosis.

In recent years, with the successive development of various related drugs, the role of 5‐HT in the treatment of various systemic diseases has attracted increasing attention [290]. Studies have shown that targeting 5‐HT has extensive therapeutic potential, particularly in cancer, immune disorders, neuropsychiatric conditions, metabolic syndromes, and GI diseases [346]. As research continues to elucidate the mechanisms underlying the 5‐HT signaling network under pathological conditions, its potential for use as a target for multitarget intervention platforms is gradually emerging, resulting in the development of a series of novel therapeutic strategies (Figure 4).

### 5.1 The 5‐HT System and Cancer

Recent studies have shown that 5‐HT and 5‐HTRs play significant roles in the occurrence, development, and treatment of cancer [347, 348]. The interactions between the 5‐HT system and the TME, as well as processes such as tumor cell proliferation, migration, and invasion and tumor immune escape, are closely related to this system and have become emerging topics in research in the field of cancer treatment.

#### 5.1.1 Role of 5‐HT Metabolism in Cancer

5‐HT synthesis, transport, and degradation are collectively referred to as 5‐HT metabolism [346]. Tph1 increases the intracellular 5‐HT level, SERT promotes the transport of 5‐HT from the extracellular space to the intracellular space, and MAO‐A reduces the intracellular 5‐HT level by degrading 5‐HT [346]. This review includes a summary of the roles of 5‐HT metabolism‐related enzymes in various types of cancer. Various enzymes that are related to 5‐HT metabolism are abnormally expressed in many tumors and cause 5‐HT metabolic disorders [349]. Therefore, the study of 5‐HT metabolism is very important because 5‐HT metabolism is a potential target for cancer treatment [348].

Recent studies have shown that cancers, such as pancreatic ductal adenocarcinoma, cholangiocarcinoma, breast cancer, glioblastoma, and colorectal cancer, exhibit 5‐HT accumulation, which is accompanied by increased tumor cell proliferation and growth [214, 215, 258, 350, 351, 352]. Clinical and experimental data indicate that increased 5‐HT levels lead to glioma progression and shortened overall survival times. Mechanistically, 5‐HT promotes cell proliferation, invasive migration, and resistance [214]. In 5‐HT‐related pancreatic neuroendocrine tumors, exposure to 5‐HT activates the transforming growth factor (TGF)‐β signaling pathway in pancreatic stellate cells, which is associated with extracellular matrix remodeling; thus, exposure to 5‐HT promotes tumor progression [353]. A reduction in 5‐HT expression can significantly reduce the proliferation of cholangiocarcinoma cells, leading to increased tumor necrosis, enhanced tumor fibrosis, and slowed tumor growth [215]. Additionally, a reduction in 5‐HT can suppress the growth of tumor‐initiating cells and exert a synergistic effect with chemotherapy on the growth of xenografted breast cancer tissue [209]. The use of SSRIs to increase 5‐HT levels before and after the diagnosis of breast cancer has been shown to increase the mortality rate in patients with breast cancer [354]. Clinical data indicate that men with depression who are receiving long‐term SSRI treatment‐induced 5‐HT accumulation and have a significantly increased risk of developing malignant breast cancer [355]. 5‐HT also increases the Ki67 expression and tumor weight of breast cancer and carcinoid tumors [356, 357]. In Tph1‐knockout mice, peripheral 5‐HT levels are significantly decreased, and in Tph1‐knockout mice with colorectal or pancreatic cancer, tumor growth is markedly slower. Furthermore, the expression of the programmed death 1 (PD‐1) ligand 1 (PD‐L1) in tumor cells is reduced, and the accumulation of functional CD8^+^ T cells is increased [212]. In Tph^−/−^ mice, colorectal cancer xenografts exhibit reduced tumor growth and angiogenesis, leading to slower subcutaneous tumor growth, spontaneous tumor necrosis, and tumor hypoxia [350]. Moreover, Tph1 knockout effectively reduces carbon tetrachloride (CCl_4_)‐induced liver tumor formation [358]. 5‐HT enters cells via SERT and activates the Ras homolog family member A (RhoA)/Rho‐associated kinases (ROCKs)/Yes‐associated protein (Yap) signaling pathway, thereby promoting the development of colon cancer [359]. Furthermore, colorectal cancer stem cells synthesize large amounts of 5‐HT, promoting the self‐renewal of cancer stem cells and playing a significant role in the development and metastasis of colorectal cancer [360]. Therefore, inhibiting peripheral 5‐HT synthesis may have therapeutic applications, such as in the prevention of cancer progression [361]. The elevated levels of circulating free 5‐HT induced by SSRI treatment promote the progression of cholangiocarcinoma [362]. 5‐HT promotes the apoptosis of Burkitt lymphoma cells [363]. 5‐HT in neutrophils is activated through histone H3 modification, promoting liver metastasis of neuroendocrine prostate cancer [364]. SSRIs block 5‐HT transport into neutrophils and inhibit 5‐HT‐mediated H3 modification, reversing prostate cancer liver metastasis [365]. 5‐HT also inhibits the neural invasion of prostate cancer cells through semaphorin 3C (SEMA3C)/PlexinA2/neuropilin‐1 (NRP1)/cellular‐mesenchumal epithelial transition factor (cMET) signaling, which reduces the density of tumor‐infiltrating nerve fibers and promotes the growth and migration of mouse xenograft tumors [366]. 5‐HT suppresses the induction of MAO‐A expression; promotes cancer cell proliferation and migration by activating downstream molecules, such as p21, matrix metalloproteinases 2 (MMP2), and vascular endothelial growth factor (VEGF); and promotes epithelial‐to‐mesenchymal transition (EMT) and hypoxia inducible factor‐1 (HIF1) α protein accumulation [367, 368]. Additionally, 5‐HT is highly expressed in classical Hodgkin lymphoma cells. 5‐HT suppresses the induction of MAO‐A expression, inhibits tumor cell proliferation and is efficacious in the majority of patients when combined with adriamycin, bleomycin, vinblastine, or dacarbazine (ABVD) [228]. This regimen presumably sensitizes tumor cells to ABVD treatment. However, approximately 20% of patients experience refractory disease or relapse after initial treatment [228]. Additionally, 5‐HT reduces the ability of induced MAO‐A to affect the TME and hinder tumor immunotherapy. Wang et al. [226] reported that suppressing the 5‐HT‐mediated induction of MAO‐A promotes the polarization of TAMs toward the M2 phenotype via oxidative stress. Furthermore, 5‐HT suppresses the induction of MAO‐A expression, inhibits tumor‐associated macrophage (TAM) reprogramming, induces tumor growth in xenograft tumor models, and reduces synergistic antitumor effects when combined with anti‐PD‐1 therapy [226]. Wang et al. [227] reported that 5‐HT suppresses the induced MAO‐A‐mediated suppression of antitumor T cell immunity by regulating autocrine 5‐HT signaling in tumor‐infiltrating CD8^+^ T cells [227].

Furthermore, some studies have suggested that 5‐HT overproduction can inhibit tumor development [369, 370]. 5‐HT can inhibit the proliferation of colon cancer cells in mice [371]. Additionally, in colon cancer cells, 5‐HT production is induced by dimethylhydrazine, which inhibits microvascular formation in the TME and affects mitochondrial function and energy production in cancer cells [372]. In melanoma, 5‐HT can slow tumor growth by inhibiting the production of interleukin (IL)‐10 and interferon (IFN)‐γ and increasing the production of IL‐1β [216, 373]. In non‐small cell lung cancer, SSRIs limit tumor growth and increase tumor sensitivity to erlotinib by inhibiting the adenosine 5‐monophosphate adenosine 5‘‐monophosphate (AMP)‐activated protein kinase (AMPK)/mammalian target of rapamycin (mTOR) signaling pathway [220]. Additionally, 5‐HT can modulate immunity, reversing the suppression of antitumor immune responses due to chronic stress [374]. 5‐HT reduces the expression of IL‐4I1 and PD‐L1 and decreases the numbers of inhibitory immune cells, MDSCs, Tregs, and CD39^+^/PD‐1^+^ CD8^+^ T cells. Additionally, the SSRI amitriptyline (AMI) enhances the cytotoxic potential of CD8^+^ T cells and exerts a synergistic effect with PD‐1 [375]. Furthermore, research has shown that low 5‐HT expression is associated with poor clinical outcomes in prostate cancer patients [376]. Reduced 5‐HT levels promote prostate tumorigenesis and development [376, 377]. 5‐HT reduces glioma cell proliferation and invasion as well as microvessel density while reducing macrophage infiltration [224, 225].

Thus, these reports suggest that the dysregulation of 5‐HT, which is regulated by 5‐HT metabolic enzymes, is involved in tumor growth and immune regulation in the TME. Therefore, reducing intratumoral 5‐HT levels by blocking Tph1 and SERT or promoting MAO‐A expression may be a target for cancer therapy as well as for the design and development of antineoplastic drugs. However, other studies have shown that the aberrant expression of MAO‐A in many cancers promotes tumor progression by reducing tumor 5‐HT levels, which may be related to the heterogeneity of the tumors studied. Therefore, targeting 5‐HT metabolism to regulate tumor 5‐HT levels has become a potential target for the design and development of cancer therapeutic drugs.

#### 5.1.2 Roles of 5‐HTRs in Cancer

5‐HT‐induced 5‐HTR activation can affect the development, recurrence and metastasis of tumors [347]. This section focuses on the relationships between 5‐HTRs and the occurrence, development, and prognosis of tumors. The expression of 5‐HTRs in various cancer tissues is shown in Table 2. The role of 5‐HTRs in cancer is discussed further below.

**TABLE 2:** Roles of 5‐HTRs in normal and tumor microenvironments.

Receptor	Normal vs. tumor	Tumor type	Mechanism	Response	References
5‐HT_1B/1D/1F_R		Colorectal cancer, pancreatic ductal carcinoma	5‐HT activates 5‐HTRs to initiate Wnt/b‐catenin signaling in order to promote the self‐renewal of colorectal cancer stem cells, integrin/Src/Fak‐mediated signaling, uPAR/MMP‐2 signaling, and zinc finger ZEB1 and Snail protein expression.	Tumor cell proliferation and migration	[360, 378]
5‐HT_1A_R	Upregulated in tumors	Lung cancer, lymphoma	5‐HT activates 5‐HT_1A_R, and then the 5‐HT_1A_R/autophagy/p‐STAT3 axis increases the abundance of Tregs and reduces the Th1/Th2 ratio. )5‐HT activates 5‐HT_1A_R, and then inhibits DNA damage and ROS‐independent caspase activation.	Tumor cell proliferation, tumor recurrence, and tumor metastasis	[233, 379]
5‐HT_1D_R	Upregulated in tumors	Hepatocellular carcinoma	5‐HT_1D_R activated by 5‐HT inhibits PIK3R1 ubiquitin degradation, thereby increasing FoxO6 expression via the PI3K/Akt pathway.	Increased probability of tumor recurrence	[380]
5‐HT_1E_R	Upregulated in tumors	Ovarian cancer	5‐HT_1D_R activated by 5‐HT inhibits the activation of factors related to SRC	Inhibits tumor cell proliferation and EMT	[236]
5‐HT_1B_R	Upregulated in tumors	Uterine leiomyoma, colorectal cancer, lymphoma	5‐HT_1B_R activated by 5‐HT activates the MAPK/ERK pathway, EF‐2 kinase, the cyclin D1 pathway and α‐smooth muscle antigen; inhibits the caspase pathway; and inhibits apoptosis in tumor cells.	Promotes tumor cell proliferation and tumor metastasis	[131, 233, 235, 326]
5‐HT_2B_R	Upregulated in tumors	Colorectal cancer, hepatocellular carcinoma, prostate cancer, uveal melanoma, pancreatic ductal adenocarcinoma, gastric cancer	5‐HT_2B_R activated by 5‐HT activates TGF‐β signaling and promotes ERK phosphorylation, thereby promoting the upregulation of Yap expression. 5‐HT_2B_R activated by 5‐HT activates Jak/STAT and adhesion kinase; 5‐HT_2B_R activated by 5‐HT increases the Warburg effect and PI3K/Akt/mTOR signaling by increasing the protein levels of HIF1 α and Myc. 5‐HT_2B_R activated by 5‐HT activates the FoxO3a pathway in liver cancer cells	Inhibits early CAC initiation but promotes late CAC progression Promotes the proliferation, invasion and metastasis of liver cancer, prostate cancer, melanoma, and pancreatic ductal cancer cells Inhibits tumor apoptosis	[238, 240, 241, 243, 245, 381, 382]
5‐HT_2A/2C_R	Upregulated in tumors	Colorectal cancer, choriocarcinoma, breast cancer	5‐HT_2A/2C_R activated by 5‐HT activates the ERK‐mediated pathways (c‐Jun and Ki67 transcription). 5‐HT_2A/2C_R activated by 5‐HT activates the STAT3 and ERK1/2 signaling pathways through Jak2.	Promotes tumor cell proliferation Promotes choriocarcinoma cell growth and survival	[383, 384]
5‐HT_3_R	Upregulated in tumors	Pancreatic cancer, neuroblastoma, lung cancer, esophageal squamous cell carcinoma, colorectal cancer, melanoma, gastric cancer	5‐HT_3_R activated by 5‐HT inhibits the expression of BAD and Bax and upregulates the expression of Bcl‐2, inhibits cAMP expression, inhibits ERK phosphorylation, and increases intracellular ([Ca^2+^]i) and causes Ca^2+^ influx, which in turn causes CaMKIIa phosphorylation and activation, leading to NLRP3 phosphorylation and inflammasome assembly.	Promotes tumor cell proliferation Promotes metastasis and spread of esophageal squamous cell carcinoma tumor cells	[123, 352, 385, 386, 382, 387, 388, 389]
5‐HT_4_R	Upregulated in tumors	Prostate cancer, adrenal cancer, glioblastoma	5‐HT_4_R activated by 5‐HT activates the Akt/mTOR pathway, thereby inhibiting cleaved caspase 3 and Bax and promoting Bcl‐2, reduces LC3‐II and Beclin 1 expression.	Promotes tumor cell proliferation, migration, and invasion	[245, 252]
5‐HT_5_R	Upregulated in tumors	Breast cancer, glioblastoma, prostate cancer	5‐HT_5_R activated by 5‐HT enhances ROS levels and activates the AMPK pathway. Activates the PI3K/Akt/mTOR axis. 5‐HT_5_R activated by 5‐HT promotes the PKA pathway to increase androgen receptor activation.	Inhibits glioblastoma cell proliferation and migration Promotes the proliferation of breast and prostate cancer cells	[162, 254, 390]
5‐HT_7_R	Upregulated in tumors	Non‐small cell lung cancer, prostate cancer, adrenocortical carcinoma, glioblastoma, liver cancer, breast cancer	5‐HT_7_R activated by 5‐HT activates the P38 MAPK and NF‐κB signaling pathway and activates the cAMP⁄PKA signaling axis, leading to increased phosphorylation of CREB and ERK⁄Akt. Simultaneously, it activates the kinase signaling pathway and the PI3K/Akt signaling axis.	Promotes the proliferation, migration, and spread of non‐small cell lung cancer, prostate cancer, and breast cancer cells	[256, 258, 260, 391]

##### 5.1.2.1 5‐HT_2_R

5‐HT_2_R affects tumors [20]. The 5‐HT_2_R family consists of three subtypes, namely, 5‐HT_2A_R, 5‐HT_2B_R, and 5‐HT_2C_R. 5‐HT_2A_R expression is significantly upregulated in tumor cells, and 5‐HT_2A_R enhances proliferation by altering cell cycle progression [108, 392]. 5‐HT_2A_R activation also promotes skin cancer progression. Furthermore, 5‐HT_2A_R‐overexpressing mice exhibit a significantly increased incidence of skin cancer, and platelet‐activating factor halts skin cancer progression [393]. Peripheral 5‐HT phosphorylates ERK through 5‐HT_2A_R, thereby increasing the Yap/vestigial like family member 4 (VGLL4) ratio [394]. The 5‐HT_2A_R–p‐ERK–Yap axis promotes liver cancer progression [395, 396]. Furthermore, the expression of 5‐HT_2B_R has been detected in breast cancer and is closely associated with the activation of estrogen receptor‐α [397]. Studies have shown that 5‐HT, through 5‐HT_2B_R, inhibits autophagy and downregulates the expression and phosphorylation of FoxO3 in HCC cells, thereby promoting the proliferation and growth of these cells [238, 398]. In prostate cancer, 5‐HT_2B_R expression is upregulated in high‐grade pathological subtypes [245]. Compared with normal pancreatic tissue, 5‐HT_2B_R is overexpressed in pancreatic cancer tissues and is closely associated with poor clinical prognosis [241, 399]. Previous studies have confirmed the oncogenic role of 5‐HT_2B_R in pancreatic cancer [241]. In colorectal cancer, the expression of 5‐HT_2B_R is reduced in early colorectal cancer and promotes tumor growth [381]. Knocking out 5‐HT_2B_R in late‐stage colorectal cancer inhibits tumor development via tumor cell proliferation and migration [381]. In uveal melanoma, the transcription levels of 5‐HT_2B_R are significantly elevated, leading to increased intracellular expression of the 5‐HT_2B_R protein [400]. 5‐HT_2B_R promotes tumor cell proliferation and migration through the activation of multiple signaling pathways, such as focal adhesion kinase and Jak [243]. Moreover, 5‐HT_2B_R can promote the nuclear translocation of cAMP response element‐binding protein 1 (CREB1), which further enhances the transcription of zinc finger e‐box binding homoeobox 1 (ZEB1), thereby increasing the migration and promoting the EMT process of colorectal cancer cells [401].

Studies have shown that 5‐HT can be involved in tumor progression via signaling through various subtypes of 5‐HT2Rs [402]. Both 5‐HT_2A_R and 5‐HT_2C_R are also expressed in breast cancer cells and tissue samples [403, 404]. Research has shown that 5‐HT can increase oxidative phosphorylation and glycolytic activity in MCF‐7 cells via the 5‐HT_2A/2C_R signaling pathway, thereby promoting cell proliferation and inhibiting apoptosis [405]. In addition, this signaling pathway activates the transcriptional coactivator PPAR gamma coactivator 1alpha (PGC‐1α), thus promoting mitochondrial biogenesis in MCF‐7 cells [406, 407]. In contrast to other studies, a study by Muller et al. [408] revealed that human cutaneous melanoma cells exhibit high 5‐HT_2A_R, 5‐HT_2B_R, and 5‐HT_2C_R expression. 5‐HT can enhance the inhibition of melanoma cell proliferation induced by radiation, possibly through the release of 5‐HT by mast cells in response to ionizing radiation [408]. These findings suggest that 5‐HT_2_R plays an important role in tumor progression. Systematic analysis of the regulation of 5‐HT_2_R expression in various cancers thus has substantial implications for the development of effective targeted drug treatments.

##### 5.1.2.2 5‐HT_3_R

Many researchers refer collectively to various subtypes of 5‐HT_3_R, and studies have also indicated that the selective blockade of 5‐HT_3A_R has the same effect as the complete blockade of 5‐HT_3_R activity [118]. Early investigations often combined 5‐HT_3_R antagonists with other chemotherapeutic drugs to ameliorate the vomiting that is caused by anticancer chemotherapy [409]. In recent years, studies have shown that in addition to reducing vomiting, 5‐HT_3_R directly affects tumor progression [410]. Clinical data have shown that the expression of the 5HT_3A_R gene is upregulated in tumor tissue compared with that in tumor margin tissue in patients with breast cancer [411]. The breast cancer cell line MCF‐7 expresses 5‐HTR_3A_R isoforms [403], and 5‐HT enhances the proliferation of MCF‐7 cells via 5‐HT_3A_R signaling. The inhibition of 5‐HT_3A_R with chemical antagonists can reduce the proliferation and induce the apoptosis of MCF‐7 cells [403]. 5‐HT_3_R is also expressed in human colon cancer cells, and activation of this receptor by 5‐HT promotes mitosis while inhibiting apoptosis [412]. Similarly, 5‐HT_3_R antagonists inhibit the proliferation of melanoma cells, promote apoptosis and exert a synergistic effect when combined with paclitaxel. In addition, 5‐HT_3_R antagonists reportedly increase cytoplasmic Ca^2+^ levels and ERK1/2 phosphorylation and inhibit microtubule formation [120]. Lung adenocarcinoma cells also express 5‐HT_3A_R, which promotes their proliferation [123]. In colon cancer, 5‐HT_3A_R is expressed at different levels across several cell lines, including SW1116, HCT116, DLD‐1, LoVo, SW62, and RKO cells, and promotes the proliferation of these cells [413]. Knockdown of 5‐HT_3A_R causes cell cycle arrest and increases the expression levels of proapoptotic proteins, including Bcl2 associated X protein (Bax), that promote apoptosis [385]. Similarly, both colon cancer tissue and colon cancer cells exhibit high levels of 5‐HT_3_R expression, and inhibiting 5‐HT_3_R slows tumor growth [352, 414]. Huang et al. [386] reported that 5‐HT_3A_R promotes the proliferation and migration of esophageal squamous cell carcinoma cells, mainly via the long noncoding RNA linc01305, the stability of which is maintained via its interaction with 5‐HT_3A_R mRNA.

These findings suggest that 5‐HT_3_R may have significant effects on tumors. Considering that it is the only ion channel receptor among 5‐HTRs, it may play a different role than other receptor subtypes do. The exact role of 5‐HT_3_R in tumors still needs to be elucidated through large‐scale studies and clinical trials.

##### 5.1.2.3 5‐HT_1_R

Various 5‐HT_1_R isoforms have been studied in tumors, and their expression has been shown to be correlated with cancer progression and prognosis. However, the distribution of 5‐HT_1_R isoforms in tumors is heterogeneous [415, 416]. 5‐HT_1A_R and 5‐HT_1B_R are upregulated in benign and noninvasive tumors but downregulated in invasive tumors [417]. Multiple ovarian cancer cell lines (ES2, OV90, SW626, CaOV3, 2774, TOV112D, UWB1.298, SKOV3, and HEYA8 cells) also overexpress 5‐HT_1A_R, 5‐HT_1B_R, and 5‐HT_1D_R; moreover, two ovarian cancer cell lines (2774 and CaOV3 cell) also express the 5‐HT_1E_R isoform [417]. In contrast, the expression of 5‐HT_1E_R is markedly reduced in peritoneally disseminated ovarian cancer cells, and this reduction significantly increases tumor cell proliferation via the activation of Src‐mediated downstream signaling pathways [236]. Siddiqui et al. [418] also measured the expression of 5‐HT_1A_R and 5‐HT_1B_R in human bladder cancer tissues and in HT1376 cells. Clinical data also indicate that the 5‐HT_1A_R subtype is highly expressed in lung cancer tissues from patients with depression [379]. Additionally, 5‐HT_1D_R has been implicated in lung cancer cell proliferation [419]. Importantly, 5‐HT_1A_R expression in lung cancer cells is negatively correlated with the activity of cytotoxic T lymphocytes (CTLs) among tumor infiltrating lymphocytes (TILs). Patients with increased intertumoral 5‐HT_1A_R expression exhibit increased Treg infiltration and a decreased Th1/Th2 cell ratio, as well as upregulated PD‐L1 expression in the tumor microenvironment (TME) [379].

Additionally, 5‐HT_1_R has been reported to be associated with liver cancer. 5‐HT levels are elevated in a chemically induced HCC mouse model, and 5‐HT expression levels are correlated with HCC prognosis and progression [420]. In vitro studies have demonstrated that 5‐HT promotes FoxO6 expression via 5‐HT_1B_R and 5‐HT_1D_R, thereby increasing liver cancer cell proliferation [380]. Moreover, 5‐HT_1B_R signaling promotes autophagy and activates Notch signaling by upregulating the expression of light chain 3 (LC3) β and autophagy‐related effector proteins, such as elF4E‐binding protein 1 (4EBP1), autophagy related 3 (ATG3), Beclin1, and s65, thereby inducing liver cancer cell death [421].

In mouse models of colorectal cancer, 5‐HT_1D_R overexpression has been shown to promote tumor cell invasion [235]. Human HT29 colon cancer cells and uterine leiomyoma cells express high levels of 5‐HT_1B_R [131, 326]. Furthermore, the 5‐HT_1B/1D/1F_R isoforms are highly expressed in colorectal cancer stem cells, and the binding of 5‐HT to these receptors promotes self‐renewal, tumor initiation, and migration in these cells [360].

5‐HT_1B_R and 5‐HT_1D_R are more highly expressed in pancreatic cancer cells than in normal pancreatic cells [378]. Overexpression of 5‐HT_1D_R modulates β1 integrin activity, promoting the recruitment of the Src–FAK complex, which enhances cancer cell proliferation and migration. Furthermore, 5‐HT_1C_R is highly expressed in chordomas in transgenic mice and promotes tumor growth [422].

In summary, these findings indicate that 5‐HT_1_R promotes the occurrence and development of tumors. Abnormally high expression levels of 5‐HT_1_R lead to the occurrence and development of various cancers. However, previous research on 5‐HT_1_Rs has focused mainly on tumor cells themselves. The effects of 5‐HT_1_R on immune cells, especially the regulatory role of immune cells in the TME, have not been studied thoroughly and require further exploration.

##### 5.1.2.4 5‐HT_4_R

Compared with the large number of studies on 5‐HT_1, 2, 3_R in cancer, relatively few studies have explored the role of 5‐HT_4_R in tumors [423]. Current evidence indicates that 5‐HT_4_R is upregulated in various tumor types, including cholangiocarcinoma, colon cancer, and breast cancer, where 5‐HT promotes tumor cell growth and proliferation through 5‐HT_4_R‐mediated signaling [412]. In addition, 5‐HT_4_R is upregulated in high‐grade prostate cancer cell lines, such as DU145 and LNCaP [245, 424]. These 5‐HT_4_R isoforms have been shown to promote tumor cell proliferation even under androgen‐deficient conditions [424]. In the ovary, 5‐HT_4_R is expressed in both normal tissues and ovarian malignancies. Notably, 5‐HT_4_R is overexpressed in benign and noninvasive ovarian tumors, whereas its expression is downregulated in more invasive ovarian cancers [417].

In contrast to its tumor‐promoting effects in many cancer types, 5‐HT_4_R activation has antitumor effects on gliomas. Specifically, 5‐HT_4_R agonists inhibit the proliferation, migration, and invasion of glioma cells; promote the apoptosis and autophagy of glioma cells; and significantly increase the expression of Beclin1 and LC3‐II [252].

These findings illustrate the role of 5‐HT_4_R in cancer progression. While its tumor‐promoting effects are evident in several cancers, such as breast, prostate, and colon cancers, 5‐HT_4_R activation may have inhibitory effects on gliomas. Despite these findings, the mechanisms by which 5‐HT_4_R functions in tumor cells have not been fully studied and need to be explored.

##### 5.1.2.5 5‐HT_5_R and 5‐HT_6_R

Although the role of 5‐HT_4_Rs in cancer has been demonstrated in several studies, the roles of 5‐HT_5_Rs and 5‐HT_6_Rs in tumors have rarely been studied. Recent studies have shown that a 5‐HT_5A_R antagonist reduces the frequency of tumor microsphere‐initiating cells among breast cancer cells and synergizes with chemotherapy to inhibit the growth of xenograft tumors by targeting the Gα‐coupling pathway [162]. Additionally, high 5‐HT_5A_R expression is also observed in prostate cancer. 5‐HT_5A_R increases androgen receptor activity by activating PKA signaling, which promotes tumor growth [390]. In contrast, although 5‐HT_5A_R isoforms are expressed in glioma tissues, their levels are lower in glioma cells than in normal brain tissues [254]. Interestingly, the activation of 5‐HT_5A_R with the agonist valine has been shown to inhibit the proliferation and invasion of glioma cells [254].

Furthermore, 5‐HT_6_R is expressed in cholangiocarcinoma cells, and 5‐HT promotes their proliferation through 5‐HT_6_R signaling [215].

These findings suggest that 5‐HT_5A_R and 5‐HT_6_R may have significant effects on tumors. However, the role of 5‐HT_5_R and 5‐HT_6_R in tumors has not yet been explored in depth. Thus, the mechanisms underlying these processes in tumors are unclear and need further exploration. Furthermore, the potential value of this receptor as a therapeutic target remains unclear.

##### 5.1.2.6 5‐HT_7_R

Although the roles of 5‐HT_5_R and 5‐HT_6_R in tumor cells have rarely been studied, the role of 5‐HT_7_R in cancer has been demonstrated in several studies [425]. Multiple glioblastoma cell lines (including U‐373MG, H4, CCF‐STTG1, U‐138MG, Hs683, DBTRG‐05MG, T98G, and U‐87MG) express 5‐HT_7_R subtypes [426]. In U‐373MG cells, 5‐HT promotes IL‐6 secretion via 5‐HT_7_R signaling, contributing to tumor progression by fostering a proinflammatory TME [259]. Clinical data have shown that 5‐HT_7_R mRNA expression in prostate tissue is approximately 200 times greater than that in healthy tissue [257]. Cinar et al. [257] reported that 5‐HT_7_R mRNA expression is significantly upregulated in PC‐3 prostate cancer cells. 5‐HT promotes the proliferation of tumor cells and inhibits apoptosis by downregulating the expression of caspase3, caspase9, Bax, and tumor protein 53 (TP53) through 5‐HT_7_R signaling [257]. Similarly, compared with its expression in paratumoral lung tissue, 5‐HT_7_R is overexpressed in non‐small cell lung cancer tumor tissue [260]. This effect of 5‐HT_7_R is mediated through activation of the MAPK and Src signaling pathways [260]. Additionally, 5‐HT_7_R expression is elevated triple‐negative breast cancer (TNBC) cells. 5‐HT activates Src/ERK through 5‐HT_7_R signaling, which promotes tumor cell invasion but does not participate in tumor spread [258]. However, 5‐HT_7_R signaling activates the adenyl cyclase, MAPK, and PI3K/Akt pathways, which inhibit the proliferation of TNBC cells [427].

These findings suggest that blocking 5‐HT_7_R may have significant potential for treating tumors. However, owing to the lack of highly selective drugs that target 5‐HT_7_R, implementing this treatment approach is difficult; thus, developing highly selective antagonists has become a potential therapeutic strategy.

Among the various 5‐HTR subtypes, certain receptors have been assigned greater priority for research and therapeutic use because of their critical roles in tumor progression and potential as drug targets. Among them, 5‐HT_2_Rs have the greatest therapeutic relevance. These receptors are highly expressed in multiple tumor types, and they are involved in regulating cell proliferation, angiogenesis, immune evasion, and even resistance to treatment [20]. The direct roles of 5‐HT_3_Rs in cancer have not yet been fully elucidated; however, emerging studies suggest that they may influence tumor progression [410]. Clinically, 5‐HT_3_R antagonists (e.g., ondansetron) are widely used to alleviate chemotherapy‐induced nausea and vomiting [409], and their indications could be expanded into antitumor applications in the future. 5‐HT_1A_R also represents a high research priority. 5‐HT_1A_R is involved in regulating the cell cycle, apoptosis, and proliferation‐related signaling in cancers such as HCC [420]. The promigratory function of 5‐HT_7_Rs in various cancer cells has made these receptors promising targets for future therapeutic intervention [425]. Currently, the development of drugs that target 5‐HT_7_R is still in its early stages, and further studies are warranted. In summary, on the basis of the current evidence and drug development potential, 5‐HT_2_R, 5‐HT_1A_R, and 5‐HT_7_R represent high‐priority targets in cancer therapy and merit further mechanistic investigation and therapeutic exploration. Although the mechanisms by which 5‐HT_3_R functions in cancer remain to be clarified, its established clinical use has translational potential. Other 5‐HTR subtypes may also contribute to specific cancer types, but further validation is needed, as most studies remain in the exploratory phase.

### 5.2 The 5‐HT System and Functional GI Disorders

In addition to its role in tumors, 5‐HT, which functions as an enteric neurotransmitter and a paracrine signaling molecule, plays a vital role in regulating GI motility and secretion. In the gut, 5‐HT is released primarily by EC cells and exerts paracrine effects on nearby target cells, inducing various physiological responses, such as nausea, vomiting, increased intestinal secretion, and enhanced motility. Dysregulation of the 5‐HT system can lead to abnormalities in GI motility and function, potentially resulting in GI disorders such as chronic constipation, IBS, and carcinoid syndrome.

#### 5.2.1 IBD

IBD comprises two chronic inflammatory conditions of the GI tract with unknown etiology and recurrent episodes: ulcerative colitis (UC) and Crohn's disease (CD). Previous research on the pathogenesis of IBD has focused mainly on immunological and genetic factors; however, studies have also suggested that functional factors are closely involved in its development. Elevated levels of gut‐derived 5‐HT are strongly associated with the onset and progression of IBD [428]. In patients with active IBD, both EC cell numbers and 5‐HT levels are significantly increased [429]. Animal studies have confirmed that abnormalities in the 5‐HT signaling system can lead to the dysregulation of intestinal motility in IBD models [430]. Specifically, the expression of SERT is markedly downregulated in inflamed regions, leading to the local accumulation of 5‐HT, which exacerbates the inflammatory response and is accompanied by an increased number of EC cells [430]. Research has demonstrated that in UC mouse models induced by dextran sulfate sodium (DSS) and CD models induced by trinitrobenzene sulfonic acid, 5‐HT levels are significantly elevated in inflamed colonic tissues [431]. These findings suggest that increased 5‐HT levels may promote inflammatory responses and affect local tissue inflammation, thereby exacerbating colitis [431]. Further studies have shown that reducing 5‐HT expression in the gut can significantly alleviate the severity of colitis in mice, indicating that 5‐HT plays an important regulatory role in the inflammatory process [432]. Moreover, clinical studies have revealed significantly elevated 5‐HT levels in the colonic tissues of UC patients. Experimental interventions involving *Lactobacillus* have been shown to effectively reduce abnormally elevated 5‐HT levels and relieve inflammation in colonic tissues [433, 434]. Clinical studies have shown that blocking 5‐HT_3_Rs can effectively alleviate IBS‐D‐like symptoms that are common in patients with quiescent IBD [435]. This effect is achieved by inhibiting the expression of tight junction proteins in intestinal epithelial cells, thereby increasing intestinal mucosal permeability. As a result, microbial translocation and immune cell activation are triggered, further exacerbating the inflammatory response and mucosal damage associated with IBD. In addition, 5‐HT_7_R antagonists significantly reduce the severity of colonic inflammation and decrease the expression of proinflammatory cytokines in both DSS‐induced colitis and T cell transfer‐induced colitis models [436].

#### 5.2.2 Gastroesophageal Reflux Disease

5‐HT is a key neurotransmitter that plays a crucial role in regulating GI motility, and it is involved in the pathogenesis of gastroesophageal reflux disease (GERD). The functional response of esophageal smooth muscle is mediated by two main signaling mechanisms: receptor‐dependent and receptor‐independent mechanisms. Among these pathways, the receptor‐dependent pathway includes signal transduction mediated by ACh and 5‐HTRs. In children with aromatic Tph deficiency, the conversion of tryptophan to 5‐HT is impaired, resulting in significantly reduced 5‐HT levels and subsequent esophageal motility disorders; thus, Tph deficiency contributes to the development of GERD [437]. Histological studies have shown that in patients with reflux esophagitis, 5‐HT levels are significantly elevated in diseased tissues, whereas SERT mRNA and 5‐HT_4_R expression is markedly decreased in both reflux esophagitis and nonerosive reflux disease [438]. Further research has demonstrated that inhibition of 5‐HT_4_R activity can weaken the contraction of the lower esophageal sphincter, thereby increasing the risk of gastric content reflux [439].

Thus, 5‐HT_4_R agonists may be novel therapeutic options for GERD patients who are unresponsive to proton pump inhibitors. Moreover, selective blockade of specific 5‐HTR subtypes may help reduce intestinal inflammation, indicating potential clinical value in the comprehensive treatment of GERD.

#### 5.2.3 IBS

IBS is among the most common functional GI disorders, it and is characterized primarily by abdominal pain, altered bowel habits, and abnormal stool consistency, with symptoms that are either persistent or intermittently recurrent [440]. In recent years, the understanding of IBS has shifted from a purely biological model to a more integrated biopsychosocial‐genetic model [441]. Among the various mechanisms proposed, abnormalities in the 5‐HT signaling system have emerged as a prominent focus of research worldwide [442].

Previous studies have shown that the mRNA expression of SERT and Tph1 is reduced in IBS patients, leading to impaired 5‐HT reuptake and elevated plasma 5‐HT levels. This increase may contribute to the development of visceral hypersensitivity, which is considered a key mechanism underlying the pathology of IBS [5]. Furthermore, chronic psychological stress can disrupt the gut–brain axis, increasing the number of EC cells in the intestinal mucosa and increasing both the synthesis and release of 5‐HT [443]. Acting through various 5‐HTR subtypes that are located in the myenteric and submucosal plexuses, 5‐HT increases gut sensitivity and worsens IBS symptoms [443]. A study revealed that fasting plasma 5‐HT levels are significantly greater in IBS patients than in healthy controls and that this elevation is positively correlated with the numbers of mast cells and the severity of abdominal pain [444]. These findings suggest that increased 5‐HT release may trigger immune responses in the mucosa, contributing to the sensation of pain. Overall, visceral hypersensitivity is recognized as a core pathophysiological feature of IBS, and elevated 5‐HT levels play a crucial role in its development [444].

Moreover, gut microbiota‐derived metabolites also significantly influence the pathogenesis of IBS [445, 446]. The microbial metabolite acetone can promote 5‐HT release, increase plasma 5‐HT levels, inhibit intestinal water reabsorption, and subsequently cause diarrhea and visceral hypersensitivity [447, 448]. Additionally, short‐chain fatty acids, which are key microbial fermentation products, have been shown to stimulate 5‐HT secretion, increase colonic smooth muscle contraction, and accelerate colonic transit [449]. Studies have also reported that in IBS‐C, probiotic therapy increases intestinal 5‐HT secretion and leads to significant symptom amelioration. Conversely, in IBS‐D, the inhibition of 5‐HT synthesis and release has proven effective in controlling symptoms [450]. These findings suggest that the modulation of 5‐HT signaling could be a potential strategy for the individualized treatment of patients with IBS. Specifically, suppressing 5‐HT synthesis and release is beneficial for IBS‐D patients, whereas increasing 5‐HT levels through probiotics or SSRIs may help alleviate symptoms in IBS‐C patients [450].

At the genetic level, the number of molecular studies on IBS continues to increase. Zhang et al. [451] reported that miRNA‐510 and 5‐HT_3E_R gene expression is significantly increased in the colonic mucosa of Chinese female IBS‐D patients, suggesting that targeting the expression of these molecules may provide therapeutic benefit. Research has shown that polymorphisms in the SERT gene are closely associated with the development of IBS‐C [452]. In a meta‐analysis, Areeshi et al. [453] confirmed that polymorphisms in the SERT gene SLC6A4 are associated with increased susceptibility to IBS among both American and Asian populations.

#### 5.2.4 Functional Dyspepsia

Functional dyspepsia (FD) is a common functional GI disorder. The pathogenesis of FD remains incompletely understood and may involve multiple factors, including GI motility disturbances, visceral hypersensitivity, psychological and social factors, and genetic polymorphisms [454]. Notably, the severity of FD symptoms is not primarily determined by GI pathophysiology, but it is closely associated with psychological factors [455, 456]. Some studies have shown that the S allele of the SERT gene‐linked polymorphic region is significantly associated with the postprandial distress syndrome subtype of FD and is correlated with an increased risk of comorbid psychological symptoms, such as anxiety and depression, in FD patients [457]. However, previous research on FD‐related genetic polymorphisms remains limited, and the genetic mechanisms underlying FD development require further investigation. In recent years, with an increased understanding of the brain–gut axis, SSRIs have gradually been introduced into the clinical management of FD [458, 459]. One study using the SSRI itopride to treat FD patients demonstrated that the drug not only significantly ameliorates dyspeptic symptoms but also effectively alleviates anxiety and depressive states, thereby improving overall quality of life [458]. Nonetheless, SSRIs are also associated with potential risks, including a more than twofold increase in the incidence of GI bleeding, highlighting the need for further large‐scale clinical trials to evaluate their safety profile [460]. FD is also closely associated with chronic psychological stress, which can disrupt brain‒gut axis function and contribute to visceral hypersensitivity [461]. Research suggests that 5‐HT plays a central role in this process by activating specific receptors that regulate neurotransmitter release, increasing GI mucosal and epithelial permeability, and activating pain signaling pathways, ultimately leading to persistent discomfort or pain perception [462]. Abnormal 5‐HT signaling has also been implicated in the pathophysiology of FD. Studies have shown that patients with FD exhibit a significantly attenuated short‐circuit current response to exogenous 5‐HT stimulation. This is accompanied by upregulated expression of 5‐HT_3E_R and SERT and downregulated expression of 5‐HT_7_R and Tph1, suggesting the localized dysfunction of mucosal 5‐HT signaling in patients with FD [463]. A recent meta‐analysis indicated that 5‐HTR agonists can significantly alleviate symptoms in FD patients, with an odds ratio (OR) of approximately 2.99; in particular, these agents can improve early satiety and epigastric fullness [464, 465]. Among these agents, 5‐HT_4_R agonists improve gastric motility, thereby relieving symptoms related to delayed gastric emptying. Furthermore, studies have shown that FD patients exhibit reduced 5‐HT_3_R activity and increased 5‐HT_4_R expression in their stomachs, further suggesting a pivotal role of altered 5‐HT signaling in FD pathogenesis and treatment [466, 467, 468].

Therefore, increasing plasma 5‐HT levels and enhancing 5‐HT activity may be effective therapeutic strategies for treating FD. The combined use of 5‐HTR agonists or SSRIs on the basis of conventional treatment represents a novel direction and therapeutic approach for FD. However, the efficacy and safety of this strategy require further validation in high‐quality clinical trials to support its standardized application in routine clinical practice.

5‐HT plays a significant role in the occurrence and development of functional GI disorders. Drugs that target 5‐HT metabolism and 5‐HTRs represent new agents for treating these diseases. With an increased understanding of the mechanism of action of 5‐HT, more precise and personalized treatment plans are expected to be developed in the future.

### 5.3 The 5‐HT System and Psychiatric Diseases

5‐HTergic neurons regulate a wide range of physiological and behavioral functions and play crucial roles in the onset and progression of various neuropsychiatric disorders [15]. In recent years, in‐depth studies of the serotonergic system, especially its precise mechanisms of action in different brain regions, have increasingly revealed the pivotal role of 5‐HT in mental illnesses [469]. Abnormal 5‐HT signaling is closely associated with numerous neuropsychiatric conditions, including depression, anxiety disorders, bipolar disorder, schizophrenia, autism spectrum disorders, and obsessive‒compulsive disorder [15]. In these disorders, dysfunction of the 5‐HT system typically involves abnormalities in 5‐HT synthesis and metabolism, imbalances in receptor expression, impaired transporter function, and disruptions in downstream signaling pathways [470, 471].

#### 5.3.1 Depression

The 5‐HT h ypothesis of depression posits that depression is associated with impaired serotonergic neuronal function [472]. Studies have demonstrated that alterations in 5‐HTR and SERT, as well as increased activity of presynaptic autoreceptors, are observed in patients with major depressive disorder (MDD) [473, 474]. Moreover, transient depletion of tryptophan in remitted MDD patients can induce a relapse of acute depressive symptoms, further suggesting a critical role of the serotonergic system in depression [475]. SSRIs and other antidepressants exert therapeutic effects by prolonging the half‐life of 5‐HT, thereby alleviating depressive symptoms [14].

Both presynaptic and postsynaptic 5‐HT_1A_Rs are believed to play key roles in the regulation of depression‐like behaviors [476]. Researchers have proposed that selectively targeting 5‐HT_1A_R and 5‐HT_1B_R populations through heteroreceptor activation and autoreceptor blockade may contribute to the antidepressant and antipsychotic effects of certain drugs [477]. Some 5‐HT_1A_R agonists, such as buspirone, have been applied in the clinic to treat depression [478, 479]. Other 5‐HTR subtypes also play important roles in the neurobiology of depression. For example, selective blockade of 5‐HT_2A_R [480], 5‐HT_2C_R [481], and 5‐HT_3_R [482], as well as activation of 5‐HT_2B_R [483] and 5‐HT_4_R [484], is considered to have antidepressant potential. Notably, SSRI treatment activates 5‐HT_2A_R on GABAergic neurons, leading to reduced NE firing [485]. This interaction between 5‐HT_2A_R and the noradrenergic system may explain the limited efficacy of SSRIs in treatment‐resistant depression [480, 486]. In addition, 5‐HT_2C_R antagonism has been shown to increase NE and DA levels in the prefrontal cortex (PFC). Therefore, the suboptimal therapeutic response of some patients to SSRIs may be partially due to the suppression of ventral tegmental area (VTA) dopaminergic activity via the 5‐HT_2C_R [481]. Furthermore, the antidepressant‐like effects of 5‐HT_3_R antagonists are thought to be mediated via the hypothalamic–pituitary–adrenal (HPA) axis [487]. Studies also suggest that both agonists and antagonists of 5‐HT_6_R exert antidepressant‐ and anxiolytic‐like effects in rodent models. However, whether these convergent outcomes occur due to distinct neurochemical effects or to region‐specific brain mechanisms remains unclear. Further research is needed to elucidate the role of 5‐HT_6_Rs in antidepressant responses. Moreover, blockade of 5‐HT_7_R has been proposed to be a promising novel and faster‐acting strategy for treating depression. Interestingly, the inhibition of 5‐HT_7_R has been shown to induce and accelerate antidepressant effects [488]. The microbiota–gut–brain axis is also increasingly being recognized as playing a pivotal role in the pathogenesis of depression [489]. The gut microbiota may help alleviate depressive symptoms by regulating 5‐HT levels [490].

Currently, commonly used antidepressant drugs include tricyclic antidepressants (TCAs), SSRIs, and 5‐HT‐NE reuptake inhibitors (SNRIs). These medications act by increasing the synaptic concentrations of monoamine neurotransmitters, primarily 5‐HT and NE, and activating corresponding postsynaptic receptors [491]. The long‐term use of antidepressants has also been shown to enhance hippocampal neurogenesis. Among TCAs, drugs such as AMI and imipramine, as well as MAOIs, are widely prescribed owing to their safety and tolerability profiles. Recent evidence suggests that SNRIs may have lower tolerability than SSRIs in some patients with depression. However, clinical observations indicate that tolerability varies considerably between individuals. SSRIs are suitable for long‐term treatment and are considered relatively effective at managing moderate to severe depression in adults [492].

#### 5.3.2 Schizophrenia

Schizophrenia is a severe psychiatric disorder that is characterized by chronic or recurrent episodes of psychosis [493]. The clinical manifestations of schizophrenia include positive symptoms (such as hallucinations and delusions), negative symptoms (such as affective flattening or social withdrawal), and cognitive impairments involving attention, memory, and executive function [494]. There is evidence that neurodevelopmental abnormalities that occur during early brain development may represent key factors in the pathogenesis of schizophrenia [495]. Moreover, dysregulation of various neurotransmitter systems is closely associated with the progression of this disorder. In addition to the classical DA hypothesis, chronic stress mediated by the dorsal raphe (DRN) can lead to hyperactivation of the 5‐HT system, thereby disrupting neuronal activity in the cortex, anterior cingulate cortex, and dorsolateral PFC [496]. In addition to changes in 5‐HT activity, postmortem and in vivo studies of patients with schizophrenia have revealed changes in the expression of 5‐HTR and SERT, with most studies focused on the roles of 5‐HT_1A_R and 5‐HT_2A_R. However, findings about the role of 5‐HT_1A_R in schizophrenia remain inconsistent. A meta‐analysis revealed significantly increased 5‐HT_1A_R density in the PFC of patients with schizophrenia [497], whereas another study reported no significant difference in immunoreactivity between patients and healthy controls [498]; these findings indicate that the role of 5‐HT_1A_R in the pathophysiology of schizophrenia remains under debate. In contrast, the role of 5‐HT_2A_R in the pathology of schizophrenia has been extensively documented [295]. Studies have demonstrated significantly reduced 5‐HT_2A_R binding potential in the PFC of patients with schizophrenia compared with that in healthy individuals, suggesting that downregulation or decreased activity of 5‐HT_2A_R may contribute to this disorder [499]. These receptors regulate DA release in the nigrostriatal pathway; the administration of 5‐HT_2A_R, such as olanzapine and risperidone, can increase striatal DA release by blocking the inhibitory effect of 5‐HT [500, 501]. Moreover, excessive activation of 5‐HT_2A_R may increase the release of glutamate in the ventral tegmental area (VTA), which activates the mesolimbic pathway and leads to excessive DA levels in the ventral striatum [502]. Selective 5‐HT_2A_R antagonists have largely failed as monotherapies for schizophrenia, whereas multitarget antagonists with greater affinity for 5‐HT_2A_R than DA for D2R have proven more effective [503].

5‐HT_3_R and 5‐HT_6_R have emerged as promising therapeutic targets for the development of antipsychotic agents, especially for ameliorating the cognitive deficits that are associated with schizophrenia [504, 505, 506]. Studies have shown that the 5‐HT_3_R antagonist ondansetron, when used as an adjunctive treatment, can alleviate symptoms in patients with treatment‐resistant disease and enhance visual memory performance [506, 507]. In addition, preclinical studies have demonstrated that blockade of 5‐HT_5_Rs may have beneficial effects on negative symptoms and cognitive deficits in patients with schizophrenia [508]. 5‐HT_7_R, which has high affinity for several antipsychotic and antidepressant drugs, has also been identified as a potential target [324]. Postmortem studies have revealed reduced expression of 5‐HT_7_R in the PFC of patients with schizophrenia, and polymorphisms in the 5‐HT_7_R gene are significantly associated with this disorder [509]. Blockade of 5‐HT_7_R may help alleviate the negative symptoms of schizophrenia and has been shown to ameliorate ketamine‐induced social withdrawal behavior in mice [510].

Research has indicated that SERT density is not significantly decreased in patients with schizophrenia [511, 512]. These findings suggest that SERT may play a limited role in the pathophysiology of schizophrenia.

In summary, accumulating evidence suggests the presence of serotonergic dysfunction in individuals with depression and schizophrenia. However, further research is needed to develop 5‐HT‐based pharmacotherapies that specifically target the mechanisms underlying this disorder.

### 5.4 The 5‐HT System and Hepatobiliary Diseases

5‐HT not only regulates mental disorders in the CNS but also plays an important role in the periphery. In recent years, the role of 5‐HT in various pathological and physiological processes of the liver, as well as its underlying mechanisms, has attracted significant attention. 5‐HT plays a role in promoting liver regeneration and is closely associated with liver diseases such as viral hepatitis, liver fibrosis, cirrhosis, nonalcoholic steatohepatitis (NASH), primary liver tumors, liver ischemia‒reperfusion injury, and chronic cholestasis [380, 513].

#### 5.4.1 Liver Regeneration

5‐HT not only functions as a neurotransmitter in the nervous system but also promotes cell mitosis and participates in tissue remodeling. Multiple studies have shown that 5‐HT plays an important regulatory role in hepatocyte proliferation during liver regeneration [514, 515, 516]. Platelets are the primary carriers of 5‐HT in the bloodstream, and they transport it to various organs. In a mouse model of liver regeneration following partial hepatectomy (PH), a reduction in platelet count or inhibition of platelet activity significantly suppresses hepatocyte proliferation [516, 517]. In Tph1^−/−^ mice, which lack peripheral 5‐HT, hepatocyte proliferation is markedly reduced. After PH, Tph1^−/−^ mice exhibit impaired liver regeneration and more severe liver injury, which are accompanied by significant upregulation of the transcriptional regulator Yap [518]. The inhibition of Yap expression significantly diminishes the proliferative effect mediated by 5‐HT, suggesting that 5‐HT promotes liver regeneration by regulating Yap expression [518, 519]. In addition, 5‐HTRs plays crucial roles in this process. In the livers of mice subjected to PH, the mRNA expression levels of 5‐HT_2A_R and 5‐HT_2B_R increase, which contributes to liver regeneration [520]. The underlying mechanism involves the IL‐33/growth stimulation expressed gene 2 (ST2) pathway‐mediated release of gut‐derived 5‐HT into the bloodstream, which subsequently activates the 5‐HT_2A_R/p70S6K signaling pathway in hepatocytes [521]. Further research indicated that 5‐HT_2_R may act as a cofactor in DNA synthesis, enhancing hepatocyte proliferation by promoting the G1/S phase cell cycle transition. Moreover, the 5‐HT_7_R receptor is involved in hepatocyte mitosis and plays a role in liver regeneration [256]. In animals subjected to PH, elevated 5‐HT levels in the brainstem and cerebral cortex, along with the upregulation of 5‐HT_2C_R receptors, suggest that this pathway may indirectly promote hepatocyte proliferation by stimulating sympathetic nerve activity [522].

#### 5.4.2 Liver Fibrosis and Cirrhosis

Activation of hepatic stellate cells (HSCs) is a key step in the initiation and progression of liver fibrosis. 5‐HT regulates the activation, proliferation, and apoptosis of HSCs by binding to various receptors that are expressed on their surface, thereby influencing the fibrotic process. Multiple 5‐HTR subtypes are upregulated in activated HSCs and are predominantly distributed within fibrotic tissues [523]. Among these receptors, 5‐HT_2A_R plays a critical role in HSC‐mediated fibrosis [524]. In vitro experiments have indicated that activation of 5‐HT_2A_R can stimulate the Smad signaling pathway, leading to changes in the expression of fibrosis‐related markers, such as α‐smooth muscle actin (α‐SMA) and collagen [102, 524]. Additionally, 5‐HT can activate the ERK and JunD signaling pathways via 5‐HT_2B_R, upregulate TGF‐β1 expression, and synergize with PDGF signaling to promote HSC activation [525, 526]. 5‐HT_2_R antagonists significantly inhibit HSC proliferation and induce apoptosis while downregulating the expression of key fibrogenic proteins, including TGF‐β1, PKC, p‐Smad3, and p‐ERK1/2, in liver tissue, thus effectively alleviating liver fibrosis [527]. Moreover, emerging evidence suggests that 5‐HT_7_R may have opposing effects on HSC function. Ruddell et al. [528] reported a marked reduction in 5‐HT_7_R mRNA expression in activated rat HSCs. Similarly, Polat et al. [529] reported decreased hepatic expression of 5‐HT_7_R in a CCl_4_‐induced mouse model of cirrhosis. Activation of 5‐HTRs attenuates liver fibrosis and cirrhosis and slows HCC progression [529]. Notably, HSCs also express SERT, which allows them to actively take up and release 5‐HT, forming an autocrine/paracrine regulatory loop that helps maintain the local stability and persistence of 5‐HT signaling [528].

In summary, 5‐HT may play dual roles in the development of liver fibrosis and cirrhosis through different receptor subtypes. On the one hand, 5‐HT promotes HSC activation and proliferation via 5‐HT_2_R, thereby facilitating fibrogenesis; on the other hand, 5‐HT may exert antifibrotic effects through 5‐HT_7_R. The differential expression patterns and regulatory mechanisms of various 5‐HTRs in distinct hepatic cell populations during the progression of fibrosis warrant further investigation.

#### 5.4.3 NASH

5‐HT plays a critical role in the pathogenesis of NASH [530]. 5‐HT levels are significantly elevated in both the peripheral blood and portal veins of NASH patients, suggesting a close association with disease onset [531]. Research has indicated that MAO‐A expression is upregulated in patients with NASH [531]. In methionine–choline‐deficient diet‐induced animal models of NASH, 5‐HT is metabolized by MAO‐A in mitochondria, leading to the production of large amounts of ROS and lipid peroxides [532]. This oxidative stress results in mitochondrial dysfunction and triggers inflammatory responses, thereby exacerbating hepatocellular injury and the pathological progression of NASH [532]. Moreover, inhibition of MAO‐A activity in wild‐type mice alleviates liver damage. Although Tph1^−/−^ mice exhibit levels of hepatic steatosis that are similar to those in wild‐type controls, impaired peripheral 5‐HT synthesis in these mice leads to reduced ROS production and milder hepatocellular injury, consequently attenuating liver inflammation [533]. In addition, 5‐HT contributes to NASH progression by activating 5‐HT_2A_R on hepatocyte membranes, thereby disrupting lipid metabolism and promoting hepatic fat accumulation and inflammatory responses [534]. The underlying mechanisms involve the upregulation of peroxisome proliferators‐activated receptors (PPARs) γ2 and lipogenesis‐related genes, such as factor‐related apoptosis (Fas), Cd36, and Perilpin2, which increase lipid synthesis and deposition [535]. Studies have demonstrated that hepatosteatosis is significantly reduced in intestinal‐specific Tph1^−/−^ mice and liver‐specific 5‐HT_2A_R^−/−^ mice that are fed a high‐fat diet (HFD); these results suggest that inhibiting gut‐derived 5‐HT synthesis or blocking hepatic 5‐HT_2A_R signaling can ameliorate lipid metabolism disorders [536]. Furthermore, 5‐HT_2A_R antagonists can prevent HFD‐induced hepatic steatosis [537], and the combined use of 5‐HT synthesis inhibitors and 5‐HT_2_R antagonists markedly reduces hepatic steatosis and inflammation in mouse models of diabetes [535, 538]. Moreover, studies have revealed that 5‐HT_4_R expression in both liver and adipose tissue significantly decreases after HFD intervention in mice. Blocking 5‐HT_4_R not only mitigates hepatic steatosis but also reduces adipose tissue mass and suppresses proinflammatory cytokine expression and inflammasome complex formation, highlighting a potential anti‐inflammatory and metabolic regulatory effect [539].

In addition to its direct hepatic effects, 5‐HT plays an essential role in modulating intestinal barrier function. 5‐HT_3_R regulates the expression of tight junction proteins such as claudin‐1 (CLDN1) and occludin in intestinal epithelial cells. Disruption of 5‐HT_3_R activity increases gut permeability, allowing LPS to enter the liver, where it activates immune responses and accelerates the transition from NAFLD to NASH [540]. Additionally, the gut microbiota participates in the pathogenesis of NASH by regulating the levels of 5‐HT and its metabolites. Gut bacteria can influence 5‐HT synthesis by metabolizing tryptophan and modulating the inflammatory status of the gut–liver axis, thereby indirectly affecting hepatic lipid accumulation and inflammation, which further exacerbates the progression of NASH [541].

In conclusion, 5‐HT is involved in various aspects of NASH pathology, including lipid metabolism dysregulation, oxidative stress, inflammatory activation, and disruption of the gut–liver barrier, via multiple targets and signaling pathways. These multifaceted mechanisms reveal novel targets and strategies for NASH treatment. Future interventions that focus on the comprehensive regulation of 5‐HT synthesis, metabolism, and receptor signaling may offer promising directions for the effective treatment of NASH.

#### 5.4.4 Viral Hepatitis

Clinical studies have shown that serum 5‐HT levels in patients with hepatitis C virus (HCV) infection who are receiving interferon antiviral therapy can serve as a predictor of treatment efficacy [542]. Patients with higher 5‐HT levels tend to respond better to interferon treatment, suggesting that 5‐HT may be closely associated with the onset and progression of viral hepatitis [543]. In mouse models of viral hepatitis, the number of activated platelets in the liver is significantly increased [544]. These platelets release 5‐HT, which can reduce hepatic blood flow, impair the recruitment of CD8^+^ T cells that are needed for viral clearance, and thus exacerbate liver injury. In contrast, Tph1^−/−^ mice, which lack the ability to synthesize 5‐HT in the periphery, exhibit significantly reduced liver damage; these results further suggest a pathogenic role of 5‐HT in viral hepatitis [545]. Additionally, studies have shown that SSRIs, such as fluoxetine, can effectively inhibit platelet uptake of 5‐HT, thereby reducing circulating 5‐HT levels. This phenomenon improves hepatic microcirculation, enhances CD8^+^ T cell recruitment, and accelerates hepatitis viral clearance [545]. Clinical observations have also shown that SSRIs can reduce the risk of HCC in patients with hepatitis B virus (HBV) infection in a dose‐dependent manner [546]. Moreover, in vitro studies involving HCV‐infected hepatocyte cultures have suggested that 5‐HTR activation participates in the viral entry process. Specifically, the activation of 5‐HT_2A_R enhances the membrane localization of CLDN1, which is a key tight junction protein, thereby promoting endocytosis and facilitating HCV entry into cells [542].

In conclusion, 5‐HT plays a crucial role in the occurrence and progression of various liver diseases. Modulating the level of 5‐HT or blocking signaling pathways mediated by its receptors may provide new therapeutic approaches and potential therapeutic targets for liver diseases.

### 5.5 The 5‐HT System and Diabetes

5‐HT, which is a key neurotransmitter, also plays a widespread role in regulating energy metabolism, particularly in the maintenance of glucose homeostasis, in which it performs dual functions. On the one hand, some studies have reported that 5‐HT can increase blood glucose levels and promote insulin resistance; on the other hand, evidence suggests that 5‐HT can stimulate insulin secretion, increase insulin sensitivity, and ameliorate metabolic disorders.

The hyperglycemic effect of 5‐HT is likely mediated by 5‐HT_2_R. Gut‐derived 5‐HT can act on 5‐HT_2B_R in hepatocytes, thereby promoting gluconeogenesis [547, 548]. Blocking 5‐HT_2B_R improves glucose metabolism and facilitates the restoration of normoglycemia [549, 550]. When 5‐HT binds to 5‐HT_2_R, it induces serine phosphorylation of insulin receptor substrate‐1, reduces its activity, and promotes its ubiquitin‐mediated degradation, thereby disrupting insulin signaling pathways [551]. On the basis of these mechanisms, 5‐HT_2_R antagonists have been explored in the clinic as potential treatments for type 2 diabetes and metabolic syndrome.

Recent studies have also revealed the hypoglycemic effects of 5‐HT. Animal experiments have shown that 5‐HT increases serum insulin levels, reduces plasma glucose concentrations, improves glucose tolerance and lipid metabolism in mice with type 2 diabetes, and reduces body weight in a dose‐dependent manner [552]. Additionally, 5‐HT activates 5‐HT_7_R in the adrenal gland, promoting the release of β‐endorphin, which in turn activates opioid receptors, enhances peripheral glucose uptake and utilization, and significantly decreases blood glucose levels in type 1 diabetes models [553]. Clinical studies have shown that 5‐HT_4_R agonists also exert glucose‐lowering effects, primarily by increasing insulin sensitivity [554]. Furthermore, 5‐HT activates 5‐HT_2A/2C_R, increasing the membrane expression of glucose transporters (e.g., GLUT4) and thereby promoting glucose uptake by cardiomyocytes [555]. In addition, Tph1^−/−^ mice, which lack peripheral 5‐HT, exhibit reduced insulin secretion and elevated blood glucose levels, highlighting the crucial role of 5‐HT in pancreatic islet function [556].

In addition to insulin signaling, 5‐HT plays important roles in regulating insulin secretion and β‐cell growth. 5‐HT is stored along with insulin in pancreatic β‐cell granules, and it is released simultaneously in response to glucose stimulation. Studies suggest that intracellular 5‐HT can regulate the fusion of insulin‐containing vesicles with the cell membrane through serotonylation, thus facilitating insulin secretion [551]. Extracellular 5‐HT can regulate insulin release via the activation of 5‐HT_1A_R. Additionally, 5‐HT may act in an autocrine or paracrine manner to activate 5‐HT_2B_R, thus promoting β‐cell proliferation [557]. 5‐HT also suppresses the expression of α‐cell markers (such as Arx and Gcg) while increasing β‐cell marker expression in murine α‐cell lines, suggesting its potential to induce α‐to‐β‐cell phenotype conversion [558].

In addition to its direct effects on glucose metabolism, 5‐HT is involved in the regulation of diabetic complications. Peripheral 5‐HT_5A_R has been shown to play a significant role in inhibiting cardiac sympathetic nerve activity in rats with type 1 diabetes [559]. The 5‐HT_1A_R agonist NLX‐112 has been shown to ameliorate urinary dysfunction in rats with diabetes, and this mechanism is likely associated with the upregulation of 5‐HTR expression in the L6–S1 spinal dorsal lateral nucleus [560]. Treatment with a 5‐HT_2B_R agonist has been shown to improve colonic migrating motor complex activity in males with diabetes and enhance colonic transit in ovariectomized female patients with diabetes [561]. In type 1 diabetes models, the downregulation of presynaptic 5‐HT_1A_R enhances spinal 5‐HT release, reduces quinolinic acid levels, inhibits tryptophan 2,3‐dioxygenase (Tdo), indoleamine 2,3‐dioxygenase (Ido) 1, and Ido2 expression, and improves neuronal degeneration and pain‐related behaviors [562]. Furthermore, the contractile response of the pancreatic and mesenteric arteries to 5‐HT is significantly reduced in mice with diabetes, possibly due to decreased 5‐HTR activity and increased eNOS activation, leading to elevated nitric oxide release and reduced vascular tone [563].

In summary, the 5‐HT signaling pathway, which is a complex and highly tunable biological network, includes factors that are emerging as therapeutic targets for a wide range of systemic diseases. Future research should continue to integrate multiomics data, drug screening platforms, and clinical validation to advance the development of 5‐HT‐related therapies toward precision medicine, combination treatments, and targeted delivery, thereby promoting translational advancements in disease management.

## 6 Role of 5‐HT in Tumor Immunity

**FIGURE 5:**
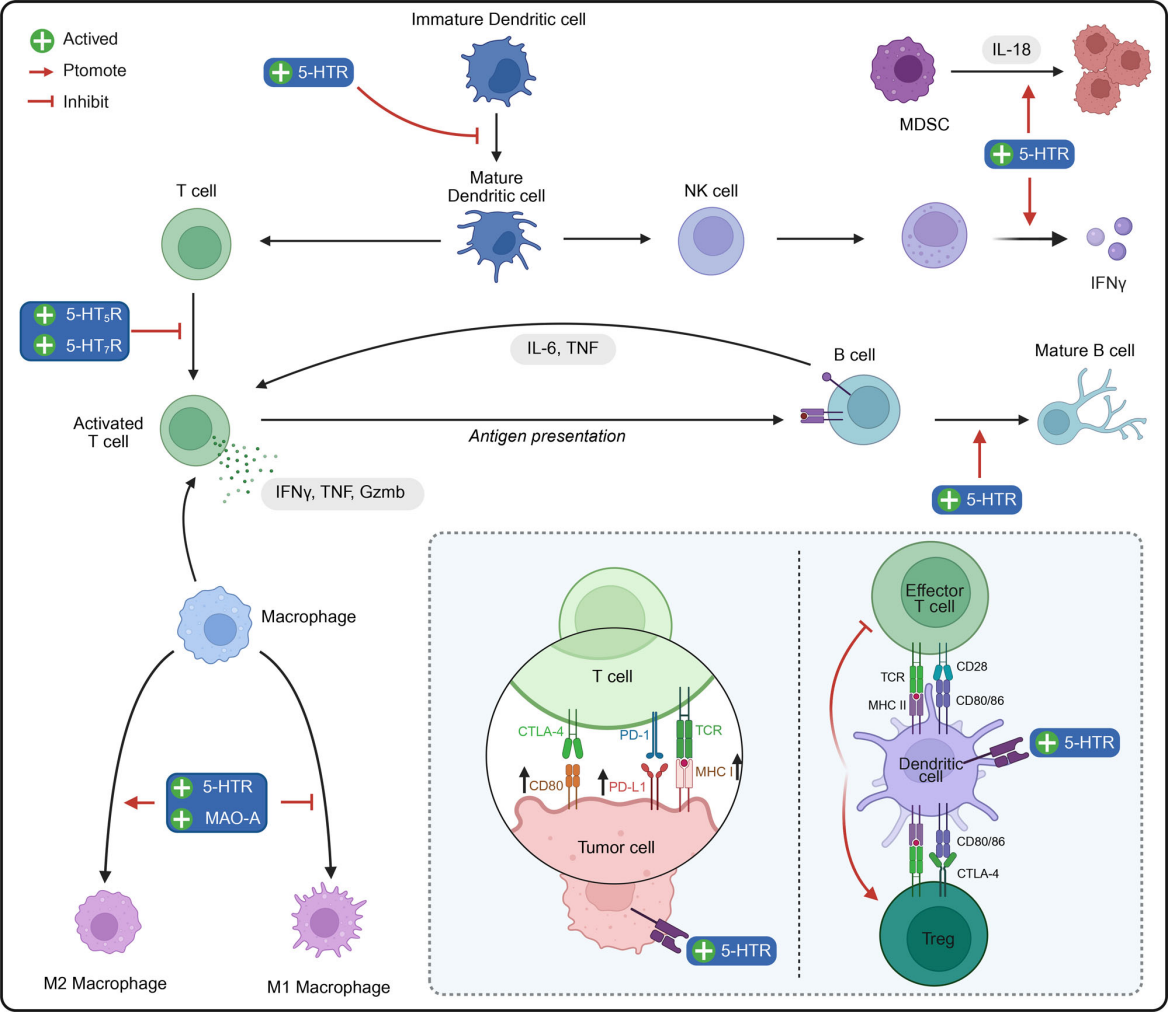
Role of serotonin (5‐HT) signaling in tumor immunity and mechanistic cross‐talk between 5‐HT signaling and immune checkpoints. 5‐HT acts on 5‐HT receptors (5‐HTRs) on the surfaces of immune cells, promotes dendritic cell (DC) maturation, stimulates T‐cell activation, increases NK cell cytotoxicity, promotes B‐cell maturation, and stimulates macrophage polarization toward the M2 phenotype.

In addition to its role in a variety of diseases that have been widely reported or applied in clinical treatment, an increasing number of studies in recent years have focused on the function of 5‐HT in immune regulation. Owing to its multifaceted effects on immune cells, the 5‐HT signaling axis represents a promising direction for research on tumor immunity (Figure 5 and Table 3).

**TABLE 3:** Roles of 5‐HT in tumor immunity.

Receptor	Cell type	Mechanism	Effect	References
5‐HT_1A_R	T cells, B cells, NK cells	5‐HT binds to 5‐HTR and increases the intracellular cAMP level in response to ATP in T cells. 5‐HT binds to 5‐HTR and increases IL‐10 production in B cells through the activation of STAT3 activation. 5‐HT protects NK cells from peroxidase inhibition.	T cell proliferation and stimulation B cell proliferation Promotes NK cell activation and IFN‐γ production by NK cells	[564, 565, 566]
5‐HT_1B_R	DCs	5‐HT induces 5‐HTR‐dependent Ca^2+^ influx in immature and mature DCs.	DC migration	[301]
5‐HT_1E/1F_R	DCs	5‐HT induces a 5‐HTR‐dependent intracellular Ca^2+^ spike in immature DCs.	DC migration	[301]
5‐HT_2_R	DCs, T cells, TAMs	5‐HT stimulates 5‐HTR to induce intracellular Ca^2+^ mobilization via Gi/o proteins in immature DCs and promote TLR3 activation. 5‐HT mediates the phosphorylation of the ERK1/2 and Iκβα in T cells. 5‐HT stimulates 5‐HTR to promote the ERK pathway and AhR axis activation.	Immature CD1a^+^ monocyte‐derived DC maturation T cell proliferation and stimulation M2 macrophage polarization	[242, 301, 302]
5‐HT_3_R	DCs, T cells	5‐HT induces a 5‐HTR‐dependent intracellular Ca^2+^ spike in immature DCs. 5‐HT stimulates 5‐HTR and increases intracellular Na^+^ concentrations.	DC maturation T cell activation	[301]
5‐HT_4_R	DCs	5‐HT activates 5‐HTR to induce an increase in cAMP levels in mature DCs, increase the release of the cytokines IL‐1β and IL‐8, and reduce the secretion of IL‐12 and TNF‐α.	DC maturation	[301]
5‐HT_1/4/7_R	DCs	5‐HT binds to 5‐HTR in DCs cells to promote the production of the pro‐inflammatory cytokine IL‐6, inhibit the production of the chemokine IP‐10/CXCL10, increase the secretion of CCL22/MDC, and promote the transformation of mature DCs into high IL‐10 and low IL‐12/p70 secretion phenotypes.	Cytokine release from DCs	[567]
5‐HT_7_R	DCs, T cells, TAMs	5‐HT binds to 5‐HTR on monocytes, induces changes in DC phenotypes, decreases the expression of costimulatory molecules and CD1a, increases the expression of CD14, significantly decreases the stimulating activity of allogeneic T cells, and affects small GTPase and Cdc45 activation. 5‐HT binds to 5‐HTR to induce the phosphorylation of ERK1/2 and IκBα in naive T cells. 5‐HT binds to 5‐HTR to inhibit the release of TNF‐α and IL‐12/p40 from monocyte‐derived macrophages stimulated by LPS.	Maturation and migration of bone marrow‐derived DCs T cell activation TAM polarization toward an anti‐inflammatory phenotype and infiltration	[242, 341, 568]

### 6.1 5‐HT‐Mediated Regulation of Tumor‐Infiltrating Immune Cells

A variety of 5‐HTRs are expressed on the surfaces of immune cells [569]. 5‐HT interacts directly with distinct 5‐HTRs on immune cells that can profoundly influence the ability of the immune system to target and eliminate tumors.

#### 6.1.1 Macrophages

Macrophages, particularly TAMs, are critical targets of 5‐HT signaling within the TME. Macrophages play pivotal roles in modulating inflammation, tissue remodeling, and immune surveillance [570, 571, 572]. Emerging evidence indicates that 5‐HT can influence macrophage polarization and cytokine production, thereby shaping the immune landscape in ways that either support or hinder tumor development.

5‐HT binds to 5‐HTR on the surfaces of monocytes and macrophages, promoting the polarization of TAMs toward the M2 phenotype and regulating their cytokine secretion [573]. Nocito et al. [162] reported that 5‐HT also reduces the expression of MMP12 in TAMs and promotes tumor progression in mice with colon cancer. MMP12 cleaves plasminogen to generate the angiogenesis inhibitor angiostatin, exerting an antiangiogenic effect. However, it has been reported that macrophages respond to stimulation with 5‐HT at different concentrations. Low levels of 5‐HT promote the production of IL‐6 and TNF‐α by macrophages and T cells through 5‐HT_2_R, whereas high concentrations of 5‐HT inhibit the secretion of IL‐6 and TNF‐α, thereby inducing T cells to differentiate into immunosuppressive or nonfunctional types [242, 302].

#### 6.1.2 DCs

In addition to its effects on macrophages, 5‐HT also exerts a significant regulatory effect on DCs, which serve as key antigen‐presenting cells that bridge innate and adaptive immunity. By modulating the phenotype and cytokine profile of DCs, 5‐HT can indirectly shape T‐cell responses and thereby influence tumor progression.

5‐HT signaling promotes the maturation and chemotaxis of bone marrow‐derived DCs [574]. 5‐HT promotes the development of anti‐inflammatory DCs, which in turn may lead to the polarization of Tregs [575]. Tregs often suppress cytotoxic T‐cell activity, thereby promoting tumor development [576]. 5‐HT binds to 5‐HTR on DCs, induces changes in the DC phenotype, decreases the expression of costimulatory molecules and CD1a, increases the expression of CD14, and significantly decreases the stimulating activity of allogeneic T cells [568]. Idzko et al. [301] reported differences in the expression of 5‐HTR on DCs at different stages; immature DCs preferentially express 5‐HT_1B_R, 5‐HT_1E_R and 5‐HT_2B_R, whereas mature DCs mainly express 5‐HT_4_R and 5‐HT7R. The activation of different types of receptors induces different functions in DCs. 5‐HT_1_R and 5‐HT_2_R stimulation induces intracellular Ca^2+^ mobilization via the Gi/o protein in immature DCs. 5‐HT_4_R and 5‐HT_7_R induce elevated cAMP levels in mature DCs. In addition, 5‐HT_4_R and 5‐HT_7_R increase the release of IL‐1β and IL‐8 while decreasing the secretion of IL‐12 and TNF‐α by mature DCs [301].

#### 6.1.3 T Cells

T cells play a direct role in inhibiting and killing tumors [577, 578, 579, 580]. Recent research has highlighted the complex and sometimes contradictory roles of 5‐HT in modulating antitumor immunity, particularly through its influence on the behavior of T cells in the TME. On the one hand, 5‐HT has been shown to inhibit T‐cell function; on the other hand, under certain conditions, it may enhance T cell‐mediated immune responses.

5‐HT can inhibit the proliferation and activity of cytotoxic T cells through 5‐HT_5A_R and 5‐HT7R [581]. The activation of 5‐HT_3_Rs on the surface of T cells by 5‐HT can significantly suppress the CXCL12‐mediated migration of CD4^+^ T cells, indicating that increased 5‐HT levels may prevent the recruitment of T lymphocytes to tumor tissues [341]. The expression of 5HT_2A/2B_R in breast cancer patients is positively correlated with the invasion of CD8^+^ T cells. In zebrafish and mouse models, 5HT_2A_R activation promotes CD8^+^ T‐cell proliferation and inhibits breast cancer invasion and metastasis [582]. CD8^+^ T cells accumulate 5‐HT intracellularly to promote the formation of 5‐HT from GAPDH, thereby promoting the glycolytic metabolism and antitumor immune activity of CD8^+^ T cells [583]. In addition, treatment of chronically stressed mice with 5‐HT reduces the infiltration of CD8^+^ T cells into the TME, and the expression of IFN‐γ and granzyme B in CD8^+^ T cells is also reduced, whereas the expression of PD‐1on CD8^+^ T cells is increased [584]. Other groups have reported that 5‐HT increases PD‐L1 expression by tumor cells in vitro through serotonylation. Moreover, serum 5‐HT concentrations in patients with abdominal tumor metastases are negatively correlated with the number of tumor‐infiltrating CD8^+^ T cells. 5‐HT depletion suppresses the growth of syngeneic pancreatic and colorectal tumors in wild‐type mice, increases CD8^+^ T cell influx, and reduces PD‐L1 expression [212].

#### 6.1.4 Other Cells

5‐HT can increase the cytotoxic potential of NK cells [564, 585]. 5‐HT also regulates the mitogen‐stimulated proliferation of mature B cells [565]. However, the detailed mechanisms by which 5‐HT affects various immune cells via downstream signaling pathways have not been elucidated. In the future, the use of various drugs with the potential to regulate 5‐HT signaling may play a key role in modulating tumor immunity.

### 6.2 5‐HT‐Mediated Tumor Immunotherapy

Studies have shown that 5‐HT can upregulate PD‐L1 expression on the surface of tumor cells, thereby significantly reducing the infiltration of CD8^+^ T cells into the TME and weakening the antitumor immune response. Experimental results demonstrate that eliminating 5‐HT or blocking 5‐HT signaling leads to a reduction in PD‐L1 levels on tumor cells, increased T‐cell infiltration, and slower tumor progression; these results suggest that antidepressants (e.g., SSRIs) in combination with PD‐1/PD‐L1 inhibitors may exert synergistic antitumor effects [212]. In addition, MAOIs can act as immune modulators. Studies have shown that MAO‐A is overexpressed in tumor‐associated immune cells and that its genetic deletion or pharmacological inhibition (e.g., with phenelzine) can significantly increase the antitumor activity of CD8^+^ T cells. In murine tumor models, MAO‐A knockout or inhibition not only markedly suppresses tumor growth but also exhibits synergistic efficacy when combined with anti‐PD‐1 therapy [226]. In the context of CAR T‐cell immunotherapy, which is a novel T cell‐based therapy that involves Tph1‐engineered CAR T cells that can endogenously synthesize 5‐HT, has been developed. These modified cells significantly enhance CD8^+^ T‐cell activation and endoplasmic function. While traditional CAR T cells reduce tumor volume by approximately 32% on day 23, Tph1‐CAR T cells achieve a tumor reduction of approximately 78%, indicating markedly stronger antitumor activity [583]. Furthermore, recent studies in animal models revealed that SSRIs significantly enhance CD8^+^ T‐cell function in various tumor models, including melanoma, breast cancer, prostate cancer, and colorectal cancer models, leading to greater than 50% tumor growth inhibition. More importantly, when combined with PD‐1/PD‐L1 immune checkpoint inhibitors, this treatment resulted in complete tumor regression in all experimental animals, further highlighting the promising clinical translation potential of targeting the 5‐HT signaling pathway [586].

In summary, 5‐HT plays a crucial role in regulating tumor‐infiltrating immune cells, and its signaling axis has emerged as a novel target for tumor immunotherapy. Future studies should focus on elucidating the mechanisms by which different receptor subtypes regulate immune cells and validating their therapeutic potential across various tumor models and clinical settings.

## 7 The Potential of 5‐HT for Use as a Biomarker of Disease

5‐HT, also known as serotonin, is a crucial neurotransmitter, hormone, and paracrine signaling molecule [587, 588]. While it is best known for its role in mood regulation, cognitive function, and GI motility, an increasing number of studies have demonstrated that changes in 5‐HT levels, metabolic pathways, and receptor expression are closely associated with various pathological conditions [39]. As such, interest in the potential of 5‐HT for use as a biomarker of a wide range of diseases, particularly psychiatric disorders, inflammatory conditions, cancer, and metabolic syndromes, is increasing [589].

Decreased 5‐HT signaling in the CNS is considered a key mechanism underlying depressive symptoms [590]. 5‐HT levels, such as those in plasma or platelets, have been used as dynamic indicators for evaluating depressive states [591]. Some studies have reported significantly lower levels of 5‐HT in the cerebrospinal fluid of patients with depression than in that of healthy individuals, suggesting the potential of 5‐HT levels to be used as a diagnostic or prognostic biomarker [592]. However, findings on the effects of antidepressant treatment on peripheral 5‐HT levels remain inconsistent. A meta‐analysis of 15 clinical trials and 11 animal studies revealed that peripheral 5‐HT levels may increase, decrease, or remain unchanged after pharmacological intervention [593, 594]. This high degree of variability currently limits the reliability of peripheral 5‐HT levels as a stable biomarker for diagnosing depression or evaluating therapeutic efficacy.

5‐HT plays a critical role in GI motility, secretion, and local immune regulation [595]. Studies have shown that elevated 5‐HT levels in the GI mucosa or peripheral blood are strongly associated with conditions such as IBS, IBD, and carcinoid syndrome [596]. In patients with CD, serum 5‐HT levels are significantly elevated during phases of active disease [597, 598]. Moreover, serum 5‐HT levels outperform traditional inflammatory markers, such as C‐reactive protein and circulating cytokine levels, in distinguishing active versus refractory or remissive disease states [599]. Thus, the serum 5‐HT level is considered an effective biomarker for disease stratification and treatment guidance in patients with CD [600]. Additionally, 5‐HT is involved in platelet aggregation and vasoconstriction, indicating its potential clinical relevance in cardiovascular diseases such as hypertension, atherosclerosis, and myocardial infarction [601, 602, 603].

A growing body of evidence highlights the role of 5‐HT in tumor biology [604]. In addition to supporting tumor cell proliferation and angiogenesis, 5‐HT may promote immune evasion and metastasis. In solid tumors such as colorectal, breast, and prostate cancers, the overexpression of 5‐HTRs (e.g., 5‐HT_2A_R and 5‐HT_2B_R) and increased SERT levels have been correlated with tumor progression, stage, and prognosis [604, 605, 606]. Dynamic changes in the 5‐HT levels in blood and tumor tissues may serve as indicators for monitoring therapeutic response and recurrence risk [606]. In patients with carcinoid syndrome, urinary 5‐HIAA, which is an end metabolite of 5‐HT, has already become a clinically established biomarker for assessing tumor burden and monitoring treatment efficacy [607].

5‐HT also plays a pivotal role in modulating the immune system. Studies have shown that 5‐HT influences the functions of DCs, macrophages, and T and B lymphocytes [608]. In autoimmune diseases such as systemic lupus erythematosus, rheumatoid arthritis, and multiple sclerosis, abnormal expression of 5‐HTRs on immune cells may affect disease progression, suggesting the potential of these receptors to be used as supplementary biomarkers for monitoring disease activity [609, 610]. In the context of metabolic disorders, particularly type 2 diabetes and obesity, 5‐HT has been shown to regulate glucose metabolism, insulin sensitivity, and adipose tissue inflammation [611]. Studies have shown that changes in 5‐HT levels or disruptions in its metabolic pathway are associated with insulin resistance and chronic inflammation in adipose tissue, indicating that 5‐HT can be used as a potential biomarker for the early diagnosis of diabetic complications and related disorders [611].

Overall, the widespread involvement of 5‐HT and its signaling pathways across numerous physiological and pathological processes highlights its tremendous potential in biomarker development. Through direct measurement (e.g., serum or CSF levels of 5‐HT and 5‐HIAA), functional imaging (e.g., PET scans of SERT distribution), or functional assessment (e.g., receptor sensitivity and transporter expression), 5‐HT‐related indicators hold promise for early disease screening, disease progression monitoring, therapeutic response prediction, and the development of personalized treatment strategies [612]. However, the expression of 5‐HT is influenced by various factors, such as age, sex, diet, circadian rhythms, and methodological differences in sample collection and analysis. Therefore, further large‐scale and standardized studies are needed to optimize its clinical application.

## 8 Conclusion and Prospects

This review provides a comprehensive overview of the 5‐HT system, emphasizing 5‐HT metabolic pathways, 5‐HT receptor‐mediated signaling mechanisms, and the complex and widespread roles of 5‐HT across various physiological and pathological contexts. 5‐HT has multiple biological effects on emotional regulation, GI homeostasis, and immune modulation, and it is also involved in the development and progression of numerous diseases, including psychiatric disorders, metabolic syndrome, inflammatory conditions, and cancer [17, 18, 19]. In recent years, aberrant 5‐HT signaling has been increasingly recognized as a key feature of various pathologies, particularly neuroinflammation, tumor immune evasion, and vascular dysfunction [589]. While several therapeutic agents that target the 5‐HT system, such as SSRIs, MAOIs, and receptor‐specific antagonists, have been successfully applied in clinical practice [221, 279, 290], their broader implementation is hindered by limited target selectivity, systemic complexity, and frequent adverse effects.

Several fundamental scientific questions remain unresolved. The mechanisms by which 5‐HT regulates immune cell functions, such as macrophage polarization and T‐cell activation, within the tumor immune microenvironment remain poorly defined, and the causal relationships between 5‐HT signaling and immune activation or suppression require further clarification. Additionally, the interplay between 5‐HTRs and key signaling pathways (e.g., Wnt/β‐catenin and PD‐1/PD‐L1) is a potential basis for the dual tumor‐promoting and tumor‐inhibiting effects of 5‐HT, and this interplay warrants systematic investigation. Moreover, the influence of the gut microbiota on peripheral 5‐HT synthesis and metabolism has emerged as a novel research topic [613]. Increasing evidence suggests that microbial metabolites can modulate tryptophan metabolism in the gut, thereby regulating EC cell‐derived 5‐HT production, which in turn affects host neuroendocrine and immune homeostasis through the gut–brain–immune axis [614]. Thus, microbially mediated regulation of 5‐HT metabolism may act as a mechanistic link in the pathogenesis of autoimmune diseases and mood disorders.

Although preclinical studies have demonstrated the therapeutic potential of targeting 5‐HT pathways, significant barriers remain in translating these findings into clinical applications. On the one hand, the bidirectional role of 5‐HT in different disease contexts, especially in cancer, poses considerable challenges for drug design. On the other hand, currently available pharmacological agents lack receptor subtype selectivity and tissue specificity, which results in systemic side effects. The development of highly selective receptor modulators represents a promising direction for enhancing therapeutic specificity and minimizing off‐target effects. Immunometabolic combination strategies should also be prioritized, including the use of SSRIs to increase CD8^+^ T‐cell function in synergy with PD‐1/PD‐L1 immune checkpoint blockade. Future research to engineer CAR T cells with the ability to locally modulate 5‐HT signaling may offer new approaches to reshape the immunosuppressive TME. Alternatively, targeted nanocarrier systems could enable the precise delivery of 5‐HTR antagonists to specific tissues, thereby reducing systemic toxicity. Spatial and temporal modulation of 5‐HT levels, for example, by enhancing 5‐HT signaling in the gut while suppressing it in the CNS, could represent a strategy to maximize therapeutic efficacy while minimizing side effects.

In summary, 5‐HT signaling represents a central interface that links metabolic control, receptor activation, and immune responses across a spectrum of pathophysiological states. Therapeutically targeting the 5‐HT system not only advances mechanistic insights into disease progression but also offers a framework for the development of integrative, multitarget treatment strategies. Continued research into the biology of 5‐HT is expected to accelerate the discovery of novel therapeutics, ultimately improving the management of cancer, neuropsychiatric disorders, GI dysfunction, and metabolic disease.

## Author Contributions

**Yuxin Zhang**: conceptualization, methodology, investigation, data curation, writing – original draft, and visualization. **Nan Wang**: methodology. **Louqian Zhang**: methodology. **Yan Zhuang**: methodology. **Qilei Xin**: investigation. **Xiaosong Gu**: supervision, visualization, project administration, and funding acquisition. **Chunping Jiang**: supervision, visualization, project administration, and funding acquisition. **Junhua Wu**: supervision, visualization, writing – review and editing, project administration, and funding acquisition. All the authors have approved the final version of this manuscript.

## Conflicts of Interest

The authors declare no conflicts of interest.

## Ethics Statement

The authors have nothing to report.

## Data Availability

No data were used for the research described in the article.

## References

[1] J. Veenstra‐VanderWeele , G. M. Anderson , and E. H. Cook Jr. , “Pharmacogenetics and the Serotonin System: Initial Studies and Future Directions,” European Journal of Pharmacology 410, no. 2‐3 (2000): 165–181. 11134668 10.1016/s0014-2999(00)00814-1

[2] B. Aryal , T. Shimizu , J. Kadono , et al., “Post‐Resection Exhaustion of Intra‐Platelet Serotonin: Also an Indicator of Early Hepatocellular Carcinoma Recurrence?” Journal of Cancer 8, no. 19 (2017): 3984–3991. 29187873 10.7150/jca.20971 PMC5706000

[3] R. G. Ruddell , D. A. Mann , and G. A. Ramm , “The Function of Serotonin Within the Liver,” Journal of Hepatology 48, no. 4 (2008): 666–675. 18280000 10.1016/j.jhep.2008.01.006

[4] S. N. Young , “How to Increase Serotonin in the human Brain Without Drugs,” Journal of Psychiatry & Neuroscience 32 (2007): 394–399. 18043762 PMC2077351

[5] A. P. Kerckhoffs , J. J. ter Linde , L. M. Akkermans , and M. Samsom , “SERT and TPH‐1 mRNA Expression Are Reduced in Irritable Bowel Syndrome Patients Regardless of Visceral Sensitivity state in Large Intestine,” American Journal of Physiology Gastrointestinal and Liver Physiology 302, no. 9 (2012): G1053–G1060. 22323131 10.1152/ajpgi.00153.2011

[6] B. Pernow and J. Waldenstrom , “Paroxysmal Flushing and Other Symptoms Caused by 5‐hydroxytryptamine and Histamine in Patients With Malignant Tumours,” Lancet 267, no. 6845 (1954): 951. 13213086 10.1016/s0140-6736(54)92559-3

[7] W. G. Rice and J. Mitchener , “Histochemical Evidence of 5‐hydroxytryptamine in a Dog Mast Cell Tumour,” Nature 189 (1961): 767–768. 13741241 10.1038/189767a0

[8] R. C. Cîmpeanu , M. V. Boldeanu , R. V. Ahrițculesei , et al., “Correlation Between Neurotransmitters (Dopamine, Epinephrine, Norepinephrine, Serotonin), Prognostic Nutritional Index, Glasgow Prognostic Score, Systemic Inflammatory Response Markers, and TNM Staging in a Cohort of Colorectal Neuroendocrine Tumor Patients,” International Journal of Molecular Sciences 25, no. 13 (2024): 6977. 39000088 10.3390/ijms25136977 PMC11241815

[9] R. Arreola , E. Becerril‐Villanueva , and C. Cruz‐Fuentes , “Immunomodulatory Effects Mediated by Serotonin,” Journal of Immunology Research 2015 (2015): 354957. 25961058 10.1155/2015/354957 PMC4417587

[10] M. Lesurtel , C. Soll , R. Graf , and P. A. Clavien , “Role of Serotonin in the Hepato‐gastroIntestinal Tract: An Old Molecule for New Perspectives,” Cellular and Molecular Life Sciences 65, no. 6 (2008): 940–952. 18080089 10.1007/s00018-007-7377-3 PMC11131662

[11] F. G. Boess and I. L. Martin , “Molecular Biology of 5‐HT Receptors,” Neuropharmacology 33, no. 3‐4 (1994): 275–317. 7984267 10.1016/0028-3908(94)90059-0

[12] G. Richter , F. Stöckmann , J. M. Conlon , and W. Creutzfeldt , “Serotonin Release Into Blood After Food and Pentagastrin. Studies in Healthy Subjects and in Patients With Metastatic Carcinoid Tumors,” Gastroenterology 91, no. 3 (1986): 612–618. 2426155 10.1016/0016-5085(86)90630-x

[13] M. Dioguardi Burgio , J. Cros , and N. Panvini , “Serotonin Immunoreactive Pancreatic Neuroendocrine Neoplasm Associated With Main Pancreatic Duct Dilation: A Recognizable Entity With Excellent Long‐term Outcome,” European Radiology 31, no. 11 (2021): 8671–8681. 33977308 10.1007/s00330-021-08007-4

[14] H. Tanaka , T. Sawano , N. Konishi , et al., “Serotonin Induces Arcadlin in Hippocampal Neurons,” Neuroscience Letters 721 (2020): 134783. 31981722 10.1016/j.neulet.2020.134783

[15] M. Pourhamzeh , F. G. Moravej , M. Arabi , et al., “The Roles of Serotonin in Neuropsychiatric Disorders,” Cellular and Molecular Neurobiology 42, no. 6 (2022): 1671–1692. 33651238 10.1007/s10571-021-01064-9 PMC11421740

[16] S. W. Watts , S. F. Morrison , R. P. Davis , and S. M. Barman , “Serotonin and Blood Pressure Regulation,” Pharmacological Reviews 64, no. 2 (2012): 359–388. 22407614 10.1124/pr.111.004697 PMC3310484

[17] F. X. Chen , X. S. Chen , J. C. Guo , B. A. Zheng , and M. Guo , “Serotonin Transporter‐linked Polymorphic Region Genotypes in Relation to Stress Conditions Among Patients With Papillary Thyroid Carcinoma,” International Journal of Clinical and Experimental Pathology 12, no. 3 (2019): 968–977. 31933907 PMC6945171

[18] M. Zopun , B. Lieder , A. K. Holik , J. P. Ley , J. Hans , and V. Somoza , “Noncaloric Sweeteners Induce Peripheral Serotonin Secretion via the T1R3‐Dependent Pathway in Human Gastric Parietal Tumor Cells (HGT‐1),” Journal of Agricultural and Food Chemistry 66, no. 27 (2018): 7044–7053. 29874909 10.1021/acs.jafc.8b02071

[19] Q. Pang , C. Liu , J. Y. Zhang , et al., “Serotonin in Liver Tumor: Friend or Foe?” Hepatology 62, no. 1 (2015): 319. 10.1002/hep.27568 25330322

[20] D. Sarrouilhe , J. Clarhaut , N. Defamie , and M. Mesnil , “Serotonin and Cancer: What Is the Link?” Current Molecular Medicine 15, no. 1 (2015): 62–77. 25601469 10.2174/1566524015666150114113411

[21] D. Sarrouilhe and M. Mesnil , “Serotonin and human Cancer: A Critical View,” Biochimie 161 (2019): 46–50. 29936294 10.1016/j.biochi.2018.06.016

[22] P. Balakrishna , S. George , H. Hatoum , and S. Mukherjee , “Serotonin Pathway in Cancer,” International Journal of Molecular Sciences 22, no. 3 (2021): 1268. 33525332 10.3390/ijms22031268 PMC7865972

[23] W. T. Zandee , R. C. van Adrichem , K. Kamp , R. A. Feelders , M. F. van Velthuysen , and W. W. de Herder , “Incidence and Prognostic Value of Serotonin Secretion in Pancreatic Neuroendocrine Tumours,” Clinical Endocrinology 87, no. 2 (2017): 165–170. 28464233 10.1111/cen.13364

[24] C. G. Nebigil , J. M. Launay , P. Hickel , C. Tournois , and L. Maroteaux , “5‐hydroxytryptamine 2B Receptor Regulates Cell‐cycle Progression: Cross‐talk With Tyrosine Kinase Pathways,” PNAS 97, no. 6 (2000): 2591–2596. 10688905 10.1073/pnas.050282397 PMC15973

[25] M. E. Maffei , “5‐Hydroxytryptophan (5‐HTP): Natural Occurrence, Analysis, Biosynthesis, Biotechnology, Physiology and Toxicology,” International Journal of Molecular Sciences 22, no. 1 (2020): 181. 33375373 10.3390/ijms22010181 PMC7796270

[26] F. Cote , E. Thevenot , C. Fligny , et al., “Disruption of the Nonneuronal tph1 Gene Demonstrates the Importance of Peripheral Serotonin in Cardiac Function,” PNAS 100, no. 23 (2003): 13525–13530. 14597720 10.1073/pnas.2233056100 PMC263847

[27] Q. Q. Liu , X. X. Yao , S. H. Gao , et al., “Role of 5‐HT Receptors in Neuropathic Pain: Potential Therapeutic Implications,” Pharmacological Research 159 (2020): 104949. 32464329 10.1016/j.phrs.2020.104949

[28] A. Haduch , E. Bromek , W. Kuban , and W. A. Daniel , “The Engagement of Cytochrome P450 Enzymes in Tryptophan Metabolism,” Metabolites 13, no. 5 (2023): 629. 37233670 10.3390/metabo13050629 PMC10224044

[29] J. S. Bhullar , J. M. Leung , and M. S. Almehthel , “Management of Refractory Hypoglycaemia in a Metastatic Neuroendocrine Tumour co‐secreting Serotonin and Insulin,” BMJ Case Reports 13, no. 11 (2020): e236659. 10.1136/bcr-2020-236659 PMC7654127 33168531

[30] L. Yu , S. Li , J. Wei , H. Sun , C. Yang , and H. Tan , “Association of Serotonin Transporter‐linked Polymorphic Region (5‐HTTLPR) With Heat Pain Stimulation and Postoperative Pain in Gastric Cancer Patients,” Molecular Pain 17 (2021): 17448069211006606. 33882731 10.1177/17448069211006606 PMC8071976

[31] K. Hussaarts , F. A. Berger , L. Binkhorst , et al., “The Risk of QTc‐Interval Prolongation in Breast Cancer Patients Treated With Tamoxifen in Combination With Serotonin Reuptake Inhibitors,” Pharmaceutical Research 37, no. 1 (2019): 7. 31845095 10.1007/s11095-019-2746-9 PMC6914733

[32] T. Shinka , D. Onodera , T. Tanaka , et al., “Serotonin Synthesis and Metabolism‐related Molecules in a human Prostate Cancer Cell Line,” Oncology Letters 2, no. 2 (2011): 211–215. 22866066 10.3892/ol.2011.244 PMC3410569

[33] L. F. Mohammad‐Zadeh , L. Moses , and S. M. Gwaltney‐Brant , “Serotonin: A Review,” Journal of Veterinary Pharmacology and Therapeutics 31, no. 3 (2008): 187–199. 18471139 10.1111/j.1365-2885.2008.00944.x

[34] M. D. Ferrari , J. Odink , C. Tapparelli , G. M. Van Kempen , E. J. Pennings , and G. W. Bruyn , “Serotonin Metabolism in Migraine,” Neurology 39, no. 9 (1989): 1239–1242. 2475821 10.1212/wnl.39.9.1239

[35] M. B. H. Youdim and M. Weinstock , “Therapeutic Applications of Selective and Non‐Selective Inhibitors of Monoamine Oxidase A and B That Do Not Cause Significant Tyramine Potentiation,” Neurotoxicology 25, no. 1 (2004): 243–250. 14697899 10.1016/S0161-813X(03)00103-7

[36] H. Morita , E. Mochiki , N. Takahashi , et al., “Effects of 5‐HT2B, 5‐HT3 and 5‐HT4 Receptor Antagonists on Gastrointestinal Motor Activity in Dogs,” World Journal of Gastroenterology 19, no. 39 (2013): 6604–6612. 24151388 10.3748/wjg.v19.i39.6604 PMC3801375

[37] D. G. Grahame‐Smith , “Serotonin (5‐hydroxytryptamine, 5‐HT),” Quarterly Journal of Medicine 67, no. 254 (1988): 459–466. 3074334

[38] P. G. McLean , R. A. Borman , and K. Lee , “5‐HT in the Enteric Nervous System: Gut Function and Neuropharmacology,” Trends in Neuroscience (Tins) 30, no. 1 (2007): 9–13. 17126921 10.1016/j.tins.2006.11.002

[39] A. Meneses , “Physiological, Pathophysiological and Therapeutic Roles of 5‐HT Systems in Learning and Memory,” Reviews in the Neurosciences 9, no. 4 (1998): 275–289. 9886142 10.1515/revneuro.1998.9.4.275

[40] A. R. Green , “Neuropharmacology of 5‐hydroxytryptamine,” British Journal of Pharmacology 147, no. Suppl 1 (2006): S145–S152. 16402098 10.1038/sj.bjp.0706427 PMC1760750

[41] S. M. Stahl , C. Lee‐Zimmerman , S. Cartwright , and D. A. Morrissette , “Serotonergic Drugs for Depression and Beyond,” Current Drug Targets 14, no. 5 (2013): 578–585. 23531115 10.2174/1389450111314050007

[42] H. Zhang , H. Xu , Q. Tang , and F. Bi , “The Selective Serotonin Reuptake Inhibitors Enhance the Cytotoxicity of sorafenib in Hepatocellular Carcinoma Cells,” Anti‐Cancer Drugs 32, no. 8 (2021): 793–801. 33675613 10.1097/CAD.0000000000001067

[43] L. Bardin , “The Complex Role of Serotonin and 5‐HT Receptors in Chronic Pain,” Behavioural Pharmacology 22, no. 5‐6 (2011): 390–404. 21808193 10.1097/FBP.0b013e328349aae4

[44] C. Sommer , “Serotonin in Pain and Analgesia: Actions in the Periphery,” Molecular Neurobiology 30, no. 2 (2004): 117–125. 15475622 10.1385/MN:30:2:117

[45] C. Sommer , “Is Serotonin Hyperalgesic or Analgesic?” Current Pain and Headache Reports 10, no. 2 (2006): 101–106. 16539862 10.1007/s11916-006-0020-4

[46] R. Suzuki , L. J. Rygh , and A. H. Dickenson , “Bad News From the Brain: Descending 5‐HT Pathways That Control Spinal Pain Processing,” Trends in Pharmacological Sciences 25, no. 12 (2004): 613–617. 15530638 10.1016/j.tips.2004.10.002

[47] D. Armstrong , R. M. Dry , C. A. Keele , and J. W. Markham , “Pain‐producing Actions of Tryptamine and 5‐hydroxytryptamine,” The Journal of Physiology 117, no. 4 (1952): 70p–71p. 12991261

[48] M. Fava , “The Role of the Serotonergic and Noradrenergic Neurotransmitter Systems in the Treatment of Psychological and Physical Symptoms of Depression,” Journal of Clinical Psychiatry 64, no. Suppl 13 (2003): 26–29. 14552653

[49] K. Z. Peters , J. F. Cheer , and R. Tonini , “Modulating the Neuromodulators: Dopamine, Serotonin, and the Endocannabinoid System,” Trends in Neuroscience (Tins) 44, no. 6 (2021): 464–477. 33674134 10.1016/j.tins.2021.02.001 PMC8159866

[50] R. Yamamoto , T. Ito , T. Furuyama , M. Ono , and N. Kato , “5‐HT and α‐m‐5‐HT Attenuate Excitatory Synaptic Transmissions Onto the Lateral Amygdala Principal Neurons via Presynaptic 5‐HT(1B) Receptors,” Biochemical and Biophysical Research Communications 624 (2022): 28–34. 35932576 10.1016/j.bbrc.2022.07.076

[51] S. Afshar , S. Shahidi , H. Baooshi , et al., “The Role of Hippocampal 5‐HT(1D) and 5‐HT(1F) Receptors on Learning and Memory in Rats,” Naunyn‐Schmiedebergs Archives of Pharmacology 396, no. 7 (2023): 1451–1460. 36749399 10.1007/s00210-023-02411-x

[52] J. Zeng , X. Li , R. Zhang , et al., “Local 5‐HT Signaling bi‐directionally Regulates the Coincidence Time Window for Associative Learning,” Neuron 111, no. 7 (2023): 1118–1135. e5. 36706757 10.1016/j.neuron.2022.12.034 PMC11152601

[53] M. J. Robson , M. A. Quinlan , and R. D. Blakely , “Immune System Activation and Depression: Roles of Serotonin in the Central Nervous System and Periphery,” Acs Chemical Neuroscience 8, no. 5 (2017): 932–942. 28345868 10.1021/acschemneuro.6b00412

[54] N. Israelyan , A. Del Colle , Z. Li , et al., “Effects of Serotonin and Slow‐Release 5‐Hydroxytryptophan on Gastrointestinal Motility in a Mouse Model of Depression,” Gastroenterology 157, no. 2 (2019): 507–521. e4. 31071306 10.1053/j.gastro.2019.04.022 PMC6650329

[55] Z. Chen , J. Luo , J. Li , et al., “Interleukin‐33 Promotes Serotonin Release From Enterochromaffin Cells for Intestinal Homeostasis,” Immunity 54, no. 1 (2021): 151–163. e6. 33220232 10.1016/j.immuni.2020.10.014 PMC7856083

[56] N. J. Spencer and D. J. Keating , “Is There a Role for Endogenous 5‐HT in Gastrointestinal Motility? How Recent Studies Have Changed Our Understanding,” Advances in Experimental Medicine and Biology 891 (2016): 113–122. 27379639 10.1007/978-3-319-27592-5_11

[57] M. Yang , H. Fukui , H. Eda , et al., “Involvement of Gut Microbiota in the Association Between Gastrointestinal Motility and 5‑HT Expression/M2 Macrophage Abundance in the Gastrointestinal Tract,” Molecular Medicine Reports 16, no. 3 (2017): 3482–3488. 28714029 10.3892/mmr.2017.6955

[58] I. Hanna‐Jairala and D. A. Drossman , “Central Neuromodulators in Irritable Bowel Syndrome: Why, How, and When,” American Journal of Gastroenterology 119, no. 7 (2024): 1272–1284. 38595149 10.14309/ajg.0000000000002800 PMC11208063

[59] J. Bai , Y. Cai , Z. Huang , et al., “Shouhui Tongbian Capsule Ameliorates Constipation via Gut Microbiota‐5‐HT‐intestinal Motility Axis,” Biomedicine & Pharmacotherapy 154 (2022): 113627. 36058152 10.1016/j.biopha.2022.113627

[60] K. Merecz , M. Hirsa , O. Biniszewska , J. Fichna , and A. Tarasiuk , “An Overview of 5‐HT(3) Receptor Antagonists as a Treatment Option for Irritable Bowel Syndrome With Diarrhea,” Expert Opinion on Pharmacotherapy 24, no. 10 (2023): 1189–1198. 37173833 10.1080/14656566.2023.2214314

[61] C. Stasi , S. Sadalla , and S. Milani , “The Relationship between the Serotonin Metabolism, Gut‐Microbiota and the Gut‐Brain Axis,” Current Drug Metabolism 20, no. 8 (2019): 646–655. 31345143 10.2174/1389200220666190725115503

[62] Y. H. Kwon and W. I. Khan , “Peripheral Serotonin: Cultivating Companionship With Gut Microbiota in Intestinal Homeostasis,” American Journal of Physiology. Cell Physiology 323, no. 2 (2022): C550–C555. 35759441 10.1152/ajpcell.00433.2021

[63] E. Ayme‐Dietrich , G. Aubertin‐Kirch , L. Maroteaux , and L. Monassier , “Cardiovascular Remodeling and the Peripheral Serotonergic System,” Archives of Cardiovascular Diseases 110, no. 1 (2017): 51–59. 28017279 10.1016/j.acvd.2016.08.002

[64] S. A. Doggrell , “The Role of 5‐HT on the Cardiovascular and Renal Systems and the Clinical Potential of 5‐HT Modulation,” Expert Opinion on Investigational Drugs 12, no. 5 (2003): 805–823. 12720492 10.1517/13543784.12.5.805

[65] N. Kajiwara , T. Kushiro , and H. Hayashi , “[Blood pressure regulation and serotonin],” Nihon Rinsho 43, no. 5 (1985): 923–928. 3897649

[66] C. Schoenichen , C. Bode , and D. Duerschmied , “Role of Platelet Serotonin in Innate Immune Cell Recruitment,” Frontiers in Bioscience‐Landmark 24, no. 3 (2019): 514–526. 10.2741/4732 30468670

[67] K. Nishihira , A. Yamashita , N. Tanaka , et al., “Inhibition of 5‐hydroxytryptamine Receptor Prevents Occlusive Thrombus Formation on Neointima of the Rabbit Femoral Artery,” Journal of Thrombosis and Haemostasis 4, no. 1 (2006): 247–255. 16409475 10.1111/j.1538-7836.2005.01702.x

[68] A. M. Galan , I. Lopez‐Vilchez , M. Diaz‐Ricart , et al., “Serotonergic Mechanisms Enhance Platelet‐mediated Thrombogenicity,” Thromb Haemost 102, no. 3 (2009): 511–519. 19718472 10.1160/TH08-12-0810

[69] N. Liu , S. Sun , P. Wang , Y. Sun , Q. Hu , and X. Wang , “The Mechanism of Secretion and Metabolism of Gut‐Derived 5‐Hydroxytryptamine,” International Journal of Molecular Sciences 22, no. 15 (2021): 7931. 34360695 10.3390/ijms22157931 PMC8347425

[70] Y. Wu , J. Ma , J. Chen , et al., “Ablation of CD44 Attenuates Adipogenesis in White Adipocytes via the Tryptophan 5‐Hydroxylase 2/5‐Hydroxytryptamine Axis to Protect Mice From High‐Fat Diet‐Induced Obesity,” American Journal of Pathology 195, no. 2 (2025): 247–264. 39476955 10.1016/j.ajpath.2024.10.005

[71] H. Wu , T. H. Denna , J. N. Storkersen , and V. A. Gerriets , “Beyond a Neurotransmitter: The Role of Serotonin in Inflammation and Immunity,” Pharmacological Research 140 (2019): 100–114. 29953943 10.1016/j.phrs.2018.06.015

[72] A. Parajulee and K. Kim , “Structural Studies of Serotonin Receptor family,” BMB Reports 56, no. 10 (2023): 527–536. 37817438 10.5483/BMBRep.2023-0147 PMC10618075

[73] T. Sharp and N. M. Barnes , “Central 5‐HT Receptors and Their Function; Present and Future,” Neuropharmacology 177 (2020): 108155. 32522572 10.1016/j.neuropharm.2020.108155

[74] N. I. Kalinina , A. V. Zaitsev , and N. P. Vesselkin , “Presynaptic Serotonin 5‐HT(1B/D) Receptor‐mediated Inhibition of Glycinergic Transmission to the Frog Spinal Motoneurons,” Journal of Comparative Physiology. A, Neuroethology, Sensory, Neural, and Behavioral Physiology 204, no. 3 (2018): 329–337. 29290056 10.1007/s00359-017-1244-y

[75] V. K. Sharma and Y. P. Loh , “The Discovery, Structure, and Function of 5‐HTR1E Serotonin Receptor,” Cell Communication and Signaling 21, no. 1 (2023): 235. 37723479 10.1186/s12964-023-01195-0 PMC10506339

[76] J. Yu , Z. Wang , Y. Chen , and Y. Dong , “Restraint Stress Disrupted Intestinal Homeostasis via 5‐HT/HTR7/Wnt/β‐Catenin/NF‐kB Signaling,” International Journal of Molecular Sciences 26, no. 9 (2025): 4021. 40362261 10.3390/ijms26094021 PMC12071331

[77] I. Moutkine , E. L. Collins , C. Béchade , and L. Maroteaux , “Evolutionary Considerations on 5‐HT(2) Receptors,” Pharmacological Research 140 (2019): 14–20. 30223085 10.1016/j.phrs.2018.09.014

[78] S. Chaumont‐Dubel , V. Dupuy , J. Bockaert , C. Bécamel , and P. Marin , “The 5‐HT(6) Receptor Interactome: New Insight in Receptor Signaling and Its Impact on Brain Physiology and Pathologies,” Neuropharmacology 172 (2020): 107839. 31682856 10.1016/j.neuropharm.2019.107839

[79] A. Chagraoui , F. Thibaut , and P. De Deurwaerdère , “5‐HT6 receptors: Contemporary Views on Their Neurobiological and Pharmacological Relevance in Neuropsychiatric Disorders,” Dialogues in Clinical Neuroscience 27, no. 1 (2025): 112–128. 40347153 10.1080/19585969.2025.2502028 PMC12068339

[80] A. Åstrand , D. Guerrieri , S. Vikingsson , R. Kronstrand , and H. Green , “In Vitro Characterization of New Psychoactive Substances at the μ‐opioid, CB1, 5HT(1A), and 5‐HT(2A) Receptors‐On‐target Receptor Potency and Efficacy, and off‐target Effects,” Forensic Science International 317 (2020): 110553. 33160102 10.1016/j.forsciint.2020.110553

[81] A. Tadjalli and G. S. Mitchell , “Cervical Spinal 5‐HT(2A) and 5‐HT(2B) Receptors Are Both Necessary for Moderate Acute Intermittent Hypoxia‐induced Phrenic Long‐term Facilitation,” Journal of Applied Physiology 127, no. 2 (2019): 432–443. 31219768 10.1152/japplphysiol.01113.2018 PMC6732436

[82] P. Campos‐Bedolla , E. G. Torrejón‐González , D. Mendoza‐Mejía , et al., “Role of 5‐HT2 Receptors family in the Allergy‐induced Increased Aorta Contractile Responses to 5‐HT,” Physiological Research 72, no. 1 (2023): 111–116. 36545875 10.33549/physiolres.934968 PMC10069811

[83] G. Fakhfouri , K. Mousavizadeh , S. E. Mehr , et al., “From Chemotherapy‐Induced Emesis to Neuroprotection: Therapeutic Opportunities for 5‐HT3 Receptor Antagonists,” Molecular Neurobiology 52, no. 3 (2015): 1670–1679. 25377794 10.1007/s12035-014-8957-5

[84] Q. Cheng , X. Feng , Q. Meng , et al., “[6]‐** *Gingerol Ameliorates Cisplatin‐Induced Pica by Regulating the* ** TPH/MAO‐A/SERT/5‐HT/5‐HT(3) Receptor System in Rats,” Drug Design, Development and Therapy 14 (2020): 4085–4099. 33061309 10.2147/DDDT.S270185 PMC7538004

[85] J. B. Pineda‐Farias , P. Barragán‐Iglesias , A. Valdivieso‐Sánchez , et al., “Spinal 5‐HT(4) and 5‐HT(6) Receptors Contribute to the Maintenance of Neuropathic Pain in Rats,” Pharmacology Reports 69, no. 5 (2017): 916–923. 10.1016/j.pharep.2017.04.001 28628851

[86] A. Petelák , N. A. Lambert , and A. Bondar , “Serotonin 5‐HT(7) Receptor Slows Down the G(s) Protein: A Single Molecule Perspective,” Molecular Biology of the Cell 34, no. 9 (2023): br14. 37342875 10.1091/mbc.E23-03-0117 PMC10398887

[87] Y. Zhou , J. Ma , X. Lin , X. P. Huang , K. Wu , and N. Huang , “Structure‐Based Discovery of Novel and Selective 5‐Hydroxytryptamine 2B Receptor Antagonists for the Treatment of Irritable Bowel Syndrome,” Journal of Medicinal Chemistry 59, no. 2 (2016): 707–720. 26700945 10.1021/acs.jmedchem.5b01631

[88] B. Mao , S. Liu , S. Zhu , et al., “The janus Face of Serotonin: Regenerative Promoter and Chronic Liver Disease Aggravator,” Heliyon 10, no. 9 (2024): e30703. 38756588 10.1016/j.heliyon.2024.e30703 PMC11096747

[89] R. E. Tay , C. M. Ho , N. D. Z. Ang , et al., “Serotonin Receptor 5‐HT(2A) as a Potential Target for HCC Immunotherapy,” Journal for ImmunoTherapy of Cancer 13, no. 6 (2025): e011088. 40550560 10.1136/jitc-2024-011088 PMC12186046

[90] D. Hoyer , “5‐HT Receptor Nomenclature: Naming Names, Does It Matter? A Tribute to Maurice Rapport,” Acs Chemical Neuroscience 8, no. 5 (2017): 908–919. 28269984 10.1021/acschemneuro.7b00011

[91] O. E. Brodde , “5‐Hydroxytryptamine‐receptor Subtypes,” Clinical Physiology and Biochemistry 8, no. Suppl 3 (1990): 19–27. 2132174

[92] J. F. López‐Giménez and J. González‐Maeso , “Hallucinogens and Serotonin 5‐HT(2A) Receptor‐Mediated Signaling Pathways,” Current Topics in Behavioral Neurosciences 36 (2018): 45–73. 28677096 10.1007/7854_2017_478 PMC5756147

[93] K. Kim , T. Che , O. Panova , et al., “Structure of a Hallucinogen‐Activated Gq‐Coupled 5‐HT(2A) Serotonin Receptor,” Cell 182, no. 6 (2020): 1574–1588. e19. 32946782 10.1016/j.cell.2020.08.024 PMC7593816

[94] M. B. Zimering , “Severe COVID‐19 Pneumonia Is Associated With Increased Plasma Immunoglobulin G Agonist Autoantibodies Targeting the 5‐Hydroxytryptamine 2A Receptor,” Endocrinology, Diabetes & Metabolism 5, no. 1 (2021): 1–9. 10.31038/EDMJ.2021511 PMC7931266 33680365

[95] D. O. Borroto‐Escuela , X. Li , A. O. Tarakanov , et al., “Existence of Brain 5‐HT1A‐5‐HT2A Isoreceptor Complexes With Antagonistic Allosteric Receptor‐Receptor Interactions Regulating 5‐HT1A Receptor Recognition,” ACS Omega 2, no. 8 (2017): 4779–4789. 28920103 10.1021/acsomega.7b00629 PMC5597955

[96] Z. Barclay , L. Dickson , D. Robertson , et al., “Attenuated PLD1 Association and Signalling at the H452Y Polymorphic Form of the 5‐HT(2A) Receptor,” Cell Signalling 25, no. 4 (2013): 814–821. 23314176 10.1016/j.cellsig.2013.01.004

[97] A. Iglesias , M. Cimadevila , M. I. Cadavid , M. I. Loza , and J. Brea , “Serotonin‐2A Homodimers Are Needed for Signalling via both Phospholipase A(2) and Phospholipase C in Transfected CHO Cells,” European Journal of Pharmacology 800 (2017): 63–69. 28216047 10.1016/j.ejphar.2017.02.028

[98] Z. Barclay , L. Dickson , D. N. Robertson , et al., “5‐HT2A receptor Signalling Through Phospholipase D1 Associated With Its C‐terminal Tail,” Biochemical Journal 436, no. 3 (2011): 651–660. 21410433 10.1042/BJ20101844

[99] L. A. Desouza , M. Benekareddy , S. E. Fanibunda , et al., “The Hallucinogenic Serotonin(2A) Receptor Agonist, 2,5‐Dimethoxy‐4‐Iodoamphetamine, Promotes cAMP Response Element Binding Protein‐Dependent Gene Expression of Specific Plasticity‐Associated Genes in the Rodent Neocortex,” Frontiers in Molecular Neuroscience 14 (2021): 790213. 35002622 10.3389/fnmol.2021.790213 PMC8739224

[100] H. Zhu , X. Liu , X. Wang , et al., “Gβγ Subunit Inhibitor Decreases DOM‐induced Head Twitch Response via the PLCβ/IP3/Ca(2+)/ERK and cAMP Signaling Pathways,” European Journal of Pharmacology 957 (2023): 176038. 37657742 10.1016/j.ejphar.2023.176038

[101] F. Borsini , E. Giraldo , and E. Monferini , “BIMT 17, a 5‐HT2A Receptor Antagonist and 5‐HT1A Receptor Full Agonist in Rat Cerebral Cortex,” Naunyn‐Schmiedebergs Archives of Pharmacology 352, no. 3 (1995): 276–282. 8584042 10.1007/BF00168557

[102] D. M. El‐Tanbouly , W. Wadie , and R. H. Sayed , “Modulation of TGF‐β/Smad and ERK Signaling Pathways Mediates the Anti‐fibrotic Effect of Mirtazapine in Mice,” Toxicology and Applied Pharmacology 329 (2017): 224–230. 28623179 10.1016/j.taap.2017.06.012

[103] Z. Marinova , S. Walitza , and E. Grünblatt , “5‐HT2A serotonin Receptor Agonist DOI Alleviates Cytotoxicity in Neuroblastoma Cells: Role of the ERK Pathway,” Progress in Neuro‐Psychopharmacology & Biological Psychiatry 44 (2013): 64–72. 23380172 10.1016/j.pnpbp.2013.01.017

[104] J. A. Florian and S. W. Watts , “Integration of Mitogen‐activated Protein Kinase Kinase Activation in Vascular 5‐hydroxytryptamine2A Receptor Signal Transduction,” Journal of Pharmacology and Experimental Therapeutics 284, no. 1 (1998): 346–355. 9435197

[105] M. Göőz , P. Göőz , L. M. Luttrell , and J. R. Raymond , “5‐HT2A receptor Induces ERK Phosphorylation and Proliferation Through ADAM‐17 Tumor Necrosis Factor‐alpha‐converting Enzyme (TACE) Activation and Heparin‐bound Epidermal Growth Factor‐Like Growth Factor (HB‐EGF) Shedding in Mesangial Cells,” Journal of Biological Chemistry 281, no. 30 (2006): 21004–21012. 16737974 10.1074/jbc.M512096200

[106] S. W. Watts , “Activation of the Mitogen‐activated Protein Kinase Pathway via the 5‐HT2A Receptor,” Annals of the New York Academy of Sciences 861 (1998): 162–168. 9928253 10.1111/j.1749-6632.1998.tb10187.x

[107] P. V. Avdonin , A. D. Nadeev , G. Y. Mironova , I. L. Zharkikh , P. P. Avdonin , and N. V. Goncharov , “Enhancement by Hydrogen Peroxide of Calcium Signals in Endothelial Cells Induced by 5‐HT1B and 5‐HT2B Receptor Agonists,” Oxidative Medicine and Cellular Longevity 2019 (2019): 1701478. 30886671 10.1155/2019/1701478 PMC6388333

[108] T. Oufkir , M. Arseneault , J. T. Sanderson , and C. Vaillancourt , “The 5‐HT 2A Serotonin Receptor Enhances Cell Viability, Affects Cell Cycle Progression and Activates MEK‐ERK1/2 and JAK2‐STAT3 Signalling Pathways in human Choriocarcinoma Cell Lines,” Placenta 31, no. 5 (2010): 439–447. 20338635 10.1016/j.placenta.2010.02.019

[109] T. Klempan , A. A. Hudon‐Thibeault , T. Oufkir , C. Vaillancourt , and J. T. Sanderson , “Stimulation of Serotonergic 5‐HT2A Receptor Signaling Increases Placental Aromatase (CYP19) Activity and Expression in BeWo and JEG‐3 human Choriocarcinoma Cells,” Placenta 32, no. 9 (2011): 651–656. 21703684 10.1016/j.placenta.2011.06.003

[110] M. I. Ahmed , H. M. A. Abdelrazek , Y. M. Moustafa , et al., “Cardioprotective Effect of Flibanserin Against Isoproterenol‐Induced Myocardial Infarction in Female Rats: Role of Cardiac 5‐HT2A Receptor Gene/5‐HT/Ca(2+) Pathway,” Pharmaceuticals (Basel) 16, no. 4 (2023): 502. 37111259 10.3390/ph16040502 PMC10143970

[111] Y. Kanda , M. Okada , R. Ikarashi , E. Morioka , T. Kondo , and M. Ikeda , “Bimodal Modulation of Store‐operated Ca(2+) Channels by Clozapine in Astrocytes,” Neuroscience Letters 635 (2016): 56–60. 27769892 10.1016/j.neulet.2016.10.027

[112] I. P. Voronova , G. M. Khramova , E. A. Kulikova , D. V. Petrovskii , D. V. Bazovkina , and A. V. Kulikov , “5‐HT2A receptors Control Body Temperature in Mice During LPS‐induced Inflammation via Regulation of NO Production,” Pharmacological Research 103 (2016): 123–131. 26621247 10.1016/j.phrs.2015.11.018

[113] K. Padhariya , R. Bhandare , D. Canney , and V. Velingkar , “Cardiovascular Concern of 5‐HT2B Receptor and Recent Vistas in the Development of Its Antagonists,” Cardiovascular & Hematological Disorders‐Drug Targets 17, no. 2 (2017): 86–104. 28676029 10.2174/1871529X17666170703115111

[114] W. Janssen , Y. Schymura , T. Novoyatleva , et al., “5‐HT2B receptor Antagonists Inhibit Fibrosis and Protect From RV Heart Failure,” BioMed Research International 2015 (2015): 438403. 25667920 10.1155/2015/438403 PMC4312574

[115] S. Fatima , X. Shi , Z. Lin , et al., “5‐Hydroxytryptamine Promotes Hepatocellular Carcinoma Proliferation by Influencing β‐catenin,” Molecular Oncology 10, no. 2 (2016): 195–212. 26474915 10.1016/j.molonc.2015.09.008 PMC5528951

[116] J. M. Launay , G. Birraux , D. Bondoux , et al., “Ras Involvement in Signal Transduction by the Serotonin 5‐HT2B Receptor,” Journal of Biological Chemistry 271, no. 6 (1996): 3141–3147. 8621713 10.1074/jbc.271.6.3141

[117] N. M. Barnes , T. G. Hales , S. C. Lummis , and J. A. Peters , “The 5‐HT3 Receptor–the Relationship Between Structure and Function,” Neuropharmacology 56, no. 1 (2009): 273–284. 18761359 10.1016/j.neuropharm.2008.08.003 PMC6485434

[118] S. C. Lummis , “5‐HT(3) Receptors,” Journal of Biological Chemistry 287, no. 48 (2012): 40239–40245. 23038271 10.1074/jbc.R112.406496 PMC3504740

[119] A. J. Thompson and S. C. Lummis , “5‐HT3 receptors,” Current Pharmaceutical Design 12, no. 28 (2006): 3615–3630. 17073663 10.2174/138161206778522029 PMC2664614

[120] A. Barzegar‐Fallah , H. Alimoradi , J. L. Dunlop , E. Torbati , and S. K. Baird , “Serotonin Type‐3 Receptor Antagonists Selectively Kill Melanoma Cells Through Classical Apoptosis, Microtubule Depolymerisation, ERK Activation, and NF‐kappaB Downregulation,” Cell Biology and Toxicology (2021). 10.1007/s10565-021-09667-0 34654991

[121] S. M. El‐Khatib and A. M. Preston , “Effect of Palytoxin and Serotonin on Murine Tumor Cells,” JNCI: Journal of the National Cancer Institute 63, no. 1 (1979): 75–79. 286838

[122] D. R. Linden and E. E. El‐Fakahany , “Microglial Derived Nitric Oxide Decreases Serotonin Content in Rat Basophilic Leukemia (RBL‐2H3) Cells,” European Journal of Pharmacology 436, no. 1‐2 (2002): 53–56. 11834246 10.1016/s0014-2999(01)01615-6

[123] M. Tone , S. Tahara , S. Nojima , D. Motooka , D. Okuzaki , and E. Morii , “HTR3A is Correlated With Unfavorable Histology and Promotes Proliferation Through ERK Phosphorylation in Lung Adenocarcinoma,” Cancer Science 111, no. 10 (2020): 3953–3961. 32736413 10.1111/cas.14592 PMC7540989

[124] L. Lanfumey and M. Hamon , “5‐HT1 receptors,” Current Drug Targets. CNS and Neurological Disorders 3, no. 1 (2004): 1–10. 14965240 10.2174/1568007043482570

[125] P. Xu , S. Huang , H. Zhang , et al., “Structural Insights Into the Lipid and Ligand Regulation of Serotonin Receptors,” Nature 592, no. 7854 (2021): 469–473. 33762731 10.1038/s41586-021-03376-8

[126] G. Y. Tang , R. J. Wang , Y. Guo , and J. Liu , “5‐HT(1B) Receptor‐AC‐PKA Signal Pathway in the Lateral Habenula Is Involved in the Regulation of Depressive‐Like Behaviors in 6‐hydroxydopamine‐induced Parkinson's Rats,” Neurological Research 45, no. 2 (2023): 127–137. 36127643 10.1080/01616412.2022.2124797

[127] P. R. Albert and F. Vahid‐Ansari , “The 5‐HT1A Receptor: Signaling to Behavior,” Biochimie 161 (2019): 34–45. 31079617 10.1016/j.biochi.2018.10.015

[128] Y. G. Ni , M. M. Panicker , and R. Miledi , “Efficient Coupling of 5‐HT1a Receptors to the Phospholipase C Pathway in Xenopus Oocytes,” Brain Research Molecular Brain Research 51, no. 1‐2 (1997): 115–122. 9427513 10.1016/s0169-328x(97)00225-8

[129] K. A. Berg and W. P. Clarke , “Regulation of 5‐HT(1A) and 5‐HT(1B) Receptor Systems by Phospholipid Signaling Cascades,” Brain Research Bulletin 56, no. 5 (2001): 471–477. 11750792 10.1016/s0361-9230(01)00645-1

[130] M. Zhang , X. Qian , Z. Wei , et al., “Micro‐Infusion of 5‐HT1a Receptor Antagonists Into the Ventral Subiculum Ameliorate MK‐801 Induced Schizophrenia‐Like Behavior in Rats,” Neuroscience 552 (2024): 115–125. 38909674 10.1016/j.neuroscience.2024.06.010

[131] N. Gurbuz , M. R. Asoglu , A. A. Ashour , S. Salama , G. S. Kilic , and B. Ozpolat , “A Selective Serotonin 5‐HT(1B) Receptor Inhibition Suppresses Cells Proliferation and Induces Apoptosis in human Uterine Leiomyoma Cells,” European Journal of Obstetrics, Gynecology, and Reproductive Biology 206 (2016): 114–119. 27669395 10.1016/j.ejogrb.2016.08.006

[132] Y. Liu , Y. J. Suzuki , R. M. Day , and B L. Fanburg , “Rho Kinase‐induced Nuclear Translocation of ERK1/ERK2 in Smooth Muscle Cell Mitogenesis Caused by Serotonin,” Circulation Research 95, no. 6 (2004): 579–586. 15297378 10.1161/01.RES.0000141428.53262.a4

[133] Y. V. Mukhin , M. N. Garnovskaya , G. Collinsworth , et al., “5‐Hydroxytryptamine1A receptor/Gibetagamma Stimulates Mitogen‐activated Protein Kinase via NAD(P)H Oxidase and Reactive Oxygen Species Upstream of Src in chinese Hamster Ovary Fibroblasts,” Biochemical Journal 347, no. Pt 1 (2000): 61–67. 10727402 PMC1220931

[134] T. Machida , K. Iizuka , and M. Hirafuji , “5‐hydroxytryptamine and Its Receptors in Systemic Vascular Walls,” Biological & pharmaceutical bulletin 36, no. 9 (2013): 1416–1419. 23995652 10.1248/bpb.b13-00344

[135] S. Hemmati , N. Rahimi , S. Dabiri , M. Alaeddini , S. Etemad‐Moghadam , and A. R. Dehpour , “Inhibition of Ovalbumin‐induced Allergic Rhinitis by sumatriptan Through the Nitric Oxide Pathway in Mice,” Life Sciences 236 (2019): 116901. 31610206 10.1016/j.lfs.2019.116901

[136] M. Iwabayashi , Y. Taniyama , F. Sanada , et al., “Role of Serotonin in Angiogenesis: Induction of Angiogenesis by Sarpogrelate via Endothelial 5‐HT1B/Akt/eNOS Pathway in Diabetic Mice,” Atherosclerosis 220, no. 2 (2012): 337–342. 22172591 10.1016/j.atherosclerosis.2011.10.042

[137] J. J. Lee , E. T. Hahm , C. H. Lee , and Y. W. Cho , “5‐HT1A receptor‐mediated Activation of a G‐protein‐coupled Inwardly Rectifying K+ Current in Rat Medial Preoptic Area Neurons,” European Journal of Pharmacology 586, no. 1‐3 (2008): 114–122. 18367171 10.1016/j.ejphar.2008.02.051

[138] F. Amber‐Cicek , O. Ugur , K. Sayar , and M. Ugur , “Cell Adhesion Modulates 5‐HT(1D) and P2Y Receptor Signal Trafficking Differentially in LTK‐8 Cells,” European Journal of Pharmacology 590, no. 1‐3 (2008): 12–19. 18582865 10.1016/j.ejphar.2008.05.012

[139] D. N. Middlemiss , “The Putative 5‐HT1 Receptor Agonist, RU 24969, Inhibits the Efflux of 5‐hydroxytryptamine From Rat Frontal Cortex Slices by Stimulation of the 5‐HT Autoreceptor,” Journal of Pharmacy and Pharmacology 37, no. 6 (1985): 434–437. 2862270 10.1111/j.2042-7158.1985.tb03032.x

[140] X. Zhao , Y. Zhang , W. Qin , et al., “Serotonin Type‐1D Receptor Stimulation of A‐type K(+) Channel Decreases Membrane Excitability Through the Protein Kinase A‐ and B‐Raf‐dependent p38 MAPK Pathways in Mouse Trigeminal Ganglion Neurons,” Cell Signalling 28, no. 8 (2016): 979–988. 27156838 10.1016/j.cellsig.2016.05.004

[141] P. R. Debata , B. Ranasinghe , A. Berliner , et al., “Erk1/2‐dependent Phosphorylation of PKCalpha at Threonine 638 in Hippocampal 5‐HT(1A) Receptor‐mediated Signaling,” Biochemical and Biophysical Research Communications 397, no. 3 (2010): 401–406. 20513439 10.1016/j.bbrc.2010.05.096 PMC2921846

[142] A. M. Leone , M. Errico , S. L. Lin , and D. S. Cowen , “Activation of Extracellular Signal‐regulated Kinase (ERK) and Akt by human Serotonin 5‐HT(1B) Receptors in Transfected BE(2)‐C Neuroblastoma Cells Is Inhibited by RGS4,” Journal of Neurochemistry 75, no. 3 (2000): 934–938. 10936173 10.1046/j.1471-4159.2000.0750934.x

[143] M. Capi , V. De Angelis , D. De Bernardini , et al., “CGRP Receptor Antagonists and 5‐HT1F Receptor Agonist in the Treatment of Migraine,” Journal of Clinical Medicine 10, no. 7 (2021): 1429. 33916043 10.3390/jcm10071429 PMC8038117

[144] J. Bockaert , S. Claeysen , V. Compan , and A. Dumuis , “5‐HT4 receptors,” Current Drug Targets CNS and Neurological Disorders 3, no. 1 (2004): 39–51. 14965243 10.2174/1568007043482615

[145] S. S. Hegde and R. M. Eglen , “Peripheral 5‐HT4 Receptors,” Faseb Journal 10, no. 12 (1996): 1398–1407. 8903510 10.1096/fasebj.10.12.8903510

[146] S. Weninger , K. Van Craenenbroeck , R. T. Cameron , et al., “Phosphodiesterase 4 Interacts With the 5‐HT4(b) Receptor to Regulate cAMP Signaling,” Cell Signalling 26, no. 11 (2014): 2573–2582. 25101859 10.1016/j.cellsig.2014.07.027

[147] Z. Yu , L. Zikela , D. Wang , et al., “Effects and Mechanisms of Sciadonic Acid on Colonic Transit Function Through Regulating 5‐HT4/cAMP/PKA/AQP4 Signaling Pathway in STC Model Mice,” Journal of Nutritional Biochemistry 131 (2024): 109676. 38851516 10.1016/j.jnutbio.2024.109676

[148] A. P. Cherkashin , O. A. Rogachevskaja , N. V. Kabanova , P. D. Kotova , M. F. Bystrova , and S. S. Kolesnikov , “Taste Cells of the Type III Employ CASR to Maintain Steady Serotonin Exocytosis at Variable Ca(2+) in the Extracellular Medium,” Cells 11, no. 8 (2022): 1369. 35456048 10.3390/cells11081369 PMC9030112

[149] J. M. Kellum , M. R. Budhoo , A. K. Siriwardena , E. P. Smith , and S. A. Jebraili , “Serotonin Induces Cl‐ secretion in human Jejunal Mucosa in Vitro via a Nonneural Pathway at a 5‐HT4 Receptor,” American Journal of Physiology 267, no. 3 Pt 1 (1994): G357–G363. 7943231 10.1152/ajpgi.1994.267.3.G357

[150] M. R. Budhoo , R. P. Harris , and J. M. Kellum , “5‐Hydroxytryptamine‐induced Cl‐ transport Is Mediated by 5‐HT3 and 5‐HT4 Receptors in the Rat Distal Colon,” European Journal of Pharmacology 298, no. 2 (1996): 137–144. 8867100 10.1016/0014-2999(95)00752-0

[151] H. W. Kim , H. Li , H. S. Kim , et al., “Cisapride, a Selective Serotonin 5‐HT4‐receptor Agonist, Inhibits Voltage‐dependent K(+) Channels in Rabbit Coronary Arterial Smooth Muscle Cells,” Biochemical and Biophysical Research Communications 478, no. 3 (2016): 1423–1428. 27569285 10.1016/j.bbrc.2016.08.140

[152] L. Fagni , A. Dumuis , M. Sebben , and J. Bockaert , “The 5‐HT4 Receptor Subtype Inhibits K+ Current in Colliculi Neurones via Activation of a Cyclic AMP‐dependent Protein Kinase,” British Journal of Pharmacology 105, no. 4 (1992): 973–979. 1324059 10.1111/j.1476-5381.1992.tb09087.x PMC1908706

[153] P. Lecouflet , C. M. Roux , B. Potier , et al., “Interplay Between 5‐HT4 Receptors and GABAergic System Within CA1 Hippocampal Synaptic Plasticity,” Cerebral Cortex 31, no. 1 (2021): 694–701. 32935845 10.1093/cercor/bhaa253

[154] V. Sgambato , “The Serotonin 4 Receptor Subtype: A Target of Particular Interest, Especially for Brain Disorders,” International Journal of Molecular Sciences 25, no. 10 (2024): 5245. 38791281 10.3390/ijms25105245 PMC11121119

[155] S. Claeysen , P. Faye , M. Sebben , S. Taviaux , J. Bockaert , and A. Dumuis , “5‐HT4 receptors: Cloning and Expression of New Splice Variants,” Annals of the New York Academy of Sciences 861 (1998): 49–56. 9928238 10.1111/j.1749-6632.1998.tb10172.x

[156] H. R. Irving , N. Tochon‐Danguy , K. A. Chinkwo , et al., “Investigations Into the Binding Affinities of Different human 5‐HT4 Receptor Splice Variants,” Pharmacology 85, no. 4 (2010): 224–233. 20299822 10.1159/000280418

[157] T. Brattelid , A. M. Kvingedal , K. A. Krobert , et al., “Cloning, Pharmacological Characterisation and Tissue Distribution of a Novel 5‐HT4 Receptor Splice Variant, 5‐HT4(i),” Naunyn‐Schmiedebergs Archives of Pharmacology 369, no. 6 (2004): 616–628. 15118808 10.1007/s00210-004-0919-4

[158] D. L. Nelson , “5‐HT5 receptors,” Current Drug Targets CNS and Neurological Disorders 3, no. 1 (2004): 53–58. 14965244 10.2174/1568007043482606

[159] L. Aparicio‐Nava , L. A. Márquez‐García , and A. Meneses , “Effects of 5‐HT(5A) Receptor Blockade on Amnesia or Forgetting,” Behavioural Brain Research 357‐358 (2019): 98–103. 10.1016/j.bbr.2018.01.009 29330003

[160] A. M. Kinsey , A. Wainwright , R. Heavens , D. J. Sirinathsinghji , and K. R. Oliver , “Distribution of 5‐ht(5A), 5‐ht(5B), 5‐ht(6) and 5‐HT(7) Receptor mRNAs in the Rat Brain,” Brain Research Molecular Brain Research 88, no. 1‐2 (2001): 194–198. 11295248 10.1016/s0169-328x(01)00034-1

[161] D. R. Thomas , “5‐ht5A receptors as a Therapeutic Target,” Pharmacology & Therapeutics 111, no. 3 (2006): 707–714. 16516972 10.1016/j.pharmthera.2005.12.006

[162] W. D. Gwynne , M. S. Shakeel , A. Girgis‐Gabardo , et al., “Antagonists of the Serotonin Receptor 5A Target human Breast Tumor Initiating Cells,” BMC cancer 20, no. 1 (2020): 724. 32758183 10.1186/s12885-020-07193-6 PMC7404930

[163] M. Noda , S. Yasuda , M. Okada , et al., “Recombinant human Serotonin 5A Receptors Stably Expressed in C6 Glioma Cells Couple to Multiple Signal Transduction Pathways,” Journal of Neurochemistry 84, no. 2 (2003): 222–232. 12558985 10.1046/j.1471-4159.2003.01518.x

[164] M. Yamazaki , K. Harada , N. Yamamoto , et al., “ASP5736, a Novel 5‐HT5A Receptor Antagonist, Ameliorates Positive Symptoms and Cognitive Impairment in Animal Models of Schizophrenia,” European Neuropsychopharmacology 24, no. 10 (2014): 1698–1708. 25108314 10.1016/j.euroneuro.2014.07.009

[165] T. Riccioni , “5‐HT6 receptor Characterization,” International Review of Neurobiology 94 (2010): 67–88. 21081202 10.1016/B978-0-12-384976-2.00003-4

[166] A. J. Sleight , F. G. Boess , M. Bös , and A. Bourson , “The Putative 5‐ht6 Receptor: Localization and Function,” Annals of the New York Academy of Sciences 861 (1998): 91–96. 9928244 10.1111/j.1749-6632.1998.tb10178.x

[167] A. Nikiforuk , “The Procognitive Effects of 5‐HT6 Receptor Ligands in Animal Models of Schizophrenia,” Reviews in the Neurosciences 25, no. 3 (2014): 367–382. 24501158 10.1515/revneuro-2014-0005

[168] Y. X. Zhang , M. Yang , F. Liang , et al., “The Pronociceptive Role of 5‐HT(6) Receptors in Ventrolateral Orbital Cortex in a Rat Formalin Test Model,” Neurochemistry International 131 (2019): 104562. 31580911 10.1016/j.neuint.2019.104562

[169] N. Gottlieb , T. Y. Li , A. H. Young , and P. R. Stokes , “The 5‐HT7 Receptor System as a Treatment Target for Mood and Anxiety Disorders: A Systematic Review,” Journal of Psychopharmacology 37, no. 12 (2023): 1167–1181. 37994803 10.1177/02698811231211228 PMC10714716

[170] E. Gellynck , K. Heyninck , K. W. Andressen , et al., “The Serotonin 5‐HT7 Receptors: Two Decades of Research,” Experimental Brain Research 230, no. 4 (2013): 555–568. 24042216 10.1007/s00221-013-3694-y

[171] J. M. Monti and H. Jantos , “The Role of Serotonin 5‐HT7 Receptor in Regulating Sleep and Wakefulness,” Reviews in the Neurosciences 25, no. 3 (2014): 429–437. 24681431 10.1515/revneuro-2014-0016

[172] K. Fukuyama , E. Motomura , and M. Okada , “Brexpiprazole Reduces 5‐HT7 Receptor Function on Astroglial Transmission Systems,” International Journal of Molecular Sciences 23, no. 12 (2022): 6571. 35743014 10.3390/ijms23126571 PMC9223571

[173] P. B. Hedlund and J. G. Sutcliffe , “Functional, Molecular and Pharmacological Advances in 5‐HT7 Receptor Research,” Trends in Pharmacological Sciences 25, no. 9 (2004): 481–486. 15559250 10.1016/j.tips.2004.07.002

[174] S. Lenglet , E. Louiset , C. Delarue , H. Vaudry , and V. Contesse , “Activation of 5‐HT(7) Receptor in Rat Glomerulosa Cells Is Associated With an Increase in Adenylyl Cyclase Activity and Calcium Influx Through T‐type Calcium Channels,” Endocrinology 143, no. 5 (2002): 1748–1760. 11956157 10.1210/endo.143.5.8817

[175] A. Matthys , G. Haegeman , K. Van Craenenbroeck , and P. Vanhoenacker , “Role of the 5‐HT7 Receptor in the central Nervous System: From Current Status to Future Perspectives,” Molecular Neurobiology 43, no. 3 (2011): 228–253. 21424680 10.1007/s12035-011-8175-3

[176] M. Kolaj , L. Zhang , and L. P. Renaud , “Novel Coupling Between TRPC‐Like and KNa Channels Modulates Low Threshold Spike‐induced Afterpotentials in Rat Thalamic Midline Neurons,” Neuropharmacology 86 (2014): 88–96. 25014020 10.1016/j.neuropharm.2014.06.023

[177] F. Kawahara , H. Saito , and H. Katsuki , “Inhibition by 5‐HT7 Receptor Stimulation of GABAA Receptor‐activated Current in Cultured Rat Suprachiasmatic Neurones,” The Journal of Physiology 478, no. Pt 1 (1994): 67–73. 7965836 10.1113/jphysiol.1994.sp020230 PMC1155645

[178] E. M. Chapin and R. Andrade , “A 5‐HT(7) Receptor‐mediated Depolarization in the Anterodorsal Thalamus. II. Involvement of the Hyperpolarization‐activated Current I(h),” Journal of Pharmacology and Experimental Therapeutics 297, no. 1 (2001): 403–409. 11259569

[179] S. Saha and J. González‐Maeso , “The Crosstalk Between 5‐HT(2A)R and mGluR2 in Schizophrenia,” Neuropharmacology 230 (2023): 109489. 36889432 10.1016/j.neuropharm.2023.109489 PMC10103009

[180] R. Pan , L. Wang , X. Xu , et al., “Crosstalk Between the Gut Microbiome and Colonic Motility in Chronic Constipation: Potential Mechanisms and Microbiota Modulation,” Nutrients 14, no. 18 (2022): 3704. 36145079 10.3390/nu14183704 PMC9505360

[181] T. M. Eriksson , S. Holst , T. L. Stan , et al., “5‐HT1A and 5‐HT7 Receptor Crosstalk in the Regulation of Emotional Memory: Implications for Effects of Selective Serotonin Reuptake Inhibitors,” Neuropharmacology 63, no. 6 (2012): 1150–1160. 22801295 10.1016/j.neuropharm.2012.06.061

[182] H. Zhang , Y. Hasegawa , M. Suzuki , et al., “Mouse Enteric Neurons Control Intestinal Plasmacytoid Dendritic Cell Function via Serotonin‐HTR7 Signaling,” Nature Communications 15, no. 1 (2024): 9237. 10.1038/s41467-024-53545-2 PMC11511829 39455564

[183] C. B. M. Poulie , N. Liu , A. A. Jensen , and L. Bunch , “Design, Synthesis, and Pharmacological Characterization of Heterobivalent Ligands for the Putative 5‐HT(2A)/mGlu(2) Receptor Complex,” Journal of Medicinal Chemistry 63, no. 17 (2020): 9928–9949. 32815361 10.1021/acs.jmedchem.0c01058

[184] L. Albizu , T. Holloway , J. González‐Maeso , and S. C. Sealfon , “Functional Crosstalk and Heteromerization of Serotonin 5‐HT2A and Dopamine D2 Receptors,” Neuropharmacology 61, no. 4 (2011): 770–777. 21645528 10.1016/j.neuropharm.2011.05.023 PMC3556730

[185] D. Ibi , “Role of Interaction of mGlu2 and 5‐HT(2A) Receptors in Antipsychotic Effects,” Pharmacology Biochemistry and Behavior 221 (2022): 173474. 36244526 10.1016/j.pbb.2022.173474

[186] A. Taddeucci , G. Olivero , A. Roggeri , et al., “Presynaptic 5‐HT(2A)‐mGlu2/3 Receptor‐Receptor Crosstalk in the Prefrontal Cortex: Metamodulation of Glutamate Exocytosis,” Cells 11, no. 19 (2022): 3035. 36230998 10.3390/cells11193035 PMC9562019

[187] L. Wischhof and M. Koch , “5‐HT2A and mGlu2/3 Receptor Interactions: On Their Relevance to Cognitive Function and Psychosis,” Behavioural Pharmacology 27, no. 1 (2016): 1–11. 26292187 10.1097/FBP.0000000000000183

[188] Y. Chang‐Halabi , J. Cordero , X. Sarabia , D. Villalobos , and N. P. Barrera , “Crosstalking Interactions Between P2X4 and 5‐HT(3A) Receptors,” Neuropharmacology 236 (2023): 109574. 37156336 10.1016/j.neuropharm.2023.109574

[189] G. Burnat , P. Brański , J. Solich , et al., “The Functional Cooperation of 5‐HT(1A) and mGlu4R in HEK‐293 Cell Line,” Pharmacology Reports 72, no. 5 (2020): 1358–1369. 10.1007/s43440-020-00114-1 PMC7550284 32472388

[190] B. Chruścicka , C. S. M. Cowan , and S. E. Wallace Fitzsimons , “Molecular, Biochemical and Behavioural Evidence for a Novel Oxytocin Receptor and Serotonin 2C Receptor Heterocomplex,” Neuropharmacology 183 (2021): 108394. 33188842 10.1016/j.neuropharm.2020.108394

[191] Y. Wang , D. Wang , Y. Chen , X. Fang , L. Yu , and C. Zhang , “A Novel Synthetic Interfering Peptide Tat‐3L4F Attenuates Olanzapine‐Induced Weight Gain through Disrupting Crosstalk between Serotonin Receptor 2C and Protein Phosphatase and Tensin Homolog in Rats,” The International Journal of Neuropsychopharmacology 23, no. 8 (2020): 481–490. 32710540 10.1093/ijnp/pyaa001 PMC7689208

[192] D. Hoyer , J. P. Hannon , and G. R. Martin , “Molecular, Pharmacological and Functional Diversity of 5‐HT Receptors,” Pharmacology Biochemistry and Behavior 71, no. 4 (2002): 533–554. 11888546 10.1016/s0091-3057(01)00746-8

[193] R. R. Perim , D. P. Fields , and G. S. Mitchell , “Cross‐talk Inhibition Between 5‐HT(2B) and 5‐HT(7) Receptors in Phrenic Motor Facilitation via NADPH Oxidase and PKA,” American Journal of Physiology. Regulatory, Integrative and Comparative Physiology 314, no. 5 (2018): R709–R715. 29384698 10.1152/ajpregu.00393.2017 PMC6008113

[194] H. Salkin , G. Satir‐Basaran , S. Korkmaz , Z. Burcin Gonen , and K. Erdem Basaran , “Mesenchymal Stem Cell‐derived Conditioned Medium and Methysergide Give Rise to Crosstalk Inhibition of 5‐HT2A and 5‐HT7 Receptors in Neuroblastoma Cells,” Brain Research 1808 (2023): 148354. 36997105 10.1016/j.brainres.2023.148354

[195] K. B. Fink and M. Göthert , “5‐HT Receptor Regulation of Neurotransmitter Release,” Pharmacological Reviews 59, no. 4 (2007): 360–417. 18160701 10.1124/pr.107.07103

[196] M. Takahashi , Y. Kobayashi , K. Ando , Y. Saito , and S. I. Hisanaga , “Cyclin‐dependent Kinase 5 Promotes Proteasomal Degradation of the 5‐HT(1A) Receptor via Phosphorylation,” Biochemical and Biophysical Research Communications 510, no. 3 (2019): 370–375. 30712943 10.1016/j.bbrc.2019.01.093

[197] Y. Liu , A. W. Gibson , M. R. Levinstein , A. J. Lesiak , S. E. Ong , and J. F. Neumaier , “5‐HT(1B) Receptor‐Mediated Activation of ERK1/2 Requires both Gα(i/o) and β‐Arrestin Proteins,” Acs Chemical Neuroscience 10, no. 7 (2019): 3143–3153. 30946562 10.1021/acschemneuro.8b00596 PMC7217031

[198] A. C. Magalhaes , K. D. Holmes , L. B. Dale , et al., “CRF Receptor 1 Regulates Anxiety Behavior via Sensitization of 5‐HT2 Receptor Signaling,” Nature Neuroscience 13, no. 5 (2010): 622–629. 20383137 10.1038/nn.2529 PMC2862362

[199] C. H. Chen , M. M. Paing , and J. Trejo , “Termination of Protease‐activated Receptor‐1 Signaling by Beta‐arrestins Is Independent of Receptor Phosphorylation,” Journal of Biological Chemistry 279, no. 11 (2004): 10020–10031. 14699102 10.1074/jbc.M310590200

[200] J. Mialet , R. Fischmeister , and F. Lezoualc'h , “Characterization of human 5‐HT4(d) Receptor Desensitization in CHO Cells,” British Journal of Pharmacology 138, no. 3 (2003): 445–452. 12569069 10.1038/sj.bjp.0705061 PMC1573685

[201] J. Janetzko , R. Kise , B. Barsi‐Rhyne , et al., “Membrane Phosphoinositides Regulate GPCR‐β‐arrestin Complex Assembly and Dynamics,” Cell 185, no. 24 (2022): 4560–4573. e19. 36368322 10.1016/j.cell.2022.10.018 PMC10030194

[202] S. Ragini , A. Saini , and I. Mani , “Endocytosis and Signaling of 5‐HT1A Receptor,” Progress in Molecular Biology and Translational Science 196 (2023): 113–123. 36813354 10.1016/bs.pmbts.2022.11.002

[203] S. Wang , H. Liu , J. B. Roberts , et al., “Prolonged Ethanol Exposure Modulates Constitutive Internalization and Recycling of 5‐HT1A Receptors,” Journal of Neurochemistry 160, no. 4 (2022): 469–481. 34928513 10.1111/jnc.15564 PMC8828711

[204] I. Gaidarov , J. Frazer , X. Chen , et al., “Mechanisms of Constitutive and Agonist‐induced 5‐HT(2B) Internalization, Persistent Endosomal Signaling and Paradoxical Regulation of Agonist Pharmacology,” Cell Signalling 131 (2025): 111769. 40169062 10.1016/j.cellsig.2025.111769

[205] H. A. Dunn , C. Walther , G. Y. Yuan , F. A. Caetano , C. M. Godin , and S. S. Ferguson , “Role of SAP97 in the Regulation of 5‐HT2AR Endocytosis and Signaling,” Molecular Pharmacology 86, no. 3 (2014): 275–283. 24989932 10.1124/mol.114.093476

[206] D. Kowal , J. Zhang , S. Nawoschik , et al., “The C‐terminus of Gi family G‐proteins as a Determinant of 5‐HT(1A) Receptor Coupling,” Biochemical and Biophysical Research Communications 294, no. 3 (2002): 655–659. 12056819 10.1016/S0006-291X(02)00535-1

[207] M. Soiza‐Reilly and K. G. Commons , “Glutamatergic Drive of the Dorsal Raphe Nucleus,” Journal of Chemical Neuroanatomy 41, no. 4 (2011): 247–255. 21550397 10.1016/j.jchemneu.2011.04.004 PMC3150565

[208] B. Y. Li and W. H. Li , “Effects of 5‐HT Released From Platelets on Thrombin‐induced Aggregation and ATP Release in Rabbit Platelets in Vitro,” Zhongguo Yao Li Xue Bao = Acta Pharmacologica Sinica 19, no. 4 (1998): 383–386. 10375791

[209] W. D. Gwynne , R. M. Hallett , A. Girgis‐Gabardo , et al., “Serotonergic System Antagonists Target Breast Tumor Initiating Cells and Synergize With Chemotherapy to Shrink human Breast Tumor Xenografts,” Oncotarget 8, no. 19 (2017): 32101–32116. 28404880 10.18632/oncotarget.16646 PMC5458271

[210] M. Masab and M. W. Saif , “Telotristat ethyl: Proof of Principle and the First Oral Agent in the Management of Well‐differentiated Metastatic Neuroendocrine Tumor and Carcinoid Syndrome Diarrhea,” Cancer Chemotheraphy and Pharmacology 80, no. 6 (2017): 1055–1062. 10.1007/s00280-017-3462-y 29051994

[211] M. A. Morse , E. Liu , V. N. Joish , et al., “Antiproliferative Effects of Telotristat Ethyl in Patients With Neuroendocrine Tumors: The TELEACE Real‐World Chart Review Study,” Cancer Management and Research 12 (2020): 6607–6614. 32801896 10.2147/CMAR.S261257 PMC7402667

[212] M. A. Schneider , L. Heeb , M. M. Beffinger , et al., “Attenuation of Peripheral Serotonin Inhibits Tumor Growth and Enhances Immune Checkpoint Blockade Therapy in Murine Tumor Models,” Science Translational Medicine 13, no. 611 (2021): eabc8188. 34524861 10.1126/scitranslmed.abc8188

[213] D. H. Tow , C. G. Tran , L. C. Borbon , et al., “Inhibition of Serotonin Biosynthesis in Neuroendocrine Neoplasm Suppresses Tumor Growth in Vivo,” BioRxiv (2023).

[214] J. Zhang , Z. Guo , Q. Xie , C. Zhong , X. Gao , and Q. Yang , “Tryptophan Hydroxylase 1 Drives Glioma Progression by Modulating the Serotonin/L1CAM/NF‐kappaB Signaling Pathway,” BMC cancer 22, no. 1 (2022): 457. 35473609 10.1186/s12885-022-09569-2 PMC9044587

[215] G. Alpini , P. Invernizzi , E. Gaudio , et al., “Serotonin Metabolism Is Dysregulated in Cholangiocarcinoma, Which Has Implications for Tumor Growth,” Cancer Research 68, no. 22 (2008): 9184–9193. 19010890 10.1158/0008-5472.CAN-08-2133 PMC2593938

[216] L. R. Frick , M. Rapanelli , M. L. Arcos , G. A. Cremaschi , and A M. Genaro , “Oral Administration of Fluoxetine Alters the Proliferation/Apoptosis Balance of Lymphoma Cells and Up‐regulates T Cell Immunity in Tumor‐bearing Mice,” European Journal of Pharmacology 659, no. 2‐3 (2011): 265–272. 21497159 10.1016/j.ejphar.2011.03.037

[217] E. Saponara , M. Visentin , F. Baschieri , et al., “Serotonin Uptake Is Required for Rac1 Activation in Kras‐induced Acinar‐to‐ductal Metaplasia in the Pancreas,” Journal of Pathology 246, no. 3 (2018): 352–365. 30058725 10.1002/path.5147

[218] D. Peer , Y. Dekel , D. Melikhov , and R. Margalit , “Fluoxetine Inhibits Multidrug Resistance Extrusion Pumps and Enhances Responses to Chemotherapy in Syngeneic and in human Xenograft Mouse Tumor Models,” Cancer Research 64, no. 20 (2004): 7562–7569. 15492283 10.1158/0008-5472.CAN-03-4046

[219] V. van Noort , S. Scholch , M. Iskar , et al., “Novel Drug Candidates for the Treatment of Metastatic Colorectal Cancer Through Global Inverse Gene‐expression Profiling,” Cancer Research 74, no. 20 (2014): 5690–5699. 25038229 10.1158/0008-5472.CAN-13-3540

[220] X. Jiang , W. Lu , X. Shen , et al., “Repurposing Sertraline Sensitizes Non‐small Cell Lung Cancer Cells to Erlotinib by Inducing Autophagy,” JCI Insight 3, no. 11 (2018): e98921. 29875309 10.1172/jci.insight.98921 PMC6124398

[221] Y. Shen , X. Luo , H. Li , Z. Chen , Q. Guan , and L. Cheng , “Simple and Reliable Serotonin Assay in human Serum by LC‐MS/MS Method Coupled With One Step Protein Precipitation for Clinical Testing in Patients With Carcinoid Tumors,” Journal of Chromatography. B, Analytical Technologies in the Biomedical and Life Sciences 1158 (2020): 122395. 33091677 10.1016/j.jchromb.2020.122395

[222] M. Mitsis , G. Markopoulos , G. A. Alexiou , et al., “Antiproliferative and Cytotoxic Action of N‐(p‐coumaroyl) serotonin in Lung Cancer Cells,” Journal of Buon 23, no. 6 (2018): 1693–1698. 30610796

[223] J. B. Wu , T. P. Lin , J. D. Gallagher , et al., “Monoamine Oxidase A Inhibitor‐near‐infrared Dye Conjugate Reduces Prostate Tumor Growth,” Journal of the American Chemical Society 137, no. 6 (2015): 2366–2374. 25585152 10.1021/ja512613j

[224] S. Kushal , W. Wang , V. P. Vaikari , et al., “Monoamine Oxidase A (MAO A) Inhibitors Decrease Glioma Progression,” Oncotarget 7, no. 12 (2016): 13842–13853. 26871599 10.18632/oncotarget.7283 PMC4924682

[225] P. C. Li , S. Y. Chen , D. Xiangfei , C. Mao , C. H. Wu , and J C. Shih , “PAMs Inhibits Monoamine Oxidase a Activity and Reduces Glioma Tumor Growth, a Potential Adjuvant Treatment for Glioma,” BMC Complementary Medicine and Therapies 20, no. 1 (2020): 252. 32799864 10.1186/s12906-020-03041-z PMC7429690

[226] Y. C. Wang , X. Wang , J. Yu , et al., “Targeting Monoamine Oxidase A‐regulated Tumor‐associated Macrophage Polarization for Cancer Immunotherapy,” Nature Communications 12, no. 1 (2021): 3530. 10.1038/s41467-021-23164-2 PMC8192781 34112755

[227] X. Wang , B. Li , Y. J. Kim , et al., “Targeting Monoamine Oxidase A for T Cell‐based Cancer Immunotherapy,” Science Immunology 6, no. 59 (2021): eabh2383–eabh2383. 33990379 10.1126/sciimmunol.abh2383

[228] P. C. Li , I. N. Siddiqi , A. Mottok , et al., “Monoamine Oxidase A Is Highly Expressed in Classical Hodgkin Lymphoma,” Journal of Pathology 243, no. 2 (2017): 220–229. 28722111 10.1002/path.4944 PMC5605421

[229] K. Wang , J. Luo , S. Yeh , et al., “The MAO Inhibitors Phenelzine and Clorgyline Revert Enzalutamide Resistance in Castration Resistant Prostate Cancer,” Nature Communications 11, no. 1 (2020): 2689. 10.1038/s41467-020-15396-5 PMC7264333 32483206

[230] M. Vanneste , A. Venzke , S. Guin , et al., “The Anti‐cancer Efficacy of a Novel Phenothiazine Derivative Is Independent of Dopamine and Serotonin Receptor Inhibition,” Frontiers in oncology 13 (2023): 1295185. 37909019 10.3389/fonc.2023.1295185 PMC10613967

[231] N. Dizeyi , P. Hedlund , A. Bjartell , M. Tinzl , K. Austild‐Tasken , and P A. Abrahamsson , “Serotonin Activates MAP Kinase and PI3K/Akt Signaling Pathways in Prostate Cancer Cell Lines,” Urologic Oncology 29, no. 4 (2011): 436–445. 19926313 10.1016/j.urolonc.2009.09.013

[232] J. Jose , C. D. J. Tavares , N. D. Ebelt , et al., “Serotonin Analogues as Inhibitors of Breast Cancer Cell Growth,” Acs Medicinal Chemistry Letters 8, no. 10 (2017): 1072–1076. 29057053 10.1021/acsmedchemlett.7b00282 PMC5641961

[233] S. S. Kolan , T. Lidstrom , T. Mediavilla , et al., “Growth‐inhibition of Cell Lines Derived From B Cell Lymphomas Through Antagonism of Serotonin Receptor Signaling,” Scientific Reports 9, no. 1 (2019): 4276. 30862884 10.1038/s41598-019-40825-x PMC6414675

[234] C. Soll , M. O. Riener , C. E. Oberkofler , et al., “Expression of Serotonin Receptors in human Hepatocellular Cancer,” Clinical Cancer Research 18, no. 21 (2012): 5902–5910. 23087410 10.1158/1078-0432.CCR-11-1813

[235] H. Sui , H. Xu , Q. Ji , et al., “5‐hydroxytryptamine Receptor (5‐HT1DR) Promotes Colorectal Cancer Metastasis by Regulating Axin1/Beta‐catenin/MMP‐7 Signaling Pathway,” Oncotarget 6, no. 28 (2015): 25975–25987. 26214021 10.18632/oncotarget.4543 PMC4694879

[236] X. Qin , J. Li , S. Wang , et al., “Serotonin/HTR1E Signaling Blocks Chronic Stress‐promoted Progression of Ovarian Cancer,” Theranostics 11, no. 14 (2021): 6950–6965. 34093864 10.7150/thno.58956 PMC8171092

[237] C. S. Sreevidya , N. M. Khaskhely , A. Fukunaga , P. Khaskina , and S E. Ullrich , “Inhibition of Photocarcinogenesis by Platelet‐activating Factor or Serotonin Receptor Antagonists,” Cancer Research 68, no. 10 (2008): 3978–3984. 18483284 10.1158/0008-5472.CAN-07-6132 PMC2394717

[238] C. Liang , W. Chen , and X. Zhi , “Serotonin Promotes the Proliferation of Serum‐deprived Hepatocellular Carcinoma Cells via Upregulation of FOXO3a,” Molecular cancer 12, no. 1 (2013): 14. 23418729 10.1186/1476-4598-12-14 PMC3601970

[239] M. Asada , S. Ebihara , S. Yamanda , et al., “Depletion of Serotonin and Selective Inhibition of 2B Receptor Suppressed Tumor Angiogenesis by Inhibiting Endothelial Nitric Oxide Synthase and Extracellular Signal‐regulated Kinase 1/2 Phosphorylation,” Neoplasia 11, no. 4 (2009): 408–417. 19308295 10.1593/neo.81630 PMC2657884

[240] S. Liu , R. Miao , M. Zhai , et al., “Effects and Related Mechanisms of Serotonin on Malignant Biological Behavior of Hepatocellular Carcinoma via Regulation of Yap,” Oncotarget 8, no. 29 (2017): 47412–47424. 28537892 10.18632/oncotarget.17658 PMC5564575

[241] S. H. Jiang , J. Li , F. Y. Dong , et al., “Increased Serotonin Signaling Contributes to the Warburg Effect in Pancreatic Tumor Cells under Metabolic Stress and Promotes Growth of Pancreatic Tumors in Mice,” Gastroenterology 153, no. 1 (2017): 277–291. e19. 28315323 10.1053/j.gastro.2017.03.008

[242] M. de las Casas‐Engel , A. Dominguez‐Soto , E. Sierra‐Filardi , et al., “Serotonin Skews human Macrophage Polarization Through HTR2B and HTR7,” Journal of Immunology 190, no. 5 (2013): 2301–2310. 10.4049/jimmunol.1201133 23355731

[243] C. Weidmann , J. Berube , L. Piquet , A. de la Fouchardiere , and S. Landreville , “Expression of the Serotonin Receptor 2B in Uveal Melanoma and Effects of an Antagonist on Cell Lines,” Clinical & Experimental Metastasis 35, no. 3 (2018): 123–134. 29696577 10.1007/s10585-018-9894-x

[244] B. Svejda , M. Kidd , F. Giovinazzo , et al., “The 5‐HT(2B) Receptor Plays a Key Regulatory Role in both Neuroendocrine Tumor Cell Proliferation and the Modulation of the Fibroblast Component of the Neoplastic Microenvironment,” Cancer 116, no. 12 (2010): 2902–2912. 20564397 10.1002/cncr.25049

[245] N. Dizeyi , A. Bjartell , P. Hedlund , K. A. Tasken , V. Gadaleanu , and P A. Abrahamsson , “Expression of Serotonin Receptors 2B and 4 in human Prostate Cancer Tissue and Effects of Their Antagonists on Prostate Cancer Cell Lines,” European Urology 47, no. 6 (2005): 895–900. 15925089 10.1016/j.eururo.2005.02.006

[246] F. Jaffré , J. Callebert , A. Sarre , et al., “Involvement of the Serotonin 5‐HT2B Receptor in Cardiac Hypertrophy Linked to Sympathetic Stimulation: Control of Interleukin‐6, Interleukin‐1beta, and Tumor Necrosis Factor‐alpha Cytokine Production by Ventricular Fibroblasts,” Circulation 110, no. 8 (2004): 969–974. 15302781 10.1161/01.CIR.0000139856.20505.57

[247] J. S. Lee , S. Y. Park , N. Y. Kim , et al., “Anti‐Tumor Potential of a 5‐HT3 Receptor Antagonist as a Novel Autophagy Inducer in Lung Cancer: A Retrospective Clinical Study With in Vitro Confirmation,” Journal of Clinical Medicine 8, no. 9 (2019): 1380. 31484445 10.3390/jcm8091380 PMC6780215

[248] Y. Merrouche , G. Catimel , P. Rebattu , et al., “A Phase I Antiemetic Study of MDL 73,147EF, a Novel 5‐hydroxytryptamine Antagonist in Cancer Patients Receiving Emetogenic Chemotherapy,” Annals of Oncology 5, no. 6 (1994): 549–551. 7918128 10.1093/oxfordjournals.annonc.a058911

[249] O. Mir , J. P. Durand , P. Boudou‐Rouquette , et al., “Interaction Between Serotonin Reuptake Inhibitors, 5‐HT3 Antagonists, and NK1 Antagonists in Cancer Patients Receiving Highly Emetogenic Chemotherapy: A Case‐control Study,” Supportive Care in Cancer 20, no. 9 (2012): 2235–2239. 22644261 10.1007/s00520-012-1503-y

[250] J. J. Curtis , N. T. K. Vo , C. B. Seymour , and C E. Mothersill , “5‐HT(2A) and 5‐HT(3) Receptors Contribute to the Exacerbation of Targeted and Non‐targeted Effects of Ionizing Radiation‐induced Cell Death in human Colon Carcinoma Cells,” International Journal of Radiation Biology 96, no. 4 (2020): 482–490. 31846381 10.1080/09553002.2020.1704911

[251] J. Ishizuka , A. C. Hsieh , C. M. Townsend Jr. , and J. C. Thompson , “Effect of 5‐HT3 Receptor Antagonist (ondansetron) on Functioning human Pancreatic Carcinoid Cells,” Surgical Oncology 2, no. 4 (1993): 221–225. 8252212 10.1016/0960-7404(93)90010-v

[252] H. Qiao , Y. B. Wang , Y. M. Gao , and L L. Bi , “Prucalopride Inhibits the Glioma Cells Proliferation and Induces Autophagy via AKT‐mTOR Pathway,” BMC Neurology [Electronic Resource] 18, no. 1 (2018): 80. 29866060 10.1186/s12883-018-1083-7 PMC5985575

[253] T. Nishikawa , N. H. Tsuno , Y. Shuno , et al., “Antiangiogenic Effect of a Selective 5‐HT4 Receptor Agonist,” Journal of Surgical Research 159, no. 2 (2010): 696–704. 19515383 10.1016/j.jss.2008.11.004

[254] Q. Lu , Y. Ding , Y. Li , and Q. Lu , “5‐HT Receptor Agonist Valerenic Acid Enhances the Innate Immunity Signal and Suppresses Glioblastoma Cell Growth and Invasion,” International Journal of Biological Sciences 16, no. 12 (2020): 2104–2115. 32549758 10.7150/ijbs.44906 PMC7294948

[255] S. Fatima , X. Shi , Z. Lin , et al., “5‐Hydroxytryptamine Promotes Hepatocellular Carcinoma Proliferation by Influencing Beta‐catenin,” Molecular Oncology 10, no. 2 (2016): 195–212. 26474915 10.1016/j.molonc.2015.09.008 PMC5528951

[256] B. Svejda , M. Kidd , A. Timberlake , et al., “Serotonin and the 5‐HT7 Receptor: The Link Between Hepatocytes, IGF‐1 and Small Intestinal Neuroendocrine Tumors,” Cancer Science 104, no. 7 (2013): 844–855. 23578138 10.1111/cas.12174 PMC7657194

[257] I. Cinar , B. Sirin , Z. Halici , S. S. Palabiyik‐Yucelik , E. Akpinar , and E. Cadirci , “5‐HT7 receptors as a New Target for Prostate Cancer Physiopathology and Treatment: An Experimental Study on PC‐3 Cells and FFPE Tissues,” Naunyn‐Schmiedebergs Archives of Pharmacology 394, no. 6 (2021): 1205–1213. 33528589 10.1007/s00210-021-02051-z

[258] J. Gautam , S. Banskota , S. C. Regmi , et al., “Tryptophan Hydroxylase 1 and 5‐HT(7) Receptor Preferentially Expressed in Triple‐negative Breast Cancer Promote Cancer Progression Through Autocrine Serotonin Signaling,” Molecular cancer 15, no. 1 (2016): 75. 27871326 10.1186/s12943-016-0559-6 PMC5117586

[259] K. Lieb , L. Biersack , A. Waschbisch , et al., “Serotonin via 5‐HT7 Receptors Activates p38 Mitogen‐activated Protein Kinase and Protein Kinase C Epsilon Resulting in Interleukin‐6 Synthesis in human U373 MG Astrocytoma Cells,” Journal of Neurochemistry 93, no. 3 (2005): 549–559. 15836614 10.1111/j.1471-4159.2005.03079.x

[260] X. Du , T. Wang , Z. Wang , et al., “5‐HT(7) Receptor Contributes to Proliferation, Migration and Invasion in NSCLC Cells,” OncoTargets and Therapy 13 (2020): 2139–2151. 32210580 10.2147/OTT.S244339 PMC7071786

[261] M. Camilleri , “LX‐1031, a Tryptophan 5‐hydroxylase Inhibitor That Reduces 5‐HT Levels for the Potential Treatment of Irritable Bowel Syndrome,” Idrugs 13, no. 12 (2010): 921–928. 21154152

[262] C. M. Ervin and A. W. Mangel , “Clinical Trials in Irritable Bowel Syndrome: A Review,” Reviews on Recent Clinical Trials 8, no. 1 (2013): 9–22. 23130604 10.2174/1574887111308010003

[263] P. M. Brown , D. A. Drossman , A. J. Wood , et al., “The Tryptophan Hydroxylase Inhibitor LX1031 Shows Clinical Benefit in Patients With Nonconstipating Irritable Bowel Syndrome,” Gastroenterology 141, no. 2 (2011): 507–516. 21684281 10.1053/j.gastro.2011.05.005 PMC4905727

[264] M. J. da Rocha , C. S. Pires , M. H. Presa , et al., “Involvement of the Serotonergic System in the Antidepressant‐Like Effect of 1‐(phenylselanyl)‐2‐(p‐tolyl)indolizine in Mice,” Psychopharmacology 240, no. 2 (2023): 373–389. 36645465 10.1007/s00213-023-06313-x

[265] J. Fu , Q. Y. Yang , K. Sai , et al., “TGM2 inhibition Attenuates ID1 Expression in CD44‐high Glioma‐initiating Cells,” Neuro‐oncology 15, no. 10 (2013): 1353–1365. 23877317 10.1093/neuonc/not079 PMC3779037

[266] D. P. Figgitt and M. K. Fluvoxamine , “An Updated Review of Its Use in the Management of Adults With Anxiety Disorders,” Drugs 60, no. 4 (2000): 925–954. 11085201 10.2165/00003495-200060040-00006

[267] Vilazodone . Drugs and Lactation Database (LactMed®). National Institute of Child Health and Human Development; 2006. 30000853

[268] R. Aghajani , M. Tavalaee , N. Sadeghi , et al., “Paroxetine Treatment in an Animal Model of Depression Improves Sperm Quality,” PLoS ONE 17, no. 12 (2022): e0271217. 36480503 10.1371/journal.pone.0271217 PMC9731436

[269] M. Bourin , P. Chue , and Y. Guillon , “Paroxetine: A Review,” CNS Drug Reviews 7, no. 1 (2001): 25–47. 11420571 10.1111/j.1527-3458.2001.tb00189.x PMC6741642

[270] R. Kumar , S. N. Ali , S. Saha , and S. Bhattacharjee , “SSRI Induced Hypnic Jerks: A Case Series,” Indian Journal of Psychiatry 65, no. 7 (2023): 785–788. 37645359 10.4103/indianjpsychiatry.indianjpsychiatry_207_23 PMC10461585

[271] T. Sharp and H. Collins , “Mechanisms of SSRI Therapy and Discontinuation,” Current Topics in Behavioral Neurosciences 66 (2024): 21–47. 37955823 10.1007/7854_2023_452

[272] G. Crépeau‐Gendron , H. K. Brown , C. Shorey , et al., “Association Between Citalopram, Escitalopram and QTc Prolongation in a Real‐world Geriatric Setting,” Journal of Affective Disorders 250 (2019): 341–345. 30877856 10.1016/j.jad.2019.02.060

[273] P. F. Smith and C. L. Darlington , “A Possible Explanation for Dizziness Following SSRI Discontinuation,” Acta Oto‐Laryngologica 130, no. 9 (2010): 981–983. 20144124 10.3109/00016481003602082

[274] J. K. Mortensen and G. Andersen , “Safety Considerations for Prescribing SSRI Antidepressants to Patients at Increased Cardiovascular Risk,” Expert Opinion on Drug Safety 21, no. 4 (2022): 467–475. 34569395 10.1080/14740338.2022.1986001

[275] A. Skalkidou , I. Sundström‐Poromaa , A. Wikman , S. Hesselman , A. K. Wikström , and E. Elenis , “SSRI Use During Pregnancy and Risk for Postpartum Haemorrhage: A National Register‐based Cohort Study in Sweden,” Bjog 127, no. 11 (2020): 1366–1373. 32162458 10.1111/1471-0528.16210

[276] H. Brauch , T. E. Mürdter , M. Eichelbaum , and M. Schwab , “Pharmacogenomics of Tamoxifen Therapy,” Clinical Chemistry 55, no. 10 (2009): 1770–1782. 19574470 10.1373/clinchem.2008.121756

[277] R. H. Howland , “MAOI Antidepressant Drugs,” Journal of Psychosocial Nursing and Mental Health Services 44, no. 6 (2006): 9–12. 10.3928/02793695-20060601-02 16789588

[278] V. Van den Eynde , G. Parker , H. G. Ruhé , et al., “On the Origins of MAOI Misconceptions: Reaffirming Their Role in Melancholic Depression,” Psychopharmacology Bulletin 53, no. 3 (2023): 35–54. 37601082 10.64719/pb.4466 PMC10434306

[279] T. Sub Laban and A. Saadabadi , “Monoamine Oxidase Inhibitors (MAOI),” StatPearls (StatPearls Publishing. Copyright © 2025, StatPearls Publishing LLC., 2025). 30969670

[280] V. Van den Eynde , W. R. Abdelmoemin , M. M. Abraham , et al., “The Prescriber's Guide to Classic MAO Inhibitors (phenelzine, tranylcypromine, isocarboxazid) for Treatment‐resistant Depression,” CNS Spectrums 28, no. 4 (2023): 427–440. 35837681 10.1017/S1092852922000906

[281] P. Riederer , L. Lachenmayer , and G. Laux , “Clinical Applications of MAO‐inhibitors,” Current Medicinal Chemistry 11, no. 15 (2004): 2033–2043. 15279566 10.2174/0929867043364775

[282] B. Bandelow , J. Zohar , E. Hollander , S. Kasper , and H. J. Möller , “World Federation of Societies of Biological Psychiatry (WFSBP) Guidelines for the Pharmacological Treatment of Anxiety, Obsessive‐compulsive and Posttraumatic Stress Disorders,” The World Journal of Biological Psychiatry: The Official Journal of the World Federation of Societies of Biological Psychiatry 3, no. 4 (2002): 171–199. 12516310 10.3109/15622970209150621

[283] Ł. Grabowski , “[Monoamine oxidase inhibitors (MAOI): Pharmacology, metabolism and application in the treatment of depression],” Postepy Biochemii 67, no. 2 (2021): 130–140. Inhibitory monoaminooksydazy (IMAO): farmakologia, metabolizm i zastosowanie w terapii depresji o różnej etiologii. 34378889 10.18388/pb.2021_382

[284] W. J. Scotton , L. J. Hill , A. C. Williams , and N. M. Barnes , “Serotonin Syndrome: Pathophysiology, Clinical Features, Management, and Potential Future Directions,” International Journal of Tryptophan Research 12 (2019): 1178646919873925. 31523132 10.1177/1178646919873925 PMC6734608

[285] J. P. Feighner , W. F. Boyer , D. L. Tyler , and R. J. Neborsky , “Adverse Consequences of Fluoxetine‐MAOI Combination Therapy,” Journal of Clinical Psychiatry 51, no. 6 (1990): 222–225. 2347858

[286] V. Van den Eynde , P. K. Gillman , and B. B. Blackwell , “The Prescriber's Guide to the MAOI Diet‐Thinking through Tyramine Troubles,” Psychopharmacology Bulletin 52, no. 2 (2022): 73–116. 35721816 10.64719/pb.4434 PMC9172554

[287] E. Garcia and C. Santos , “Monoamine Oxidase Inhibitor Toxicity,” StatPearls (StatPearls Publishing. Copyright © 2025, StatPearls Publishing LLC., 2025). 29083800

[288] S. C. Dilsaver , “Heterocyclic Antidepressant, Monoamine Oxidase Inhibitor and Neuroleptic Withdrawal Phenomena,” Progress in Neuro‐Psychopharmacology & Biological Psychiatry 14, no. 2 (1990): 137–161. 1968671 10.1016/0278-5846(90)90097-z

[289] M. V. Vargas , L. E. Dunlap , C. Dong , et al., “Psychedelics Promote Neuroplasticity Through the Activation of Intracellular 5‐HT2A Receptors,” Science 379, no. 6633 (2023): 700–706. 36795823 10.1126/science.adf0435 PMC10108900

[290] A. Zięba , P. Stępnicki , D. Matosiuk , and A A. Kaczor , “Overcoming Depression With 5‐HT(2A) Receptor Ligands,” International Journal of Molecular Sciences 23, no. 1 (2021): 10. 35008436 10.3390/ijms23010010 PMC8744644

[291] D. E. Nichols , “Psychedelics,” Pharmacological Reviews 68, no. 2 (2016): 264–355. 26841800 10.1124/pr.115.011478 PMC4813425

[292] K. H. Preller , J. B. Burt , J. L. Ji , et al., “Changes in Global and Thalamic Brain Connectivity in LSD‐induced Altered States of Consciousness Are Attributable to the 5‐HT2A Receptor,” Elife 7 (2018): e35082. 30355445 10.7554/eLife.35082 PMC6202055

[293] B. M. Smith , J. M. Smith , J. H. Tsai , et al., “Discovery and Structure‐activity Relationship of (1R)‐8‐chloro‐2,3,4,5‐tetrahydro‐1‐methyl‐1H‐3‐benzazepine (Lorcaserin), a Selective Serotonin 5‐HT2C Receptor Agonist for the Treatment of Obesity,” Journal of Medicinal Chemistry 51, no. 2 (2008): 305–313. 18095642 10.1021/jm0709034

[294] C. T. Nguyen , S. Zhou , W. Shanahan , and R. Fain , “Lorcaserin in Obese and Overweight Patients Taking Prohibited Serotonergic Agents: A Retrospective Analysis,” Clinical Therapeutics 38, no. 6 (2016): 1498–1509. 27206567 10.1016/j.clinthera.2016.04.004

[295] S. Aznar and S. Hervig Mel , “The 5‐HT2A Serotonin Receptor in Executive Function: Implications for Neuropsychiatric and Neurodegenerative Diseases,” Neuroscience and Biobehavioral Reviews 64 (2016): 63–82. 26891819 10.1016/j.neubiorev.2016.02.008

[296] L. Olajossy‐Hilkesberger , B. Godlewska , A. Schosser‐Haupt , et al., “Polymorphisms of the 5‐HT2A Receptor Gene and Clinical Response to Olanzapine in Paranoid Schizophrenia,” Neuropsychobiology 64, no. 4 (2011): 202–210. 21912188 10.1159/000327602

[297] S. A. Gaitonde , C. Avet , M. de la Fuente Revenga , et al., “Pharmacological Fingerprint of Antipsychotic Drugs at the Serotonin 5‐HT(2A) Receptor,” Molecular Psychiatry 29, no. 9 (2024): 2753–2764. 38561467 10.1038/s41380-024-02531-7 PMC11420065

[298] H. Hashizume , M. Kawakami , M. Yoshida , M. Okada , Y. Enyo , and Y. Inomata , “Sarpogrelate Hydrochloride, a 5‐HT2A Receptor Antagonist, Attenuates Neurogenic Pain Induced by Nucleus Pulposus in Rats,” Spine (Phila Pa 1976) 32, no. 3 (2007): 315–320. 17268262 10.1097/01.brs.0000253601.35732.c1

[299] T. Nagatomo , M. Rashid , H. Muntasir , T. Komiyama , and H. Abul Muntasir , “Functions of 5‐HT2A Receptor and Its Antagonists in the Cardiovascular System,” Pharmacology & Therapeutics 104, no. 1 (2004): 59–81. 15500909 10.1016/j.pharmthera.2004.08.005

[300] Y. Gozes , M. Moayeri , J. F. Wiggins , and S H. Leppla , “Anthrax Lethal Toxin Induces Ketotifen‐sensitive Intradermal Vascular Leakage in Certain Inbred Mice,” Infection and Immunity 74, no. 2 (2006): 1266–1272. 16428776 10.1128/IAI.74.2.1266-1272.2006 PMC1360369

[301] M. Idzko , E. Panther , C. Stratz , et al., “The Serotoninergic Receptors of human Dendritic Cells: Identification and Coupling to Cytokine Release,” Journal of Immunology 172, no. 10 (2004): 6011–6019. 10.4049/jimmunol.172.10.6011 15128784

[302] C. Nieto , I. Rayo , L. de , et al., “Serotonin (5‐HT) Shapes the Macrophage Gene Profile Through the 5‐HT(2B)‐Dependent Activation of the Aryl Hydrocarbon Receptor,” Journal of Immunology 204, no. 10 (2020): 2808–2817. 10.4049/jimmunol.1901531 32253244

[303] A. J. Thompson and S C. Lummis , “The 5‐HT3 Receptor as a Therapeutic Target,” Expert Opinion on Therapeutic Targets 11, no. 4 (2007): 527–540. 17373882 10.1517/14728222.11.4.527 PMC1994432

[304] T K. Machu , “Therapeutics of 5‐HT3 Receptor Antagonists: Current Uses and Future Directions,” Pharmacology & Therapeutics 130, no. 3 (2011): 338–347. 21356241 10.1016/j.pharmthera.2011.02.003 PMC3103470

[305] G. Fakhfouri , R. Rahimian , J. E. Ghia , W. I. Khan , and A R. Dehpour , “Impact of 5‐HT_3_ Receptor Antagonists on Peripheral and central Diseases,” Drug Discovery Today 17, no. 13‐14 (2012): 741–747. 22390946 10.1016/j.drudis.2012.02.009

[306] T. J. Gan , K. G. Belani , S. Bergese , et al., “Fourth Consensus Guidelines for the Management of Postoperative Nausea and Vomiting,” Anesthesia and Analgesia 131, no. 2 (2020): 411–448. 32467512 10.1213/ANE.0000000000004833

[307] K. Gupta , R. Walton , and S P. Kataria , “Chemotherapy‐Induced Nausea and Vomiting: Pathogenesis, Recommendations, and New Trends,” Cancer Treatment and Research Communications 26 (2021): 100278. 33360668 10.1016/j.ctarc.2020.100278

[308] I. Butt and F. Kasmin , “Alosetron,” StatPearls (StatPearls Publishing Copyright © 2025, StatPearls Publishing LLC., 2025). 31971721

[309] C. J. Li , L. G. Zhang , L. B. Liu , et al., “Inhibition of Spinal 5‐HT3 Receptor and Spinal Dorsal Horn Neuronal Excitability Alleviates Hyperalgesia in a Rat Model of Parkinson's Disease,” Molecular Neurobiology 59, no. 12 (2022): 7253–7264. 36168076 10.1007/s12035-022-03034-8

[310] N. Eissazade , H. Mosavari , S. Eghdami , M. Boroon , F. Ashrafi , and M. Shalbafan , “Efficacy and Safety of 5‐hydroxytryptamine‐3 (5‐HT3) Receptor Antagonists in Augmentation With Selective Serotonin Reuptake Inhibitors (SSRIs) in the Treatment of Moderate to Severe Obsessive‐compulsive Disorder: A Systematic Review and Meta‐analysis of Randomized Clinical Trials,” Scientific Reports 13, no. 1 (2023): 20837. 38012263 10.1038/s41598-023-47931-x PMC10682036

[311] N. A. Khan and J P. Poisson , “5‐HT3 receptor‐channels Coupled With Na+ Influx in human T Cells: Role in T Cell Activation,” Journal of Neuroimmunology 99, no. 1 (1999): 53–60. 10496177 10.1016/s0165-5728(99)00101-0

[312] J. Ailani , R. C. Burch , and M S. Robbins , “The American Headache Society Consensus Statement: Update on Integrating New Migraine Treatments Into Clinical Practice,” Headache 61, no. 7 (2021): 1021–1039. 34160823 10.1111/head.14153

[313] C. P. Zhu , S. Q. Liu , K. Q. Wang , et al., “Targeting 5‐Hydroxytryptamine Receptor 1A in the Portal Vein to Decrease Portal Hypertension,” Gastroenterology 167, no. 5 (2024): 993–1007. 38906512 10.1053/j.gastro.2024.06.007

[314] J. Staroń , R. Bugno , A. S. Hogendorf , and A J. Bojarski , “5‐HT1A receptor Ligands and Their Therapeutic Applications: Review of New Patents,” Expert Opinion on Therapeutic Patents 28, no. 9 (2018): 679–689. 30124346 10.1080/13543776.2018.1514011

[315] A. Kaur Gill , Y. Bansal , R. Bhandari , et al., “Gepirone Hydrochloride: A Novel Antidepressant With 5‐HT1A Agonistic Properties,” Drugs of Today (Barcelona, Spain: 1998) 55, no. 7 (2019): 423–437. 31347611 10.1358/dot.2019.55.7.2958474

[316] L. A. Dawson and J M. Watson , “Vilazodone: A 5‐HT1A Receptor Agonist/Serotonin Transporter Inhibitor for the Treatment of Affective Disorders,” CNS neuroscience & therapeutics 15, no. 2 (2009): 107–117. 19499624 10.1111/j.1755-5949.2008.00067.x PMC6493994

[317] Z. T. Sahli , P. Banerjee , and F I. Tarazi , “The Preclinical and Clinical Effects of Vilazodone for the Treatment of Major Depressive Disorder,” Expert Opin Drug Discov 11, no. 5 (2016): 515–523. 26971593 10.1517/17460441.2016.1160051 PMC4841022

[318] N. M. Bozkurt and G. Unal , “Vortioxetine Improved Negative and Cognitive Symptoms of Schizophrenia in Subchronic MK‐801 Model in Rats,” Behavioural Brain Research 444 (2023): 114365. 36858318 10.1016/j.bbr.2023.114365

[319] S. Nikolaus , H. J. Wittsack , M. Beu , et al., “The 5‐HT(1A) Receptor Antagonist WAY‐100635 Decreases Motor/Exploratory Behaviors and Nigrostriatal and Mesolimbocortical Dopamine D(2/3) Receptor Binding in Adult Rats,” Pharmacology Biochemistry and Behavior 215 (2022): 173363. 35227734 10.1016/j.pbb.2022.173363

[320] H. C. Diener and A. May , “Drug Treatment of Cluster Headache,” Drugs 82, no. 1 (2022): 33–42. 34919214 10.1007/s40265-021-01658-z PMC8748342

[321] P. J. Pauwels and G W. John , “Present and Future of 5‐HT Receptor Agonists as Antimigraine Drugs,” Clinical Neuropharmacology 22, no. 3 (1999): 123–136. 10367177

[322] C. Cameron , S. Kelly , S. C. Hsieh , et al., “Triptans in the Acute Treatment of Migraine: A Systematic Review and Network Meta‐Analysis,” Headache 55, no. Suppl 4 (2015): 221–235. 26178694 10.1111/head.12601

[323] M. Tanaka , N. Török , and L. Vécsei , “Are 5‐HT(1) Receptor Agonists Effective Anti‐migraine Drugs?” Expert Opinion on Pharmacotherapy 22, no. 10 (2021): 1221–1225. 33843394 10.1080/14656566.2021.1910235

[324] D. Xue , X. Guo , J. Liu , et al., “Tryptophan‐rich Diet and Its Effects on Htr7(+) Tregs in Alleviating Neuroinflammation and Cognitive Impairment Induced by Lipopolysaccharide,” Journal of Neuroinflammation 21, no. 1 (2024): 241. 39334486 10.1186/s12974-024-03239-9 PMC11437714

[325] Y N. Lamb , “Lasmiditan: First Approval,” Drugs 79, no. 18 (2019): 1989–1996. 31749059 10.1007/s40265-019-01225-7

[326] R. Ataee , S. Ajdary , M. Zarrindast , M. Rezayat , and M R. Hayatbakhsh , “Anti‐mitogenic and Apoptotic Effects of 5‐HT1B Receptor Antagonist on HT29 Colorectal Cancer Cell Line,” Journal of Cancer Research and Clinical Oncology 136, no. 10 (2010): 1461–1469. 20306273 10.1007/s00432-010-0801-3 PMC11827785

[327] H. Hagena and D. Manahan‐Vaughan , “The Serotonergic 5‐HT4 Receptor: A Unique Modulator of Hippocampal Synaptic Information Processing and Cognition,” Neurobiology of Learning and Memory 138 (2017): 145–153. 27317942 10.1016/j.nlm.2016.06.014

[328] Serotonin 5‐HT4 Receptor Agonists . LiverTox: Clinical and Research Information on Drug‐Induced Liver Injury. National Institute of Diabetes and Digestive and Kidney Diseases; 2012. 31643176

[329] W. L. Hasler and P. Schoenfeld , “Safety Profile of tegaserod, a 5‐HT4 Receptor Agonist, for the Treatment of Irritable Bowel Syndrome,” Drug Safety 27, no. 9 (2004): 619–631. 15230644 10.2165/00002018-200427090-00001

[330] S. J. Hwang , J. H. Wang , J. S. Lee , et al., “Yeokwisan, a Standardized Herbal Formula, Enhances Gastric Emptying via Modulation of the Ghrelin Pathway in a Loperamide‐induced Functional Dyspepsia Mouse Model,” Frontiers in pharmacology 12 (2021): 753153. 34630123 10.3389/fphar.2021.753153 PMC8493126

[331] K. Conlon , J. H. De Maeyer , C. Bruce , et al., “Nonclinical Cardiovascular Studies of Prucalopride, a Highly Selective 5‐Hydroxytryptamine 4 Receptor Agonist,” Journal of Pharmacology and Experimental Therapeutics 364, no. 2 (2018): 156–169. 29180358 10.1124/jpet.117.244079

[332] F. Darcet , A. M. Gardier , D. J. David , and J P. Guilloux , “Chronic 5‐HT4 Receptor Agonist Treatment Restores Learning and Memory Deficits in a Neuroendocrine Mouse Model of Anxiety/Depression,” Neuroscience Letters 616 (2016): 197–203. 26850572 10.1016/j.neulet.2016.01.055

[333] N. Kozono , A. Ohtani , and T. Shiga , “Roles of the Serotonin 5‐HT4 Receptor in Dendrite Formation of the Rat Hippocampal Neurons in Vitro,” Brain Research 1655 (2017): 114–121. 27894797 10.1016/j.brainres.2016.11.021

[334] V. H. Dam , K. Köhler‐Forsberg , B. Ozenne , et al., “Effect of Antidepressant Treatment on 5‐HT(4) Receptor Binding and Associations with Clinical Outcomes and Verbal Memory in Major Depressive Disorder,” Biological Psychiatry 97, no. 3 (2025): 261–268. 39181386 10.1016/j.biopsych.2024.08.009

[335] M. Yamazaki , N. Yamamoto , J. Yarimizu , et al., “Functional Mechanism of ASP5736, a Selective Serotonin 5‐HT(5A) Receptor Antagonist With Potential Utility for the Treatment of Cognitive Dysfunction in Schizophrenia,” European Neuropsychopharmacology 28, no. 5 (2018): 620–629. 29571967 10.1016/j.euroneuro.2018.03.003

[336] J. Lalut , D. Karila , P. Dallemagne , and C. Rochais , “Modulating 5‐HT(4) and 5‐HT(6) Receptors in Alzheimer's Disease Treatment,” Future Medicinal Chemistry 9, no. 8 (2017): 781–795. 28504917 10.4155/fmc-2017-0031

[337] R. Khoury , N. Grysman , J. Gold , K. Patel , and G T. Grossberg , “The Role of 5 HT6‐receptor Antagonists in Alzheimer's Disease: An Update,” Expert Opinion on Investigational Drugs 27, no. 6 (2018): 523–533. 29848076 10.1080/13543784.2018.1483334

[338] R. Nirogi , R. Abraham , V. Benade , et al., “SUVN‐502, a Novel, Potent, Pure, and Orally Active 5‐HT6 Receptor Antagonist: Pharmacological, Behavioral, and Neurochemical Characterization,” Behavioural Pharmacology 30, no. 1 (2019): 16–35. 29847336 10.1097/FBP.0000000000000414

[339] R. Nirogi , P. Jayarajan , A. Shinde , et al., “Progress in Investigational Agents Targeting Serotonin‐6 Receptors for the Treatment of Brain Disorders,” Biomolecules 13, no. 2 (2023): 309. 36830678 10.3390/biom13020309 PMC9953539

[340] A. Meneses , G. Perez‐Garcia , G. Liy‐Salmeron , T. Ponce‐López , E. Lacivita , and M. Leopoldo , “5‐HT7 receptor Activation: Procognitive and Antiamnesic Effects,” Psychopharmacology 232, no. 3 (2015): 595–603. 25074446 10.1007/s00213-014-3693-0

[341] M. Leon‐Ponte , G. P. Ahern , and P. J. O'Connell , “Serotonin Provides an Accessory Signal to Enhance T‐cell Activation by Signaling Through the 5‐HT7 Receptor,” Blood 109, no. 8 (2007): 3139–3146. 17158224 10.1182/blood-2006-10-052787 PMC1852236

[342] O. Mnie‐Filali , L. Lambás‐Señas , L. Zimmer , and N. Haddjeri , “5‐HT7 receptor Antagonists as a New Class of Antidepressants,” Drug News & Perspectives 20, no. 10 (2007): 613–618. 18301795 10.1358/dnp.2007.20.10.1181354

[343] B. Sahin , E. Ozdemir , E. Gumus , M. Ergul , and A S. Taskiran , “The 5‐HT7 Receptor Antagonist SB‐269970 Alleviates Seizure Activity and Downregulates Hippocampal c‐Fos Expression in Pentylenetetrazole‐induced Kindled Rats,” Neurological Research 44, no. 9 (2022): 786–796. 35404776 10.1080/01616412.2022.2064700

[344] P. B. Hedlund , S. Huitron‐Resendiz , S. J. Henriksen , and J G. Sutcliffe , “5‐HT7 receptor Inhibition and Inactivation Induce Antidepressantlike Behavior and Sleep Pattern,” Biological Psychiatry 58, no. 10 (2005): 831–837. 16018977 10.1016/j.biopsych.2005.05.012

[345] M. Padelli , C. Bruno , J. Lemarchand , et al., “[Determination of thresholds values for platelet serotonin and urinary 5‐HIAA concentrations for the biological diagnosis of digestive neuroendocrine tumors],” Annales de Biologie Clinique (Paris) 77, no. 2 (2019): 161–168. Quelles valeurs de référence pour la concentration de la sérotonine plaquettaire et du 5‐HIAA urinaire pour le diagnostic des tumeurs neuroendocrines digestives ?. 10.1684/abc.2019.1432 30998196

[346] D. Ye , H. Xu , Q. Tang , H. Xia , C. Zhang , and F. Bi , “The Role of 5‐HT Metabolism in Cancer,” Biochimica et Biophysica Acta (BBA) ‐ Reviews on Cancer 1876, no. 2 (2021): 188618. 34428515 10.1016/j.bbcan.2021.188618

[347] S. Karmakar and G. Lal , “Role of Serotonin Receptor Signaling in Cancer Cells and Anti‐tumor Immunity,” Theranostics 11, no. 11 (2021): 5296–5312. 33859748 10.7150/thno.55986 PMC8039959

[348] A. Chiechi , C. Novello , G. Magagnoli , et al., “Elevated TNFR1 and Serotonin in Bone Metastasis Are Correlated With Poor Survival Following Bone Metastasis Diagnosis for both Carcinoma and Sarcoma Primary Tumors,” Clinical Cancer Research 19, no. 9 (2013): 2473–2485. 23493346 10.1158/1078-0432.CCR-12-3416 PMC3644003

[349] O. Casar‐Borota , J. Botling , D. Granberg , et al., “Serotonin, ATRX, and DAXX Expression in Pituitary Adenomas: Markers in the Differential Diagnosis of Neuroendocrine Tumors of the Sellar Region,” American Journal of Surgical Pathology 41, no. 9 (2017): 1238–1246. 28719461 10.1097/PAS.0000000000000908

[350] A. Nocito , F. Dahm , W. Jochum , et al., “Serotonin Regulates Macrophage‐mediated Angiogenesis in a Mouse Model of Colon Cancer Allografts,” Cancer Research 68, no. 13 (2008): 5152–5158. 18593914 10.1158/0008-5472.CAN-08-0202

[351] J. Y. Sakita , M. Bader , E. S. Santos , et al., “Serotonin Synthesis Protects the Mouse Colonic Crypt From DNA Damage and Colorectal Tumorigenesis,” Journal of Pathology 249, no. 1 (2019): 102–113. 31038736 10.1002/path.5285

[352] T. Li , B. Fu , X. Zhang , et al., “Overproduction of Gastrointestinal 5‐HT Promotes Colitis‐Associated Colorectal Cancer Progression via Enhancing NLRP3 Inflammasome Activation,” Cancer immunology research 9, no. 9 (2021): 1008–1023. 34285037 10.1158/2326-6066.CIR-20-1043

[353] T. Depoilly , R. Leroux , D. Andrade , et al., “Immunophenotypic and Molecular Characterization of Pancreatic Neuroendocrine Tumors Producing Serotonin,” Modern Pathology 35, no. 11 (2022): 1713–1722. 35739266 10.1038/s41379-022-01110-x

[354] A. Fischer , H. S. Rennert , and G. Rennert , “Selective Serotonin Reuptake Inhibitors Associated With Increased Mortality Risk in Breast Cancer Patients in Northern Israel,” International Journal of Epidemiology 51, no. 3 (2022): 807–816. 35134960 10.1093/ije/dyac004

[355] S. W. Wesmiller , C. M. Bender , S. M. Sereika , et al., “Association Between Serotonin Transport Polymorphisms and Postdischarge Nausea and Vomiting in Women Following Breast Cancer Surgery,” Oncology Nursing Forum 41, no. 2 (2014): 195–202. 24578078 10.1188/14.ONF.195-202 PMC4023462

[356] R. Gärtner , D. Cronin‐Fenton , H. H. Hundborg , et al., “Use of Selective Serotonin Reuptake Inhibitors and Risk of Re‐operation due to Post‐surgical Bleeding in Breast Cancer Patients: A Danish Population‐based Cohort Study,” BMC Surgery [Electronic Resource] 10 (2010): 3. 20096133 10.1186/1471-2482-10-3 PMC2823600

[357] L. Grassi , E. Rossi , M. Cobianchi , et al., “Depression and Serotonin Transporter (5‐HTTLPR) Polymorphism in Breast Cancer Patients,” Journal of Affective Disorders 124, no. 3 (2010): 346–350. 20122741 10.1016/j.jad.2009.12.022

[358] T. Ling , Z. Dai , H. Wang , et al., “Serotonylation in Tumor‐associated Fibroblasts Contributes to the Tumor‐promoting Roles of Serotonin in Colorectal Cancer,” Cancer Letters 600 (2024): 217150. 39097134 10.1016/j.canlet.2024.217150

[359] H. Yu , T. Qu , J. Yang , and Q. Dai , “Serotonin Acts Through YAP to Promote Cell Proliferation: Mechanism and Implication in Colorectal Cancer Progression,” Cell Communication and Signaling 21, no. 1 (2023): 75. 37046308 10.1186/s12964-023-01096-2 PMC10100184

[360] P. Zhu , T. Lu , Z. Chen , et al., “5‐hydroxytryptamine Produced by Enteric Serotonergic Neurons Initiates Colorectal Cancer Stem Cell Self‐renewal and Tumorigenesis,” Neuron 110, no. 14 (2022): 2268–2282. e4. 35550066 10.1016/j.neuron.2022.04.024

[361] F. Mamdouh , S. Abdel Alem , and M. Abdo , “Serum Serotonin as a Potential Diagnostic Marker for Hepatocellular Carcinoma,” Journal of Interferon & Cytokine Research 39, no. 12 (2019): 780–785. 31478787 10.1089/jir.2019.0088

[362] R. Iwase , H. Shiba , T. Gocho , et al., “Syndrome of Inappropriate Secretion of Antidiuretic Hormone Due to Selective Serotonin Reuptake Inhibitors After Pancreaticoduodenectomy for Carcinoma of the Ampulla of Vater: Case Report,” International Surgery 98, no. 4 (2013): 289–291. 24229010 10.9738/INTSURG-D-13-00032.1 PMC3829050

[363] A. Serafeim , G. Grafton , A. Chamba , et al., “5‐Hydroxytryptamine Drives Apoptosis in Biopsylike Burkitt Lymphoma Cells: Reversal by Selective Serotonin Reuptake Inhibitors,” Blood 99, no. 7 (2002): 2545–2553. 11895792 10.1182/blood.v99.7.2545

[364] K. Liu , Y. Zhang , G. Du , et al., “5‐HT Orchestrates Histone Serotonylation and Citrullination to Drive Neutrophil Extracellular Traps and Liver Metastasis,” Journal of Clinical Investigation 135, no. 8 (2025): e183544. 39903533 10.1172/JCI183544 PMC11996869

[365] D G. Tang , “Serotonin Sets up Neutrophil Extracellular Traps to Promote Neuroendocrine Prostate Cancer Metastasis in the Liver,” Journal of Clinical Investigation 135, no. 8 (2025): e191687. 40231471 10.1172/JCI191687 PMC11996856

[366] L. Yin , J. Li , J. Wang , et al., “MAOA Promotes Prostate Cancer Cell Perineural Invasion Through SEMA3C/PlexinA2/NRP1‐cMET Signaling,” Oncogene 40, no. 7 (2021): 1362–1374. 33420365 10.1038/s41388-020-01615-2 PMC8604374

[367] X. G. Yang , Y. Y. Li , D. X. Zhao , et al., “Repurposing of a Monoamine Oxidase A Inhibitor‑Heptamethine Carbocyanine Dye Conjugate for Paclitaxel‑Resistant Non‑Small Cell Lung Cancer,” Oncology Reports 45, no. 3 (2021): 1306–1314. 33650669 10.3892/or.2021.7950

[368] B. Huang , Z. Zhou , J. Liu , et al., “The Role of Monoamine Oxidase A in HPV‐16 E7‐induced Epithelial‐mesenchymal Transition and HIF‐1alpha Protein Accumulation in Non‐small Cell Lung Cancer Cells,” International Journal of Biological Sciences 16, no. 14 (2020): 2692–2703. 32792865 10.7150/ijbs.46966 PMC7415426

[369] V. C. Chen , M. J. Lee , Y. H. Yang , M. L. Lu , W. C. Chiu , and M E. Dewey , “Selective Serotonin Reuptake Inhibitors Use and Hepatocellular Carcinoma in Patients With Alcohol Use Disorder,” Drug and Alcohol Dependence 219 (2021): 108495. 33429293 10.1016/j.drugalcdep.2020.108495

[370] N. Zhang , J. Sundquist , K. Sundquist , Z. G. Zhang , and J. Ji , “Combined Use of Aspirin and Selective Serotonin Reuptake Inhibitors Is Associated with Lower Risk of Colorectal Cancer: A Nested Case‐Control Study,” American Journal of Gastroenterology 116, no. 6 (2021): 1313–1321. 33661146 10.14309/ajg.0000000000001192

[371] V. Kannen , H. Hintzsche , D. L. Zanette , et al., “Antiproliferative Effects of Fluoxetine on Colon Cancer Cells and in a Colonic Carcinogen Mouse Model,” PLoS ONE 7, no. 11 (2012): e50043. 23209640 10.1371/journal.pone.0050043 PMC3507893

[372] V. Kannen , S. B. Garcia , and W. A. Silva Jr. , “Oncostatic Effects of Fluoxetine in Experimental Colon Cancer Models,” Cell Signalling 27, no. 9 (2015): 1781–1788. 26004136 10.1016/j.cellsig.2015.05.008

[373] B. Grygier , B. Arteta , M. Kubera , et al., “Inhibitory Effect of Antidepressants on B16F10 Melanoma Tumor Growth,” Pharmacology Reports 65, no. 3 (2013): 672–681. 10.1016/s1734-1140(13)71045-4 23950590

[374] M. E. Di Rosso , H. A. Sterle , G. A. Cremaschi , and A M. Genaro , “Beneficial Effect of Fluoxetine and Sertraline on Chronic Stress‐Induced Tumor Growth and Cell Dissemination in a Mouse Model of Lymphoma: Crucial Role of Antitumor Immunity,” Frontiers in immunology 9 (2018): 1341. 29971064 10.3389/fimmu.2018.01341 PMC6018164

[375] J. Li , B. Mei , L. Feng , et al., “Amitriptyline Revitalizes ICB Response via Dually Inhibiting Kyn/Indole and 5‐HT Pathways of Tryptophan Metabolism in Ovarian Cancer,” Iscience 27, no. 12 (2024): 111488. 39759009 10.1016/j.isci.2024.111488 PMC11697709

[376] J. Li , T. Pu , L. Yin , Q. Li , C. P. Liao , and B J. Wu , “MAOA‐mediated Reprogramming of Stromal Fibroblasts Promotes Prostate Tumorigenesis and Cancer Stemness,” Oncogene 39, no. 16 (2020): 3305–3321. 32066880 10.1038/s41388-020-1217-4

[377] J. Wei , L. Yin , J. Li , et al., “Bidirectional Cross‐talk Between MAOA and AR Promotes Hormone‐Dependent and Castration‐Resistant Prostate Cancer,” Cancer Research 81, no. 16 (2021): 4275–4289. 34167949 10.1158/0008-5472.CAN-21-0198 PMC8373824

[378] N. Gurbuz , A. A. Ashour , S. N. Alpay , and B. Ozpolat , “Down‐regulation of 5‐HT1B and 5‐HT1D Receptors Inhibits Proliferation, Clonogenicity and Invasion of human Pancreatic Cancer Cells,” PLoS ONE 9, no. 8 (2014): e105245. 25170871 10.1371/journal.pone.0105245 PMC4149367

[379] Y. Liu , H. Zhang , Z. Wang , P. Wu , and W. Gong , “5‐Hydroxytryptamine1a receptors on Tumour Cells Induce Immune Evasion in Lung Adenocarcinoma Patients With Depression via Autophagy/pSTAT3,” European Journal of Cancer 114 (2019): 8–24. 31009821 10.1016/j.ejca.2019.03.017

[380] X. Zuo , Z. Chen , J. Cai , et al., “5‐Hydroxytryptamine Receptor 1D Aggravates Hepatocellular Carcinoma Progression through FoxO6 in AKT‐Dependent and Independent Manners,” Hepatology 69, no. 5 (2019): 2031–2047. 30561038 10.1002/hep.30430

[381] L. Mao , F. Xin , J. Ren , et al., “5‐HT2B‐mediated Serotonin Activation in Enterocytes Suppresses Colitis‐associated Cancer Initiation and Promotes Cancer Progression,” Theranostics 12, no. 8 (2022): 3928–3945. 35664068 10.7150/thno.70762 PMC9131283

[382] F. Abedini , O. Amjadi , A. Hedayatizadeh‐Omran , S. A. Lira , and G. Ahangari , “Serotonin Receptors and Acetylcholinesterase Gene Expression Alternations: The Potential Value on Tumor Microenvironment of Gastric Cancer,” Oncology 101, no. 7 (2023): 415–424. 37231904 10.1159/000530878

[383] I. Drozdov , M. Kidd , B. I. Gustafsson , et al., “Autoregulatory Effects of Serotonin on Proliferation and Signaling Pathways in Lung and Small Intestine Neuroendocrine Tumor Cell Lines,” Cancer 115, no. 21 (2009): 4934–4945. 19634160 10.1002/cncr.24533 PMC2767432

[384] T. Oufkir and C. Vaillancourt , “Phosphorylation of JAK2 by Serotonin 5‐HT (2A) Receptor Activates both STAT3 and ERK1/2 Pathways and Increases Growth of JEG‐3 human Placental Choriocarcinoma Cell,” Placenta 32, no. 12 (2011): 1033–1040. 21993263 10.1016/j.placenta.2011.09.005

[385] J. Tang , Z. Wang , J. Liu , C. Zhou , and J. Chen , “Downregulation of 5‐hydroxytryptamine Receptor 3A Expression Exerts an Anticancer Activity Against Cell Growth in Colorectal Carcinoma Cells in Vitro,” Oncology letters 16, no. 5 (2018): 6100–6108. 30405756 10.3892/ol.2018.9351 PMC6202457

[386] G. W. Huang , Q. Q. Chen , C. C. Ma , L. H. Xie , and J. Gu , “linc01305 promotes Metastasis and Proliferation of Esophageal Squamous Cell Carcinoma Through Interacting With IGF2BP2 and IGF2BP3 to Stabilize HTR3A mRNA,” International Journal of Biochemistry & Cell Biology 136 (2021): 106015. 34022433 10.1016/j.biocel.2021.106015

[387] A. Stewart , P. A. Davies , E. F. Kirkness , P. Safa , and T G. Hales , “Introduction of the 5‐HT3B Subunit Alters the Functional Properties of 5‐HT3 Receptors Native to Neuroblastoma Cells,” Neuropharmacology 44, no. 2 (2003): 214–223. 12623220 10.1016/s0028-3908(02)00376-3

[388] A. Barzegar‐Fallah , H. Alimoradi , J. L. Dunlop , E. Torbati , and S K. Baird , “Serotonin Type‐3 Receptor Antagonists Selectively Kill Melanoma Cells Through Classical Apoptosis, Microtubule Depolymerisation, ERK Activation, and NF‐κB Downregulation,” Cell Biology and Toxicology (2021). 10.1007/s10565-021-09667-0 34654991

[389] J. R. Chen , M. S. Huang , Y. C. Lee , M. H. Lin , and Y F. Yang , “Potential Clinical Value of 5‐Hydroxytryptamine Receptor 3C as a Prognostic Biomarker for Lung Cancer,” Journal of oncology 2021 (2021): 1901191. 34868311 10.1155/2021/1901191 PMC8639264

[390] M. Itsumi , M. Shiota , Y. Sekino , et al., “High‐throughput Screen Identifies 5‐HT Receptor as a Modulator of AR and a Therapeutic Target for Prostate Cancer,” Prostate 80, no. 11 (2020): 885–894. 32483877 10.1002/pros.24022

[391] E. Louiset , K. Isvi , J. M. Gasc , et al., “Ectopic Expression of serotonin7 Receptors in an Adrenocortical Carcinoma co‐secreting Renin and Cortisol,” Endocrine‐Related Cancer 15, no. 4 (2008): 1025–1034. 18708508 10.1677/ERC-08-0085

[392] S. H. Hejazi , G. Ahangari , M. Pornour , et al., “Evaluation of Gene Expression Changes of Serotonin Receptors, 5‐HT3AR and 5‐HT2AR as Main Stress Factors in Breast Cancer Patients,” Asian Pacific Journal of Cancer Prevention 15, no. 11 (2014): 4455–4458. 24969868 10.7314/apjcp.2014.15.11.4455

[393] M. Kubera , M. Maes , G. Kenis , Y. K. Kim , and W. Lasoń , “Effects of Serotonin and Serotonergic Agonists and Antagonists on the Production of Tumor Necrosis Factor Alpha and Interleukin‐6,” Psychiatry Research 134, no. 3 (2005): 251–258. 15892984 10.1016/j.psychres.2004.01.014

[394] B. Yu , J. Becnel , M. Zerfaoui , R. Rohatgi , A. H. Boulares , and C D. Nichols , “Serotonin 5‐hydroxytryptamine(2A) Receptor Activation Suppresses Tumor Necrosis Factor‐alpha‐induced Inflammation With Extraordinary Potency,” Journal of Pharmacology and Experimental Therapeutics 327, no. 2 (2008): 316–323. 18708586 10.1124/jpet.108.143461

[395] B. Shu , M. Zhai , X. Miao , et al., “Serotonin and YAP/VGLL4 Balance Correlated With Progression and Poor Prognosis of Hepatocellular Carcinoma,” Scientific Reports 8, no. 1 (2018): 9739. 29950605 10.1038/s41598-018-28075-9 PMC6021381

[396] S. Liu , M. Zhai , and W. Xiao , “Intra‐platelet Serotonin and YAP Contributed to Poor Prognosis of Hepatocellular Carcinoma,” Life Sciences 270 (2021): 119140. 33524420 10.1016/j.lfs.2021.119140

[397] P. K. Kopparapu , M. Tinzl , L. Anagnostaki , J. L. Persson , and N. Dizeyi , “Expression and Localization of Serotonin Receptors in human Breast Cancer,” Anticancer Research 33, no. 2 (2013): 363–370. 23393325

[398] C. Soll , J. H. Jang , M. O. Riener , et al., “Serotonin Promotes Tumor Growth in human Hepatocellular Cancer,” Hepatology 51, no. 4 (2010): 1244–1254. 20099302 10.1002/hep.23441

[399] G G. Xiao , “Targeting Serotonin System in Pancreatic Cancer,” Pancreas 49, no. 1 (2020): e1. 10.1097/MPA.0000000000001417 31856091

[400] G. Le‐Bel , M. Benhassine , S. Landreville , and S L. Guerin , “Analysis of the Proteasome Activity and the Turnover of the Serotonin Receptor 2B (HTR2B) in human Uveal Melanoma,” Experimental Eye Research 184 (2019): 72–77. 31002821 10.1016/j.exer.2019.04.013

[401] T. Li , L. Wei , X. Zhang , et al., “Serotonin Receptor HTR2B Facilitates Colorectal Cancer Metastasis via CREB1‐ZEB1 Axis‐Mediated Epithelial‐Mesenchymal Transition,” Molecular Cancer Research 22, no. 6 (2024): 538–554. 38381131 10.1158/1541-7786.MCR-23-0513

[402] M. A. M. Peters , C. Meijer , R. S. N. Fehrmann , et al., “Serotonin and Dopamine Receptor Expression in Solid Tumours Including Rare Cancers,” Pathology Oncology Research 26, no. 3 (2020): 1539–1547. 31478179 10.1007/s12253-019-00734-w PMC7297821

[403] S. H. Hejazi , G. Ahangari , and A. Deezagi , “Alternative Viewpoint against Breast Cancer Based on Selective Serotonin Receptors 5HTR3A and 5HTR2A Antagonists That Can Mediate Apoptosis in MCF‐7 Cell Line,” Current Drug Discovery Technologies 12, no. 4 (2015): 240–249. 26768715 10.2174/1570163813666151126215210

[404] B. Sonier , M. Arseneault , C. Lavigne , R. J. Ouellette , and C. Vaillancourt , “The 5‐HT2A Serotoninergic Receptor Is Expressed in the MCF‐7 human Breast Cancer Cell Line and Reveals a Mitogenic Effect of Serotonin,” Biochemical and Biophysical Research Communications 343, no. 4 (2006): 1053–1059. 16580628 10.1016/j.bbrc.2006.03.080

[405] M. R. Ambrosio , E. Magli , G. Caliendo , et al., “Serotoninergic Receptor Ligands Improve Tamoxifen Effectiveness on Breast Cancer Cells,” BMC cancer 22, no. 1 (2022): 171. 35168555 10.1186/s12885-021-09147-y PMC8845285

[406] Y. Ren , G. Bao , H. Yang , Z. Cao , Z. Shao , and Y. Zhang , “Ethiadin Induces Apoptosis and Suppresses Growth of MCF‐7 Breast Cancer Cells by Regulating the Phosphorylation of Glycogen Synthase Kinase 3 Beta (GSK3beta),” Discovery Medicine 33, no. 169 (2022): 55–67. 36482736

[407] Q. E. Xie , X. Du , M. Wang , et al., “Identification of Serotonin as a Predictive Marker for Breast Cancer Patients,” International Journal of General Medicine 14 (2021): 1939–1948. 34045888 10.2147/IJGM.S310591 PMC8144847

[408] K. Muller , K. P. Gilbertz , and V. Meineke , “Serotonin and Ionizing Radiation Synergistically Affect Proliferation and Adhesion Molecule Expression of Malignant Melanoma Cells,” Journal of Dermatological Science 68, no. 2 (2012): 89–98. 22938911 10.1016/j.jdermsci.2012.08.001

[409] P. Eisenberg , J. Figueroa‐Vadillo , R. Zamora , et al., “Improved Prevention of Moderately Emetogenic Chemotherapy‐induced Nausea and Vomiting With palonosetron, a Pharmacologically Novel 5‐HT3 Receptor Antagonist: Results of a Phase III, Single‐dose Trial versus dolasetron,” Cancer 98, no. 11 (2003): 2473–2482. 14635083 10.1002/cncr.11817

[410] Y. Nitta , M. Nishibori , H. Iwagaki , et al., “Changes in Serotonin Dynamics in the Gastrointestinal Tract of Colon‐26 Tumour‐bearing Mice: Effects of Cisplatin Treatment,” Naunyn‐Schmiedebergs Archives of Pharmacology 364, no. 4 (2001): 329–334. 11683520 10.1007/s002100100461

[411] Z. Olfati , G. Rigi , H. Vaseghi , Z. Zamanzadeh , M. Sohrabi , and S H. Hejazi , “Evaluation of Serotonin Receptors (5HTR2A and 5HTR3A) mRNA Expression Changes in Tumor of Breast Cancer Patients,” Medical Journal of the Islamic Republic of Iran 34 (2020): 99. 33315977 10.34171/mjiri.34.99 PMC7722952

[412] R. Ataee , S. Ajdary , M. Rezayat , M. A. Shokrgozar , S. Shahriari , and M R. Zarrindast , “Study of 5HT3 and HT4 Receptor Expression in HT29 Cell Line and human Colon Adenocarcinoma Tissues,” Archives of Iranian Medicine 13, no. 2 (2010): 120–125. 20187666

[413] M. El‐Salhy and V. Dennerqvist , “Effects of Triple Therapy With Octreotide, Galanin and Serotonin on Liver Metastasis of human Colon Cancer in Xenografts,” Oncology Reports 11, no. 6 (2004): 117711–117782. 15138552

[414] J. H. Jun , J. E. Oh , J. K. Shim , Y. L. Kwak , and J S. Cho , “Effects of Bisphenol A on the Proliferation, Migration, and Tumor Growth of Colon Cancer Cells: In Vitro and in Vivo Evaluation With Mechanistic Insights Related to ERK and 5‐HT3,” Food and Chemical Toxicology 158 (2021): 112662. 34743013 10.1016/j.fct.2021.112662

[415] F. Del Bello , A. Bonifazi , G. Giorgioni , et al., “Chemical Manipulations on the 1,4‐dioxane Ring of 5‐HT(1A) Receptor Agonists Lead to Antagonists Endowed With Antitumor Activity in Prostate Cancer Cells,” European Journal of Medicinal Chemistry 168 (2019): 461–473. 30844609 10.1016/j.ejmech.2019.02.056

[416] E. J. Siddiqui , M. Shabbir , D. P. Mikhailidis , C. S. Thompson , and F H. Mumtaz , “The Role of Serotonin (5‐hydroxytryptamine1A and 1B) Receptors in Prostate Cancer Cell Proliferation,” Journal of Urology 176, no. 4 Pt 1 (2006): 1648–1653. 16952708 10.1016/j.juro.2006.06.087

[417] R. Henriksen , N. Dizeyi , and P A. Abrahamsson , “Expression of Serotonin Receptors 5‐HT1A, 5‐HT1B, 5‐HT2B and 5‐HT4 in Ovary and in Ovarian Tumours,” Anticancer Research 32, no. 4 (2012): 1361–1366. 22493371

[418] E. J. Siddiqui , M. A. Shabbir , D. P. Mikhailidis , F. H. Mumtaz , and C S. Thompson , “The Effect of Serotonin and Serotonin Antagonists on Bladder Cancer Cell Proliferation,” Bju International 97, no. 3 (2006): 634–639. 16469039 10.1111/j.1464-410X.2006.06056.x

[419] M. G. Cattaneo , E. Palazzi , G. Bondiolotti , and L M. Vicentini , “5‐HT1D receptor Type Is Involved in Stimulation of Cell Proliferation by Serotonin in human Small Cell Lung Carcinoma,” European Journal of Pharmacology 268, no. 3 (1994): 425–430. 7805767 10.1016/0922-4106(94)90068-x

[420] N. M. Abdel‐Hamid , D. E. Shehata , A. A. Abdel‐Ghany , A. Ragaa , and A. Wahid , “Serum Serotonin as Unexpected Potential Marker for Staging of Experimental Hepatocellular Carcinoma,” Biomedicine & Pharmacotherapy 83 (2016): 407–411. 27424322 10.1016/j.biopha.2016.07.005

[421] S. Niture , M. A. Gyamfi , H. Kedir , et al., “Serotonin Induced Hepatic Steatosis Is Associated With Modulation of Autophagy and Notch Signaling Pathway,” Cell Communication and Signaling 16, no. 1 (2018): 78. 30409162 10.1186/s12964-018-0282-6 PMC6225666

[422] K. A. Yagaloff , G. Lozano , T. V. Dyke , A. J. Levine , and P R. Hartig , “Serotonin 5‐{HTlc} Receptors Are Expressed at High Density on Choroid Plexus Tumors From Transgenic Mice,” Brain Research (1986): 6–6. 3022874 10.1016/0006-8993(86)91089-9

[423] V. Contesse , Y. Reznik , C. Duparc , et al., “Effects of Serotonin and Vasopressin on Cortisol Production From an Adrenocortical Tumor Causing Subclinical Cushing's Syndrome,” Endocrine Research 28, no. 4 (2002): 787–791. 12530699 10.1081/erc-120017074

[424] Y. Nakamura , K. Ise , Y. Yamazaki , F. Fujishima , K. M. McNamara , and H. Sasano , “Serotonin Receptor 4 (5‐hydroxytryptamine receptor Type 4) Regulates Expression of Estrogen Receptor Beta and Cell Migration in Hormone‐naive Prostate Cancer,” Indian Journal of Pathology & Microbiology 60, no. 1 (2017): 33–37. 28195088 10.4103/0377-4929.200022

[425] V. Cınar , Z. Hamurcu , A. Guler , N. Nurdinov , and B. Ozpolat , “Serotonin 5‐HT7 Receptor Is a Biomarker Poor Prognostic Factor and Induces Proliferation of Triple‐negative Breast Cancer Cells Through FOXM1,” Breast Cancer (Tokyo, Japan) 29, no. 6 (2022): 1106–1120. 36006564 10.1007/s12282-022-01391-9

[426] C. Mahe , M. Bernhard , I. Bobirnac , et al., “Functional Expression of the Serotonin 5‐HT7 Receptor in human Glioblastoma Cell Lines,” British Journal of Pharmacology 143, no. 3 (2004): 404–410. 15339860 10.1038/sj.bjp.0705936 PMC1575348

[427] V. P. Pai , A. M. Marshall , L. L. Hernandez , A. R. Buckley , and N D. Horseman , “Altered Serotonin Physiology in human Breast Cancers Favors Paradoxical Growth and Cell Survival,” Breast Cancer Research 11, no. 6 (2009): R81. 19903352 10.1186/bcr2448 PMC2815543

[428] A. Al Saedi , S. Sharma , and E. Bani Hassan , “Characterization of Skeletal Phenotype and Associated Mechanisms with Chronic Intestinal Inflammation in the Winnie Mouse Model of Spontaneous Chronic Colitis,” Inflammatory Bowel Diseases 28, no. 2 (2022): 259–272. 34347076 10.1093/ibd/izab174

[429] M. S. Shajib , U. Chauhan , S. Adeeb , et al., “Characterization of Serotonin Signaling Components in Patients With Inflammatory Bowel Disease,” Journal of the Canadian Association of Gastroenterology 2, no. 3 (2019): 132–140. 31294376 10.1093/jcag/gwy039 PMC6619411

[430] J. W. Jørandli , S. Thorsvik , H. K. Skovdahl , et al., “The Serotonin Reuptake Transporter Is Reduced in the Epithelium of Active Crohn's Disease and Ulcerative Colitis,” American journal of physiology Gastrointestinal and liver physiology 319, no. 6 (2020): G761–G768. 32967429 10.1152/ajpgi.00244.2020

[431] F. Perez , N. Kotecha , B. Lavoie , G. M. Mawe , and B A. Patel , “Monitoring Gut Epithelium Serotonin and Melatonin Overflow Provides Spatial Mapping of Inflammation,” Chembiochem 24, no. 2 (2023): e202200334. 36394122 10.1002/cbic.202200334 PMC9909162

[432] Y. H. Kwon , H. Wang , E. Denou , et al., “Modulation of Gut Microbiota Composition by Serotonin Signaling Influences Intestinal Immune Response and Susceptibility to Colitis,” Cellular and Molecular Gastroenterology and Hepatology 7, no. 4 (2019): 709–728. 30716420 10.1016/j.jcmgh.2019.01.004 PMC6462823

[433] A. Hizay , K. Dag , N. Oz , et al., “Lactobacillus Acidophilus Regulates Abnormal Serotonin Availability in Experimental Ulcerative Colitis,” Anaerobe 80 (2023): 102710. 36708801 10.1016/j.anaerobe.2023.102710

[434] S. González Delgado , I. Garza‐Veloz , F. Trejo‐Vazquez , and M. L. Martinez‐Fierro , “Interplay Between Serotonin, Immune Response, and Intestinal Dysbiosis in Inflammatory Bowel Disease,” International Journal of Molecular Sciences 23, no. 24 (2022): 15632. 36555276 10.3390/ijms232415632 PMC9779345

[435] T. Tomita , H. Fukui , D. Morishita , et al., “Efficacy of Serotonin Type 3 Receptor Antagonist Ramosetron on Diarrhea‐Predominant Irritable Bowel Syndrome (IBS‐D)‐Like Symptoms in Patients With Quiescent Inflammatory Bowel Disease: A Randomized, Double‐Blind, Placebo‐Controlled Trial,” Journal of Clinical Medicine 11, no. 23 (2022): 6882. 36498457 10.3390/jcm11236882 PMC9736938

[436] Y. H. Kwon , B. E. Blass , H. Wang , et al., “Novel 5‐HT(7) Receptor Antagonists Modulate Intestinal Immune Responses and Reduce Severity of Colitis,” American journal of physiology Gastrointestinal and liver physiology 327, no. 1 (2024): G57–G69. 38713616 10.1152/ajpgi.00299.2023 PMC11550998

[437] M. Barth , V. Serre , L. Hubert , et al., “Kinetic Analyses Guide the Therapeutic Decision in a Novel Form of Moderate Aromatic Acid Decarboxylase Deficiency,” JIMD reports 3 (2012): 25–32. 23430870 10.1007/8904_2011_43 PMC3520504

[438] L. Yang , H. Cai , J. Tou , et al., “The Role of the 5‐hydroxytryptamine Pathway in Reflux‐induced Esophageal Mucosal Injury in Rats,” World Journal of Surgical Oncology 10 (2012): 219. 23092450 10.1186/1477-7819-10-219 PMC3534590

[439] Y. Saegusa , H. Takeda , S. Muto , et al., “Decreased Motility of the Lower Esophageal Sphincter in a Rat Model of Gastroesophageal Reflux Disease May be Mediated by Reductions of Serotonin and Acetylcholine Signaling,” Biological & pharmaceutical bulletin 34, no. 5 (2011): 704–711. 21532161 10.1248/bpb.34.704

[440] A. C. Ford , A. D. Sperber , M. Corsetti , and M. Camilleri , “Irritable Bowel Syndrome,” Lancet 396, no. 10263 (2020): 1675–1688. 33049223 10.1016/S0140-6736(20)31548-8

[441] K. G. Margolis , J. F. Cryan , and E A. Mayer , “The Microbiota‐Gut‐Brain Axis: From Motility to Mood,” Gastroenterology 160, no. 5 (2021): 1486–1501. 33493503 10.1053/j.gastro.2020.10.066 PMC8634751

[442] A. Sikander , S. V. Rana , S. K. Sinha , K. K. Prasad , and S K. Arora , “Association of Serotonin Transporter Promoter Polymorphism (5‐HTTLPR) With Orocecal Transit Time in Irritable Bowel Syndrome,” Indian Journal of Gastroenterology 41, no. 6 (2022): 610–617. 36573962 10.1007/s12664-022-01280-1

[443] M. El‐Salhy , I. Wendelbo , and D. Gundersen , “Serotonin and Serotonin Transporter in the Rectum of Patients With Irritable Bowel Disease,” Molecular Medicine Reports 8, no. 2 (2013): 451–455. 23778763 10.3892/mmr.2013.1525

[444] C. Cremon , G. Carini , B. Wang , et al., “Intestinal Serotonin Release, Sensory Neuron Activation, and Abdominal Pain in Irritable bowel Syndrome,” American Journal of Gastroenterology 106, no. 7 (2011): 1290–1298. 21427712 10.1038/ajg.2011.86

[445] J. Gao , T. Xiong , G. Grabauskas , and C. Owyang , “Mucosal Serotonin Reuptake Transporter Expression in Irritable Bowel Syndrome Is Modulated by Gut Microbiota via Mast Cell‐Prostaglandin E2,” Gastroenterology 162, no. 7 (2022): 1962–1974. 35167867 10.1053/j.gastro.2022.02.016 PMC9117493

[446] Y. Mishima and S. Ishihara , “Enteric Microbiota‐Mediated Serotonergic Signaling in Pathogenesis of Irritable Bowel Syndrome,” International Journal of Molecular Sciences 22, no. 19 (2021): 10235. 34638577 10.3390/ijms221910235 PMC8508930

[447] L. Zhai , C. Huang , Z. Ning , et al., “Ruminococcus Gnavus Plays a Pathogenic Role in Diarrhea‐predominant Irritable Bowel Syndrome by Increasing Serotonin Biosynthesis,” Cell Host & Microbe 31, no. 1 (2023): 33–44. e5. 36495868 10.1016/j.chom.2022.11.006

[448] Y. Gu , C. Wang , X. Qin , et al., “Saccharomyces Boulardii, a Yeast Probiotic, Inhibits Gut Motility Through Upregulating Intestinal Serotonin Transporter and Modulating Gut Microbiota,” Pharmacological Research 181 (2022): 106291. 35690329 10.1016/j.phrs.2022.106291

[449] X. Jin , Y. Hu , T. Lin , et al., “Selenium‐enriched Bifidobacterium Longum DD98 Relieves Irritable Bowel Syndrome Induced by Chronic Unpredictable Mild Stress in Mice,” Food & Function 14, no. 11 (2023): 5355–5374. 37212199 10.1039/d2fo03408e

[450] C. Chojnacki , A. Błońska , P. Konrad , M. Chojnacki , M. Podogrocki , and T. Poplawski , “Changes in Tryptophan Metabolism on Serotonin and Kynurenine Pathways in Patients With Irritable Bowel Syndrome,” Nutrients 15, no. 5 (2023): 1262. 36904262 10.3390/nu15051262 PMC10005076

[451] Z. F. Zhang , Z. J. Duan , L. X. Wang , D. Yang , G. Zhao , and L. Zhang , “The Serotonin Transporter Gene Polymorphism (5‐HTTLPR) and Irritable Bowel Syndrome: A Meta‐analysis of 25 Studies,” BMC Gastroenterology [Electronic Resource] 14 (2014): 23. 24512255 10.1186/1471-230X-14-23 PMC3926682

[452] C. Almansa , A. Agrawal , and L A. Houghton , “Intestinal Microbiota, Pathophysiology and Translation to Probiotic Use in Patients With Irritable Bowel Syndrome,” Expert Review of Gastroenterology & Hepatology 6, no. 3 (2012): 383–398. 22646259 10.1586/egh.12.9

[453] M. Y. Areeshi , S. Haque , A. K. Panda , and R K. Mandal , “A Serotonin Transporter Gene (SLC6A4) Polymorphism Is Associated With Reduced Risk of Irritable Bowel Syndrome in American and Asian Population: A Meta‐analysis,” PLoS ONE 8, no. 9 (2013): e75567. 24069428 10.1371/journal.pone.0075567 PMC3777956

[454] L. Bosman , L. Wauters , and T. Vanuytsel , “Neuromodulating Agents in Functional Dyspepsia: A Comprehensive Review,” Acta Gastro‐Enterologica Belgica 86, no. 1 (2023): 49–57. 36842175 10.51821/86.1.10998

[455] S. Shanmugham , M. Zuber , J. E. Chan , et al., “Efficacy of Antidepressants in Functional Dyspepsia: Systematic Review and Meta‐analysis With Trial Sequential Analysis of Randomized Controlled Trials,” Indian Journal of Gastroenterology 44, no. 1 (2025): 24–34. 39180628 10.1007/s12664-024-01648-5

[456] H. Zhou , X. Tang , D. Wang , et al., “Neuroregulatory and Clinical Efficacy of Auricular Vagus Nerve Stimulation in Elderly Patients With Chronic Insomnia Comorbid With Functional Dyspepsia: Protocol for a Randomized Controlled Trial,” Frontiers in Medicine 12 (2025): 1537515. 40171499 10.3389/fmed.2025.1537515 PMC11959086

[457] A. Kourikou , G. P. Karamanolis , G. D. Dimitriadis , and K. Triantafyllou , “Gene Polymorphisms Associated With Functional Dyspepsia,” World Journal of Gastroenterology 21, no. 25 (2015): 7672–7682. 26167069 10.3748/wjg.v21.i25.7672 PMC4491956

[458] I. V. Korendovych , A. S. Svintsits'kyĭ , K. M. Revenok , and S. O. Maliarov , “[Psychopharmacological approach With the usage of selective serotonin reuptake inhibitors in functional dyspepsia treatment],” Likarska Sprava no. 11 (2014): 58–64. 25528834

[459] Y. A. Saito , G. R. Locke , A. E. Almazar , et al., “Polymorphisms of 5‐HTT LPR and GNβ3 825C>T and Response to Antidepressant Treatment in Functional Dyspepsia: A Study From the Functional Dyspepsia Treatment Trial,” American Journal of Gastroenterology 112, no. 6 (2017): 903–909. 28291238 10.1038/ajg.2017.52

[460] R. Bahuva , J. Yee , S. Gupta , and A. Atreja , “SSRI and the Risk of Gastrointestinal Bleed: More Than What Meets the Eye,” American Journal of Gastroenterology 110, no. 2 (2015): 346. 10.1038/ajg.2014.373 25646912

[461] F. C. Jing , J. Zhang , C. Feng , et al., “Potential Rat Model of Anxiety‐Like Gastric Hypersensitivity Induced by Sequential Stress,” World Journal of Gastroenterology 23, no. 42 (2017): 7594–7608. 29204059 10.3748/wjg.v23.i42.7594 PMC5698252

[462] N J. Talley , “Functional Dyspepsia: New Insights Into Pathogenesis and Therapy,” Korean Journal of Internal Medicine 31, no. 3 (2016): 444–456. 27048251 10.3904/kjim.2016.091 PMC4855108

[463] A. B. Witte , M. M. Walker , N. J. Talley , et al., “Decreased Number of Duodenal Endocrine Cells With Unaltered Serotonin‐Containing Cells in Functional Dyspepsia,” American Journal of Gastroenterology 111, no. 12 (2016): 1852–1853. 27924095 10.1038/ajg.2016.468

[464] Z. Wang , L. Wu , P. Dong , et al., “Meta‐Analysis of the Association between 5‐Hydroxytryptamine Transporter Gene‐Linked Polymorphic Region and Functional Dyspepsia and Its Subtypes,” Genet Test Mol Biomarkers 27, no. 3 (2023): 100–108. 36989523 10.1089/gtmb.2022.0151

[465] M. Jin , Y. Mo , K. Ye , M. Chen , Y. Liu , and C. He , “Efficacy of Serotonin Receptor Agonists in the Treatment of Functional Dyspepsia: A Meta‐analysis,” Archives of Medical Science 15, no. 1 (2019): 23–32. 30697251 10.5114/aoms.2017.69234 PMC6348349

[466] Z. Xiao , J. Xu , J. Tan , et al., “Zhizhu Kuanzhong, a Traditional Chinese Medicine, Alleviates Gastric Hypersensitivity and Motor Dysfunction on a Rat Model of Functional Dyspepsia,” Frontiers in pharmacology 13 (2022): 1026660. 36467071 10.3389/fphar.2022.1026660 PMC9712737

[467] J. Zhao , L. Zhao , S. Zhang , and C. Zhu , “Modified Liu‐Jun‐Zi Decoction Alleviates Visceral Hypersensitivity in Functional Dyspepsia by Regulating EC Cell‐5HT3r Signaling in Duodenum,” Journal of Ethnopharmacology 250 (2020): 112468. 31836517 10.1016/j.jep.2019.112468

[468] T. Amano , H. Ariga , A. Kurematsu , et al., “Effect of 5‐hydroxytryptamine Receptor 4 Agonist Mosapride on human Gastric Accommodation,” Neurogastroenterology and Motility 27, no. 9 (2015): 1303–1309. 26303048 10.1111/nmo.12623

[469] W. K. Goodman , E. A. Storch , and S A. Sheth , “Harmonizing the Neurobiology and Treatment of Obsessive‐Compulsive Disorder,” American Journal of Psychiatry 178, no. 1 (2021): 17–29. 33384007 10.1176/appi.ajp.2020.20111601 PMC8091795

[470] C. Xing , H. Chen , W. Bi , T. Lei , Z. Hang , and H. Du , “Targeting 5‐HT Is a Potential Therapeutic Strategy for Neurodegenerative Diseases,” International Journal of Molecular Sciences 25, no. 24 (2024): 13446. 39769209 10.3390/ijms252413446 PMC11679250

[471] D. O. Borroto‐Escuela , P. Ambrogini , B. Chruścicka , et al., “The Role of Central Serotonin Neurons and 5‐HT Heteroreceptor Complexes in the Pathophysiology of Depression: A Historical Perspective and Future Prospects,” International Journal of Molecular Sciences 22, no. 4 (2021): 1927. 33672070 10.3390/ijms22041927 PMC7919680

[472] S. Jauhar , P. J. Cowen , and M. Browning , “Fifty Years On: Serotonin and Depression,” Journal of Psychopharmacology 37, no. 3 (2023): 237–241. 36938996 10.1177/02698811231161813 PMC10076339

[473] S. H. Lin , L. T. Lee , and Y K. Yang , “Serotonin and Mental Disorders: A Concise Review on Molecular Neuroimaging Evidence,” Clinical Psychopharmacology and Neuroscience 12, no. 3 (2014): 196–202. 25598822 10.9758/cpn.2014.12.3.196 PMC4293164

[474] T. Sharp , L. Boothman , J. Raley , and P. Quérée , “Important Messages in the 'post': Recent Discoveries in 5‐HT Neurone Feedback Control,” Trends in Pharmacological Sciences 28, no. 12 (2007): 629–636. 17996955 10.1016/j.tips.2007.10.009

[475] J. P. Jacobsen , I. O. Medvedev , and M G. Caron , “The 5‐HT Deficiency Theory of Depression: Perspectives From a Naturalistic 5‐HT Deficiency Model, the Tryptophan Hydroxylase 2Arg439His Knockin Mouse,” Philosophical Transactions of the Royal Society of London. Series B: Biological Sciences 367, no. 1601 (2012): 2444–2459. 22826344 10.1098/rstb.2012.0109 PMC3405680

[476] B. A. Samuels , C. Anacker , A. Hu , et al., “5‐HT1A receptors on Mature Dentate Gyrus Granule Cells Are Critical for the Antidepressant Response,” Nature Neuroscience 18, no. 11 (2015): 1606–1616. 26389840 10.1038/nn.4116 PMC4624493

[477] B. M. Ruf and Z. Bhagwagar , “The 5‐HT1B Receptor: A Novel Target for the Pathophysiology of Depression,” Current Drug Targets 10, no. 11 (2009): 1118–1138. 19702551 10.2174/138945009789735192

[478] A. L. W. Smith , C. J. Harmer , P. J. Cowen , and S E. Murphy , “The Serotonin 1A (5‐HT(1A)) Receptor as a Pharmacological Target in Depression,” CNS Drugs 37, no. 7 (2023): 571–585. 37386328 10.1007/s40263-023-01014-7

[479] M. Głuch‐Lutwin , K. Sałaciak , K. Pytka , et al., “The 5‐HT(1A) Receptor Biased Agonist, NLX‐204, Shows Rapid‐acting Antidepressant‐Like Properties and Neurochemical Changes in Two Mouse Models of Depression,” Behavioural Brain Research 438 (2023): 114207. 36368443 10.1016/j.bbr.2022.114207

[480] M. Sekssaoui , J. Bockaert , P. Marin , and C. Bécamel , “Antidepressant‐Like Effects of Psychedelics in a Chronic Despair Mouse Model: Is the 5‐HT(2A) Receptor the Unique Player?” Neuropsychopharmacology 49, no. 4 (2024): 747–756. 38212441 10.1038/s41386-024-01794-6 PMC10876623

[481] J. Jastrzębska , M. Frankowska , I. Smaga , et al., “Evaluation of the 5‐HT(2C) Receptor Drugs RO 60‐0175, WAY 161503 and Mirtazepine in a Preclinical Model of Comorbidity of Depression and Cocaine Addiction,” Pharmacology Reports 75, no. 1 (2023): 99–118. 10.1007/s43440-022-00428-2 PMC9889480 36374478

[482] S. Bhatt , T. Devadoss , S. N. Manjula , and J. Rajangam , “HT(3) Receptor Antagonism a Potential Therapeutic Approach for the Treatment of Depression and Other Disorders,” Current Neuropharmacology 19, no. 9 (2021): 1545–1559. 33059577 10.2174/1570159X18666201015155816 PMC8762176

[483] R. Hamati , M. El Mansari , and P. Blier , “Serotonin‐2B Receptor Antagonism Increases the Activity of Dopamine and Glutamate Neurons in the Presence of Selective Serotonin Reuptake Inhibition,” Neuropsychopharmacology 45, no. 12 (2020): 2098–2105. 32473594 10.1038/s41386-020-0723-y PMC7547697

[484] A. N. de Cates , C. J. Harmer , P. J. Harrison , et al., “Association Between a Selective 5‐HT(4) Receptor Agonist and Incidence of Major Depressive Disorder: Emulated Target Trial,” British Journal of Psychiatry 225, no. 3 (2024): 371–378. 10.1192/bjp.2024.97 PMC7616487 39109752

[485] Y. Kawahara , H. Kawahara , F. Kaneko , and M. Tanaka , “Long‐term Administration of citalopram Reduces Basal and Stress‐induced Extracellular Noradrenaline Levels in Rat Brain,” Psychopharmacology 194, no. 1 (2007): 73–81. 17534604 10.1007/s00213-007-0826-8

[486] G. S. Sachs , P. P. Yeung , L. Rekeda , A. Khan , J. L. Adams , and M. Fava , “Adjunctive Cariprazine for the Treatment of Patients with Major Depressive Disorder: A Randomized, Double‐Blind, Placebo‐Controlled Phase 3 Study,” American Journal of Psychiatry 180, no. 3 (2023): 241–251. 36789515 10.1176/appi.ajp.20220504

[487] D. Gupta , V. Prabhakar , and M. Radhakrishnan , “5HT3 receptors: Target for New Antidepressant Drugs,” Neuroscience and Biobehavioral Reviews 64 (2016): 311–325. 26976353 10.1016/j.neubiorev.2016.03.001

[488] O. Mnie‐Filali , C. Faure , L. Lambás‐Señas , et al., “Pharmacological Blockade of 5‐HT7 Receptors as a Putative Fast Acting Antidepressant Strategy,” Neuropsychopharmacology 36, no. 6 (2011): 1275–1288. 21326194 10.1038/npp.2011.13 PMC3079839

[489] S. Bhatt , J. Kanoujia , S. Mohana Lakshmi , et al., “Role of Brain‐Gut‐Microbiota Axis in Depression: Emerging Therapeutic Avenues,” CNS & Neurological Disorders ‐ Drug Targets 22, no. 2 (2023): 276–288. 35352640 10.2174/1871527321666220329140804

[490] J. Ma , R. Wang , Y. Chen , Z. Wang , and Y. Dong , “5‐HT Attenuates Chronic Stress‐induced Cognitive Impairment in Mice Through Intestinal Flora Disruption,” Journal of Neuroinflammation 20, no. 1 (2023): 23. 36737776 10.1186/s12974-023-02693-1 PMC9896737

[491] Y. P. López‐Echeverri , K. J. Cardona‐Londoño , J. F. Garcia‐Aguirre , and M. Orrego‐Cardozo , “Effects of Serotonin Transporter and Receptor Polymorphisms on Depression,” Revista Colombiana de Psiquiatría (English Edition) 52, no. 2 (2023): 130–138. 10.1016/j.rcpeng.2021.07.003 37453823

[492] D. Naber and M. Bullinger , “Should Antidepressants be Used in minor Depression?” Dialogues in Clinical Neuroscience 20, no. 3 (2018): 223–228. 30581292 10.31887/DCNS.2018.20.3/dnaber PMC6296391

[493] W. Wei , L. Deng , C. Qiao , et al., “Neural Variability in Three Major Psychiatric Disorders,” Molecular Psychiatry 28, no. 12 (2023): 5217–5227. 37443193 10.1038/s41380-023-02164-2

[494] C. A. Tamminga and H H. Holcomb , “Phenotype of Schizophrenia: A Review and Formulation,” Molecular Psychiatry 10, no. 1 (2005): 27–39. 15340352 10.1038/sj.mp.4001563

[495] K. Hrovatin , T. Kunej , and V. Dolžan , “Genetic Variability of Serotonin Pathway Associated With Schizophrenia Onset, Progression, and Treatment,” American Journal of Medical Genetics. Part B, Neuropsychiatric Genetics 183, no. 2 (2020): 113–127. 10.1002/ajmg.b.32766 31674148

[496] A. E. Eggers , “A Serotonin Hypothesis of Schizophrenia,” Medical Hypotheses 80, no. 6 (2013): 791–794. 23557849 10.1016/j.mehy.2013.03.013

[497] R. Yamada , A. Wada , A. Stickley , Y. Yokoi , and T. Sumiyoshi , “Effect of 5‐HT1A Receptor Partial Agonists of the Azapirone Class as an Add‐On Therapy on Psychopathology and Cognition in Schizophrenia: A Systematic Review and Meta‐Analysis,” The International Journal of Neuropsychopharmacology 26, no. 4 (2023): 249–258. 36721972 10.1093/ijnp/pyad004 PMC10109009

[498] R. Yamada , A. Wada , A. Stickley , A. Newman‐Tancredi , and T. Sumiyoshi , “Augmentation Therapy with Serotonin 5‐HT(1A) Receptor Partial Agonists on Cognitive Function in Depressive Disorders: A Systematic Review of Randomized Controlled Studies,” Neuropsychopharmacology Reports 45, no. 2 (2025): e70023. 40421605 10.1002/npr2.70023 PMC12107367

[499] S. Selvaraj , D. Arnone , A. Cappai , and O. Howes , “Alterations in the Serotonin System in Schizophrenia: A Systematic Review and Meta‐analysis of Postmortem and Molecular Imaging Studies,” Neuroscience and Biobehavioral Reviews 45 (2014): 233–245. 24971825 10.1016/j.neubiorev.2014.06.005

[500] M. A. Cummings , A. W. Arias , and S M. Stahl , “What Is the Neurobiology of Schizophrenia?” CNS Spectrums 30, no. 1 (2024): e13. 39473188 10.1017/S1092852924000518 PMC13064734

[501] K. Thomas and A. Saadabadi , “Olanzapine,” StatPearls (StatPearls Publishing Copyright © 2025, StatPearls Publishing LLC., 2025).

[502] E. Kossatz , R. Diez‐Alarcia , S. A. Gaitonde , et al., “G Protein‐specific Mechanisms in the Serotonin 5‐HT(2A) Receptor Regulate Psychosis‐related Effects and Memory Deficits,” Nature Communications 15, no. 1 (2024): 4307. 10.1038/s41467-024-48196-2 PMC11137019 38811567

[503] M. Tarzian , M. Soudan , M. Alhajji , M. Ndrio , and A O. Fakoya , “Lurasidone for Treating Schizophrenia and Bipolar Depression: A Review of Its Efficacy,” Cureus 15, no. 4 (2023): e38071. 37228542 10.7759/cureus.38071 PMC10208134

[504] L. A. Márquez , A. Meneses , and E J. Galván , “5‐HT(6) Receptors Control GABAergic Transmission and CA1 Pyramidal Cell Output of Dorsal Hippocampus,” Neuroscience 532 (2023): 65–78. 37776946 10.1016/j.neuroscience.2023.09.013

[505] I. Muneta‐Arrate , P. Miranda‐Azpiazu , I. Horrillo , R. Diez‐Alarcia , and J J. Meana , “Ligand Bias and Inverse Agonism on 5‐HT(2A) Receptor‐mediated Modulation of G Protein Activity in Post‐mortem human Brain,” British Journal of Pharmacology 182, no. 14 (2025): 3320–3335. 38644550 10.1111/bph.16368

[506] Y. S. Park , G. M. Kim , H. J. Sung , J. Y. Yu , and K W. Sung , “Atypical Antipsychotic Drug Olanzapine Inhibits 5‐HT(3) Receptor‐mediated Currents by Allosteric and Non‐competitive Mechanisms,” Korean Journal of Physiology and Pharmacology 29, no. 4 (2025): 431–442. 39690469 10.4196/kjpp.24.340 PMC12198442

[507] O. Kassar , O. Shaheen , A. Selim , et al., “Efficacy and Safety of ondansetron in Schizophrenia: A Systematic Review and Meta‐analysis of Randomized Controlled Trials,” General Hospital Psychiatry 96 (2025): 37–46. 40482395 10.1016/j.genhosppsych.2025.06.001

[508] A. Nikiforuk , M. Hołuj , T. Kos , and P. Popik , “The Effects of a 5‐HT5A Receptor Antagonist in a Ketamine‐based Rat Model of Cognitive Dysfunction and the Negative Symptoms of Schizophrenia,” Neuropharmacology 105 (2016): 351–360. 26826431 10.1016/j.neuropharm.2016.01.035

[509] R. Okubo , T. Hasegawa , K. Fukuyama , T. Shiroyama , and M. Okada , “Current Limitations and Candidate Potential of 5‐HT7 Receptor Antagonism in Psychiatric Pharmacotherapy,” Front Psychiatry 12 (2021): 623684. 33679481 10.3389/fpsyt.2021.623684 PMC7930824

[510] R. Galici , J. D. Boggs , K. L. Miller , P. Bonaventure , and J R. Atack , “Effects of SB‐269970, a 5‐HT7 Receptor Antagonist, in Mouse Models Predictive of Antipsychotic‐Like Activity,” Behavioural Pharmacology 19, no. 2 (2008): 153–159. 18332680 10.1097/FBP.0b013e3282f62d8c

[511] W. G. Frankle , I. Lombardo , L. S. Kegeles , et al., “Serotonin 1A Receptor Availability in Patients With Schizophrenia and Schizo‐affective Disorder: A Positron Emission Tomography Imaging Study With [11C]WAY 100635,” Psychopharmacology 189, no. 2 (2006): 155–164. 16953380 10.1007/s00213-006-0543-8

[512] S. Alvarez‐Herrera , M. Rosel Vales , G. Pérez‐Sánchez , et al., “Risperidone Decreases Expression of Serotonin Receptor‐2A (5‐HT2A) and Serotonin Transporter (SERT) but Not Dopamine Receptors and Dopamine Transporter (DAT) in PBMCs From Patients With Schizophrenia,” Pharmaceuticals (Basel) 17, no. 2 (2024): 167. 38399382 10.3390/ph17020167 PMC10892557

[513] H. Y. Li , S. Y. Huang , D. D. Zhou , et al., “Theabrownin Inhibits Obesity and Non‐alcoholic Fatty Liver Disease in Mice via Serotonin‐related Signaling Pathways and Gut‐liver Axis,” Journal of Advanced Research 52 (2023): 59–72. 36639024 10.1016/j.jare.2023.01.008 PMC10555776

[514] M. Kimura , H. Moteki , and M. Ogihara , “Role of Hepatocyte Growth Regulators in Liver Regeneration,” Cells 12, no. 2 (2023): 208. 36672143 10.3390/cells12020208 PMC9856461

[515] M. Lesurtel and P A. Clavien , “Platelet‐derived Serotonin: Translational Implications for Liver Regeneration,” Hepatology 60, no. 1 (2014): 30–33. 24700245 10.1002/hep.27067

[516] P. Starlinger , S. Haegele , F. Offensperger , et al., “The Profile of Platelet α‐granule Released Molecules Affects Postoperative Liver Regeneration,” Hepatology 63, no. 5 (2016): 1675–1688. 26528955 10.1002/hep.28331

[517] T. Yoshizumi , S. Itoh , D. Imai , et al., “Impact of Platelets and Serotonin on Liver Regeneration After Living Donor Hepatectomy,” Transplantation Proceedings 47, no. 3 (2015): 683–685. 25891711 10.1016/j.transproceed.2014.11.050

[518] C. He , M. Zhai , B. Shu , C. Deng , L. Li , and S. Liu , “5‐HT and Intraplatelet 5‐HT: A Potential Upstream Regulator of YAP in Liver Regeneration,” Experimental & Molecular Medicine 51, no. 10 (2019): 1–2. 10.1038/s12276-019-0324-1 PMC6802664 31619665

[519] Y. Fang , C. Liu , B. Shu , et al., “Axis of Serotonin ‐pERK‐YAP in Liver Regeneration,” Life Sciences 209 (2018): 490–497. 30142376 10.1016/j.lfs.2018.08.047

[520] M. R. Ebrahimkhani , F. Oakley , L. B. Murphy , et al., “Stimulating Healthy Tissue Regeneration by Targeting the 5‐HT_2_B Receptor in Chronic Liver Disease,” Nature Medicine 17, no. 12 (2011): 1668–1673. 10.1038/nm.2490 PMC3428919 22120177

[521] Y. Wen , C. Emontzpohl , L. Xu , et al., “Interleukin‐33 Facilitates Liver Regeneration Through Serotonin‐involved Gut‐liver Axis,” Hepatology 77, no. 5 (2023): 1580–1592. 36129070 10.1002/hep.32744 PMC10758291

[522] S. Pyroja , B. Joseph , and C S. Paulose , “Increased 5‐HT2C Receptor Binding in the Brain Stem and Cerebral Cortex During Liver Regeneration and Hepatic Neoplasia in Rats,” Journal of the Neurological Sciences 254, no. 1‐2 (2007): 3–8. 17258772 10.1016/j.jns.2006.12.003

[523] S. Redenšek Trampuž , S. van Riet , Å. Nordling , and M. Ingelman‐Sundberg , “Mechanisms of 5‐HT Receptor Antagonists in the Regulation of Fibrosis in a 3D human Liver Spheroid Model,” Scientific Reports 14, no. 1 (2024): 1396. 38228622 10.1038/s41598-023-49240-9 PMC10792007

[524] J. Fu , C. Li , G. Zhang , et al., “Crucial Roles of 5‐HT and 5‐HT2 Receptor in Diabetes‐Related Lipid Accumulation and Pro‐Inflammatory Cytokine Generation in Hepatocytes,” Cellular Physiology and Biochemistry 48, no. 6 (2018): 2409–2428. 30121645 10.1159/000492656

[525] C. Chung and Y. Iwakiri , “Activated Hepatic Stellate Cells: Negative Regulators of Hepatocyte Proliferation in Liver Diseases,” Hepatology 56, no. 1 (2012): 389–391. 22876366 10.1002/hep.25761 PMC3666938

[526] J. Yoon , W. I. Choi , W. H. Lee , et al., “Synthesis and Biological Evaluation of Peripheral 5HT(2B) Antagonists for Liver Fibrosis,” Journal of Medicinal Chemistry 68, no. 6 (2025): 6493–6506. 40048549 10.1021/acs.jmedchem.4c03003

[527] K. Kyritsi , L. Chen , and A. O'Brien , “Modulation of the Tryptophan Hydroxylase 1/Monoamine Oxidase‐A/5‐Hydroxytryptamine/5‐Hydroxytryptamine Receptor 2A/2B/2C Axis Regulates Biliary Proliferation and Liver Fibrosis during Cholestasis,” Hepatology 71, no. 3 (2020): 990–1008. 31344280 10.1002/hep.30880 PMC6993623

[528] R. G. Ruddell , F. Oakley , Z. Hussain , et al., “A Role for Serotonin (5‐HT) in Hepatic Stellate Cell Function and Liver Fibrosis,” American Journal of Pathology 169, no. 3 (2006): 861–876. 16936262 10.2353/ajpath.2006.050767 PMC1698820

[529] B. Polat , Z. Halici , E. Cadirci , et al., “Liver 5‐HT7 Receptors: A Novel Regulator Target of Fibrosis and Inflammation‐induced Chronic Liver Injury in Vivo and in Vitro,” International Immunopharmacology 43 (2017): 227–235. 28043031 10.1016/j.intimp.2016.12.023

[530] J. Park , W. Jeong , C. Yun , H. Kim , and C M. Oh , “Serotonergic Regulation of Hepatic Energy Metabolism,” Endocrinology and Metabolism (Seoul) 36, no. 6 (2021): 1151–1160. 10.3803/EnM.2021.1331 PMC8743581 34911172

[531] C. Teunis , M. Nieuwdorp , and N. Hanssen , “Interactions Between Tryptophan Metabolism, the Gut Microbiome and the Immune System as Potential Drivers of Non‐Alcoholic Fatty Liver Disease (NAFLD) and Metabolic Diseases,” Metabolites 12, no. 6 (2022): 514. 35736447 10.3390/metabo12060514 PMC9227929

[532] A. Nocito , F. Dahm , W. Jochum , et al., “Serotonin Mediates Oxidative Stress and Mitochondrial Toxicity in a Murine Model of Nonalcoholic Steatohepatitis,” Gastroenterology 133, no. 2 (2007): 608–618. 17681180 10.1053/j.gastro.2007.05.019

[533] J. D. Crane , R. Palanivel , E. P. Mottillo , et al., “Inhibiting Peripheral Serotonin Synthesis Reduces Obesity and Metabolic Dysfunction by Promoting Brown Adipose Tissue Thermogenesis,” Nature Medicine 21, no. 2 (2015): 166–172. 10.1038/nm.3766 PMC5647161 25485911

[534] J. Binetti , L. Bertran , D. Riesco , et al., “Deregulated Serotonin Pathway in Women With Morbid Obesity and NAFLD,” Life (Basel) 10, no. 10 (2020): 245. 33081272 10.3390/life10100245 PMC7603041

[535] L. Wang , X. Fan , and J. Han , “Gut‐Derived Serotonin Contributes to the Progression of Non‐Alcoholic Steatohepatitis via the Liver HTR2A/PPARγ2 Pathway,” Frontiers in pharmacology 11 (2020): 553. 32477107 10.3389/fphar.2020.00553 PMC7240039

[536] W. Choi , J. Namkung , I. Hwang , et al., “Serotonin Signals Through a Gut‐liver Axis to Regulate Hepatic Steatosis,” Nature Communications 9, no. 1 (2018): 4824. 10.1038/s41467-018-07287-7 PMC6240035 30446669

[537] L. Sessa , S. Concilio , J. Fominaya , D. Eletto , S. Piotto , and X. Busquets , “A New Serotonin 2A Receptor Antagonist With Potential Benefits in Non‐Alcoholic Fatty Liver Disease,” Life Sciences 314 (2023): 121315. 36581095 10.1016/j.lfs.2022.121315

[538] M. Kim , W. Choi , J. Yoon , et al., “Synthesis and Biological Evaluation of Tyrosine Derivatives as Peripheral 5HT(2A) Receptor Antagonists for Nonalcoholic Fatty Liver Disease,” European Journal of Medicinal Chemistry 239 (2022): 114517. 35732081 10.1016/j.ejmech.2022.114517

[539] M. H. Kim , S. J. Kim , W. J. Park , D. H. Lee , and K K. Kim , “GR113808, a Serotonin Receptor 4 Antagonist, Prevents High‐fat‐diet‐induced Obesity, Fatty Liver Formation, and Insulin Resistance in C57BL/6J Mice,” BMC Pharmacology and Toxicology 25, no. 1 (2024): 76. 39394150 10.1186/s40360-024-00800-3 PMC11470721

[540] S. Haub , Y. Ritze , I. Ladel , et al., “Serotonin Receptor Type 3 Antagonists Improve Obesity‐associated Fatty Liver Disease in Mice,” Journal of Pharmacology and Experimental Therapeutics 339, no. 3 (2011): 790–798. 21903748 10.1124/jpet.111.181834

[541] D. Chen , Y. Wang , J. Yang , et al., “Shenling Baizhu San Ameliorates Non‐alcoholic Fatty Liver Disease in Mice by Modulating Gut Microbiota and Metabolites,” Frontiers in pharmacology 15 (2024): 1343755. 38720776 10.3389/fphar.2024.1343755 PMC11076757

[542] L. Cao , J. Chen , Y. Wang , et al., “Identification of Serotonin 2A Receptor as a Novel HCV Entry Factor by a Chemical Biology Strategy,” Protein Cell 10, no. 3 (2019): 178–195. 29542010 10.1007/s13238-018-0521-z PMC6338621

[543] Y M. Wang , “[Abnormal platelet function and ultrastructure in patients With severe viral hepatitis],” Zhonghua Nei Ke Za Zhi [Chinese Journal of Internal Medicine] 29, no. 7 (1990): 416–418. 445. 1704303

[544] J. M. Loftis , B. J. Morasco , D. Menasco , D. Fuchs , M. Strater , and P. Hauser , “Serum Serotonin Levels Are Associated With Antiviral Therapy Outcomes in Patients With Chronic Hepatitis C,” Open Infect Dis J 4 (2010): 132–141. 21151716 10.2174/1874279301004010132 PMC2999909

[545] P. A. Lang , C. Contaldo , P. Georgiev , et al., “Aggravation of Viral hepatitis by Platelet‐derived Serotonin,” Nature Medicine 14, no. 7 (2008): 756–761. 10.1038/nm1780 18516052

[546] C. M. Chang , M. S. Hsieh , T. C. Yang , et al., “Selective Serotonin Reuptake Inhibitors and the Risk of Hepatocellular Carcinoma in hepatitis B Virus‐infected Patients,” Cancer Management and Research 9 (2017): 709–720. 29238221 10.2147/CMAR.S148097 PMC5713708

[547] G. Sumara , O. Sumara , J. K. Kim , and G. Karsenty , “Gut‐derived Serotonin Is a Multifunctional Determinant to Fasting Adaptation,” Cell metabolism 16, no. 5 (2012): 588–600. 23085101 10.1016/j.cmet.2012.09.014 PMC3696514

[548] K. Y. Xie , S. J. Chien , B. C. Tan , and Y W. Chen , “RNA Editing of 5‐HT(2C) R Impairs Insulin Secretion of Pancreatic Beta Cells via Altered Store‐operated Calcium Entry,” Faseb Journal 35, no. 10 (2021): e21929. 34553421 10.1096/fj.202100265RR

[549] E. Takishita , A. Takahashi , N. Harada , M. Yamato , M. Yoshizumi , and Y. Nakaya , “Effect of Sarpogrelate Hydrochloride, a 5‐HT2 Blocker, on Insulin Resistance in Otsuka Long‐Evans Tokushima Fatty Rats (OLETF rats), a Type 2 Diabetic Rat Model,” Journal of Cardiovascular Pharmacology 43, no. 2 (2004): 266–270. 14716215 10.1097/00005344-200402000-00015

[550] J. Yamada , Y. Sugimoto , T. Yoshikawa , I. Kimura , and K. Horisaka , “The Involvement of the Peripheral 5‐HT2A Receptor in Peripherally Administered Serotonin‐induced Hyperglycemia in Rats,” Life Sciences 57, no. 8 (1995): 819–825. 7637555 10.1016/0024-3205(95)02010-g

[551] H. Watanabe , T. Nakano , R. Saito , et al., “Serotonin Improves High Fat Diet Induced Obesity in Mice,” PLoS ONE 11, no. 1 (2016): e0147143. 26766570 10.1371/journal.pone.0147143 PMC4713156

[552] K. V. Derkach , V. M. Bondareva , O. V. Chistyakova , L. M. Berstein , and A O. Shpakov , “The Effect of Long‐Term Intranasal Serotonin Treatment on Metabolic Parameters and Hormonal Signaling in Rats With High‐Fat Diet/Low‐Dose Streptozotocin‐Induced Type 2 Diabetes,” International Journal of Endocrinology 2015 (2015): 245459. 26124826 10.1155/2015/245459 PMC4466391

[553] T. C. Chi , Y. J. Ho , W. P. Chen , et al., “Serotonin Enhances Beta‐endorphin Secretion to Lower Plasma Glucose in Streptozotocin‐induced Diabetic Rats,” Life Sciences 80, no. 20 (2007): 1832–1838. 17397876 10.1016/j.lfs.2007.02.016

[554] H. Chen , F. Hong , Y. Chen , et al., “Activation of Islet 5‐HT4 Receptor Regulates Glycemic Control Through Promoting Insulin Secretion,” European Journal of Pharmacology 789 (2016): 354–361. 27423314 10.1016/j.ejphar.2016.07.024

[555] K. Kim , C. M. Oh , M. Ohara‐Imaizumi , et al., “Functional Role of Serotonin in Insulin Secretion in a Diet‐induced Insulin‐resistant state,” Endocrinology 156, no. 2 (2015): 444–452. 25426873 10.1210/en.2014-1687 PMC4298319

[556] N. Paulmann , M. Grohmann , J. P. Voigt , et al., “Intracellular Serotonin Modulates Insulin Secretion From Pancreatic Beta‐cells by Protein Serotonylation,” Plos Biology 7, no. 10 (2009): e1000229. 19859528 10.1371/journal.pbio.1000229 PMC2760755

[557] M. Hasni Ebou , A. Singh‐Estivalet , and J. M. Launay , “Glucocorticoids Inhibit Basal and Hormone‐Induced Serotonin Synthesis in Pancreatic Beta Cells,” PLoS ONE 11, no. 2 (2016): e0149343. 26901633 10.1371/journal.pone.0149343 PMC4763453

[558] N. Liu , T. Liu , N. Alim , et al., “5‐HT Promotes Pancreatic α‐to‐β Cell Transdifferentiation,” Biochimica et Biophysica Acta (BBA) ‐ Molecular Cell Research 1872, no. 5 (2025): 119958. 40222656 10.1016/j.bbamcr.2025.119958

[559] J. García‐Pedraza , O. Hernández‐Abreu , A. Morán , J. Carretero , M. García‐Domingo , and C M. Villalón , “Role of Peripheral 5‐HT(5A) Receptors in 5‐HT‐induced Cardiac Sympatho‐inhibition in Type 1 Diabetic Rats,” Scientific Reports 10, no. 1 (2020): 19358. 33168874 10.1038/s41598-020-76298-6 PMC7652863

[560] M. Li , X. Chen , N. Cao , R. Lv , and B. Gu , “Improvement of Urethral Dysfunction by 5‐HT(1A) Receptor Agonist NLX‐112 in Diabetic Rats,” Neurourology and Urodynamics 41, no. 7 (2022): 1528–1538. 35870169 10.1002/nau.24993

[561] B. Jin , S. E. Ha , L. Wei , et al., “Colonic Motility Is Improved by the Activation of 5‐HT(2B) Receptors on Interstitial Cells of Cajal in Diabetic Mice,” Gastroenterology 161, no. 2 (2021): 608–622. e7. 33895170 10.1053/j.gastro.2021.04.040 PMC8532042

[562] N. Munawar , M. S. Bitar , and W. Masocha , “Activation of 5‐HT1A Receptors Normalizes the Overexpression of Presynaptic 5‐HT1A Receptors and Alleviates Diabetic Neuropathic Pain,” International Journal of Molecular Sciences 24, no. 18 (2023): 14334. 37762636 10.3390/ijms241814334 PMC10532078

[563] J. Shuai , Y. Gao , L. Chen , and Z. Wang , “Role of Serotonin in Regulation of Pancreatic and Mesenteric Arterial Function in Diabetic Mice,” European Journal of Pharmacology 901 (2021): 174070. 33798598 10.1016/j.ejphar.2021.174070

[564] K. Hellstrand , C. Czerkinsky , A. Ricksten , et al., “Role of Serotonin in the Regulation of Interferon‐gamma Production by human Natural Killer Cells,” Journal of Interferon Research 13, no. 1 (1993): 33–38. 8454908 10.1089/jir.1993.13.33

[565] K. Iken , S. Chheng , A. Fargin , A. C. Goulet , and E. Kouassi , “Serotonin Upregulates Mitogen‐stimulated B Lymphocyte Proliferation Through 5‐HT1A Receptors,” Cellular Immunology 163, no. 1 (1995): 1–9. 7758118 10.1006/cimm.1995.1092

[566] M. Wan , Z. Ma , J. Han , et al., “5‐HT Induces Regulatory B Cells in Fighting Against Inflammation‐driven Ulcerative Colitis,” International Immunopharmacology 125, no. Pt A (2023): 111042. 37866311 10.1016/j.intimp.2023.111042

[567] T. Müller , T. Dürk , B. Blumenthal , et al., “5‐hydroxytryptamine Modulates Migration, Cytokine and Chemokine Release and T‐cell Priming Capacity of Dendritic Cells in Vitro and in Vivo,” PLoS ONE 4, no. 7 (2009): e6453. 19649285 10.1371/journal.pone.0006453 PMC2714071

[568] N. Katoh , F. Soga , T. Nara , et al., “Effect of Serotonin on the Differentiation of human Monocytes Into Dendritic Cells,” Clinical and Experimental Immunology 146, no. 2 (2006): 354–361. 17034589 10.1111/j.1365-2249.2006.03197.x PMC1942053

[569] L. Fabà , N. de Groot , G. Ramis , C. G. Cabrera‐Gómez , and J. Doelman , “Serotonin Receptors and Their Association With the Immune System in the Gastrointestinal Tract of Weaning Piglets,” Porcine Health Manag 8, no. 1 (2022): 8. 35090573 10.1186/s40813-022-00250-5 PMC8796611

[570] S. Wang , L. Kong , L. Wang , et al., “Viral Expression of NE/PPE Enhances Anti‐colorectal Cancer Efficacy of Oncolytic adenovirus by Promoting TAM M1 Polarization to Reverse Insufficient Effector Memory/Effector CD8(+) T Cell Infiltration,” Journal of Experimental & Clinical Cancer Research 44, no. 1 (2025): 97. 40082916 10.1186/s13046-025-03358-y PMC11907943

[571] K. Liu , L. Kong , H. Cui , et al., “Thymosin α1 Reverses Oncolytic adenovirus‐induced M2 Polarization of Macrophages to Improve Antitumor Immunity and Therapeutic Efficacy,” Cell Reports Medicine 5, no. 10 (2024): 101751. 39357524 10.1016/j.xcrm.2024.101751 PMC11513825

[572] L. Xiao , L. Zhang , C. Guo , et al., “Find Me" and "Eat Me" Signals: Tools to Drive Phagocytic Processes for Modulating Antitumor Immunity,” Cancer communications (London) 44, no. 7 (2024): 791–832. 10.1002/cac2.12579 PMC11260773 38923737

[573] J. Molina‐Cerrillo , E. Grande , and T. Alonso‐Gordoa , “Inhibition of Serotonin Synthesis May Have Antitumor Activity? Long‐Term Efficacy in a Patient With Gastrointestinal Neuroendocrine Tumor,” The Oncologist 24, no. 7 (2019): e597–e599. 30877189 10.1634/theoncologist.2018-0776 PMC6656452

[574] A. Szabo , P. Gogolak , G. Koncz , et al., “Immunomodulatory Capacity of the Serotonin Receptor 5‐HT2B in a Subset of human Dendritic Cells,” Scientific Reports 8, no. 1 (2018): 1765. 29379077 10.1038/s41598-018-20173-y PMC5788853

[575] S. Karmakar and G. Lal , “Role of Serotonergic System in Regulating Brain Tumor‐Associated Neuroinflammatory Responses,” Methods in Molecular Biology 2761 (2024): 181–207. 38427238 10.1007/978-1-0716-3662-6_14

[576] A. D. Waldman , J. M. Fritz , and M J. Lenardo , “A Guide to Cancer Immunotherapy: From T Cell Basic Science to Clinical Practice,” Nature Reviews Immunology 20, no. 11 (2020): 651–668. 10.1038/s41577-020-0306-5 PMC7238960 32433532

[577] H. Zhang , Y. Zhang , J. Dong , et al., “Recombinant Oncolytic adenovirus Expressing a Soluble PVR Elicits Long‐term Antitumor Immune Surveillance,” Molecular Therapy Oncolytics 20 (2021): 12–22. 33575467 10.1016/j.omto.2020.11.001 PMC7851489

[578] H. Zhang , Y. Zhang , J. Dong , et al., “Recombinant adenovirus Expressing the Fusion Protein PD1PVR Improves CD8(+) T Cell‐mediated Antitumor Efficacy With Long‐term Tumor‐specific Immune Surveillance in Hepatocellular Carcinoma,” Cellular oncology (Dordrecht) 44, no. 6 (2021): 1243–1255. 10.1007/s13402-021-00633-w PMC12980736 34491549

[579] Y. Zhang , J. Wu , H. Zhang , J. Wei , and J. Wu , “Extracellular Vesicles‐Mimetic Encapsulation Improves Oncolytic Viro‐Immunotherapy in Tumors with Low Coxsackie and Adenovirus Receptor,” Frontiers in Bioengineering and Biotechnology 8 (2020): 574007. 33042975 10.3389/fbioe.2020.574007 PMC7525182

[580] A. Chen , Y. Zhang , G. Meng , et al., “Oncolytic Measles Virus Enhances Antitumour Responses of Adoptive CD8(+)NKG2D(+) Cells in Hepatocellular Carcinoma Treatment,” Scientific Reports 7, no. 1 (2017): 5170. 28701757 10.1038/s41598-017-05500-z PMC5507973

[581] A. Quintero‐Villegas and S I. Valdés‐Ferrer , “Role of 5‐HT(7) Receptors in the Immune System in Health and Disease,” Molecular Medicine 26, no. 1 (2019): 2. 31892309 10.1186/s10020-019-0126-x PMC6938607

[582] D. Zhan , X. Wang , Y. Zheng , et al., “Integrative Dissection of 5‐hydroxytryptamine Receptors‐related Signature in the Prognosis and Immune Microenvironment of Breast Cancer,” Frontiers in oncology 13 (2023): 1147189. 37795441 10.3389/fonc.2023.1147189 PMC10546427

[583] X. Wang , S. Q. Fu , X. Yuan , et al., “A GAPDH Serotonylation System Couples CD8(+) T Cell Glycolytic Metabolism to Antitumor Immunity,” Molecular Cell 84, no. 4 (2024): 760–775. e7. 38215751 10.1016/j.molcel.2023.12.015

[584] S. Zhou , D. Ye , H. Xia , et al., “Sertraline Inhibits Stress‐induced Tumor Growth Through Regulating CD8 + T Cell‐mediated Anti‐tumor Immunity,” Anti‐Cancer Drugs 33, no. 9 (2022): 935–942. 36066403 10.1097/CAD.0000000000001383

[585] K. Hellstrand and S. Hermodsson , “Role of Serotonin in the Regulation of human Natural Killer Cell Cytotoxicity,” Journal of Immunology 139, no. 3 (1987): 869–875. 2439597

[586] B. Li , J. Elsten‐Brown , M. Li , et al., “Serotonin Transporter Inhibits Antitumor Immunity Through Regulating the Intratumoral Serotonin Axis,” Cell 188, no. 14 (2025): 3823–3842. 40403728 10.1016/j.cell.2025.04.032 PMC12255530

[587] N. Pivac , D. Kozaric‐Kovacic , M. Mustapic , et al., “Platelet Serotonin in Combat Related Posttraumatic Stress Disorder With Psychotic Symptoms,” Journal of Affective Disorders 93, no. 1‐3 (2006): 223–227. 16647142 10.1016/j.jad.2006.02.018

[588] B. W. Okaty , K. G. Commons , and S M. Dymecki , “Embracing Diversity in the 5‐HT Neuronal System,” Nature Reviews Neuroscience 20, no. 7 (2019): 397–424. 30948838 10.1038/s41583-019-0151-3

[589] S. Senol and M U. Es , “Is Serotonin a Valuable Parameter in Peripheral Arterial Disease?” Asian Cardiovascular & Thoracic Annals 23, no. 3 (2015): 289–291. 25114324 10.1177/0218492314546908

[590] R. L. I. Pillai , M. Zhang , J. Yang , et al., “Will Imaging Individual Raphe Nuclei in Males With Major Depressive Disorder Enhance Diagnostic Sensitivity and Specificity?” Depression and Anxiety 35, no. 5 (2018): 411–420. 29365217 10.1002/da.22721 PMC5934332

[591] S. Jauhar , D. Arnone , D. S. Baldwin , et al., “A Leaky Umbrella Has Little Value: Evidence Clearly Indicates the Serotonin System Is Implicated in Depression,” Molecular Psychiatry 28, no. 8 (2023): 3149–3152. 37322065 10.1038/s41380-023-02095-y PMC10618084

[592] A. Mohammadi , E. Rashidi , and V G. Amooeian , “Brain, Blood, Cerebrospinal Fluid, and Serum Biomarkers in Schizophrenia,” Psychiatry Research 265 (2018): 25–38. 29680514 10.1016/j.psychres.2018.04.036

[593] C. Li , Q. Cai , Z. Su , Z. Chen , J. Cao , and F. Xu , “Could Peripheral 5‐HT Level be Used as a Biomarker for Depression Diagnosis and Treatment? A Narrative Minireview,” Frontiers in pharmacology 14 (2023): 1149511. 36959863 10.3389/fphar.2023.1149511 PMC10028199

[594] A. L. Lopresti , G. L. Maker , S. D. Hood , and P D. Drummond , “A Review of Peripheral Biomarkers in Major Depression: The Potential of Inflammatory and Oxidative Stress Biomarkers,” Progress in Neuro‐Psychopharmacology & Biological Psychiatry 48 (2014): 102–111. 24104186 10.1016/j.pnpbp.2013.09.017

[595] M. Hagbom , F. M. De Faria , M. E. Winberg , et al., “Neurotrophic Factors Protect the Intestinal Barrier From Rotavirus Insult in Mice,” MBio 11, no. 1 (2020): e02834–e02919. 31964731 10.1128/mBio.02834-19 PMC6974565

[596] A. Grozić , K. Coker , C. M. Dussik , et al., “Identification of Putative Transcriptomic Biomarkers in Irritable Bowel Syndrome (IBS): Differential Gene Expression and Regulation of TPH1 and SERT by Vitamin D,” PLoS ONE 17, no. 10 (2022): e0275683. 36264926 10.1371/journal.pone.0275683 PMC9584396

[597] Z. Xu , J. J. Chen , Q. Mei , Y. Li , and J. Xu , “Expression of 5‐hydroxytryptamine 7 Receptor in Intestinal Mucosa Correlates With the Degree of Intestinal Inflammation in Crohn's disease,” BMC Gastroenterology [Electronic Resource] 22, no. 1 (2022): 457. 36380275 10.1186/s12876-022-02513-5 PMC9667612

[598] M. Ghiboub , R. S. Boneh , B. Sovran , et al., “Sustained Diet‐Induced Remission in Pediatric Crohn's Disease Is Associated with Kynurenine and Serotonin Pathways,” Inflammatory Bowel Diseases 29, no. 5 (2023): 684–694. 36637175 10.1093/ibd/izac262 PMC10152286

[599] C. Cirillo , J. Tack , and P. Vanden Berghe , “Nerve Activity Recordings in Routine human Intestinal Biopsies,” Gut 62, no. 2 (2013): 227–235. 22387530 10.1136/gutjnl-2011-301777

[600] C. R. Manzella , D. Jayawardena , W. Pagani , et al., “Serum Serotonin Differentiates between Disease Activity States in Crohn's Patients,” Inflammatory Bowel Diseases 26, no. 10 (2020): 1607–1618. 32844174 10.1093/ibd/izaa208 PMC7500525

[601] P. Grieco and I. Gomez‐Monterrey , “Natural and Synthetic Peptides in the Cardiovascular Diseases: An Update on Diagnostic and Therapeutic Potentials,” Archives of Biochemistry and Biophysics 662 (2019): 15–32. 30481494 10.1016/j.abb.2018.11.021

[602] L. Wei , R. R. Warburton , I. R. Preston , et al., “Serotonylated Fibronectin Is Elevated in Pulmonary Hypertension,” American Journal of Physiology. Lung Cellular and Molecular Physiology 302, no. 12 (2012): L1273–L1279. 22523280 10.1152/ajplung.00082.2012 PMC3379044

[603] Y. Ban , T. Watanabe , A. Miyazaki , et al., “Impact of Increased Plasma Serotonin Levels and Carotid Atherosclerosis on Vascular Dementia,” Atherosclerosis 195, no. 1 (2007): 153–159. 17049533 10.1016/j.atherosclerosis.2006.09.005

[604] Y. Xia , D. Wang , N. Zhang , Z. Wang , and L. Pang , “Plasma Serotonin Level Is a Predictor for Recurrence and Poor Prognosis in Colorectal Cancer Patients,” Journal of Clinical Laboratory Analysis 32, no. 2 (2018): e22263. 28543924 10.1002/jcla.22263 PMC6816875

[605] G. K. Fröberg , R. Lindberg , M. Ritter , and K. Nordlind , “Expression of Serotonin and Its 5‐HT1A Receptor in Canine Cutaneous Mast Cell Tumours,” Journal of Comparative Pathology 141, no. 2‐3 (2009): 89–97. 19446835 10.1016/j.jcpa.2008.08.002

[606] A. Fröbe , L. Čičin‐Šain , G. Jones , et al., “Plasma Free Serotonin as a Marker for Early Detection of Breast Cancer Recurrence,” Anticancer Research 34, no. 3 (2014): 1167–1169. 24596355

[607] J. M. Zuetenhorst and B G. Taal , “Metastatic Carcinoid Tumors: A Clinical Review,” The Oncologist 10, no. 2 (2005): 123–131. 15709214 10.1634/theoncologist.10-2-123

[608] A. P. AhYoung , S. C. Eckard , A. Gogineni , et al., “Neutrophil Serine Protease 4 Is Required for Mast Cell‐dependent Vascular Leakage,” Communications Biology 3, no. 1 (2020): 687. 33214666 10.1038/s42003-020-01407-0 PMC7677402

[609] M. Bernardes , T. Vieira , R. Lucas , et al., “Serum Serotonin Levels and Bone in Rheumatoid Arthritis Patients,” Rheumatology International 37, no. 11 (2017): 1891–1898. 28993870 10.1007/s00296-017-3836-9

[610] A. Al‐Sharman , H. M. Al‐Khazaaleh , H. Khalil , A. Aburub , and K. El‐Salem , “The Relationship between Sleep Quality, Sleep‐Related Biomarkers, and Motor Skill Acquisition in People with Multiple Sclerosis: A Pilot Study,” Physical Therapy 101, no. 10 (2021): pzab175. 34270772 10.1093/ptj/pzab175

[611] K. Khoshnevisan , M. Chehrehgosha , S. M. Sajjadi‐Jazi , and A M. Meftah , “Tryptophan and Serotonin Levels as Potent Biomarkers in Diabetes Mellitus Complications: A New Approach of Diagnostic Role,” Journal of Diabetes & Metabolic Disorders 21, no. 2 (2022): 1923–1934. 36404840 10.1007/s40200-022-01096-y PMC9672275

[612] S. Yubero‐Lahoz , P. Robledo , M. Farré , and R. de laTorre , “Platelet SERT as a Peripheral Biomarker of Serotonergic Neurotransmission in the central Nervous System,” Current Medicinal Chemistry 20, no. 11 (2013): 1382–1396. 23409709 10.2174/0929867311320110003

[613] F. De Vadder , E. Grasset , L. Mannerås Holm , et al., “Gut Microbiota Regulates Maturation of the Adult Enteric Nervous System via Enteric Serotonin Networks,” PNAS 115, no. 25 (2018): 6458–6463. 29866843 10.1073/pnas.1720017115 PMC6016808

[614] A. Agus , J. Planchais , and H. Sokol , “Gut Microbiota Regulation of Tryptophan Metabolism in Health and Disease,” Cell Host & Microbe 23, no. 6 (2018): 716–724. 29902437 10.1016/j.chom.2018.05.003

